# An improved repertoire of splicing variants and their potential roles in Arabidopsis photomorphogenic development

**DOI:** 10.1186/s13059-022-02620-2

**Published:** 2022-02-09

**Authors:** Chun-Kai Huang, Wen-Dar Lin, Shu-Hsing Wu

**Affiliations:** 1 Institute of Plant and Microbial Biology, Academia Sinica, 128, Sec. 2, Academia Rd., Taipei, 11529 Taiwan; 2 The Bioinformatics Core Lab, Institute of Plant and Microbial Biology, Academia Sinica, Taipei, 11529 Taiwan

**Keywords:** Alternative splicing, BBX transcription factor, Full-length transcriptome, Iso-seq, Photomorphogenesis, Skotomorphogenesis

## Abstract

**Background:**

Light switches on the photomorphogenic development of young plant seedlings, allowing young seedlings to acquire photosynthetic capacities and gain survival fitness. Light regulates gene expression at all levels of the central dogma, including alternative splicing (AS) during the photomorphogenic development. However, accurate determination of full-length (FL) splicing variants has been greatly hampered by short-read RNA sequencing technologies.

**Result:**

In this study, we adopt PacBio isoform sequencing (Iso-seq) to overcome the limitation of the short-read RNA-seq technologies. Normalized cDNA libraries used for Iso-seq allows for comprehensive and effective identification of FL AS variants. Our analyses reveal more than 30,000 splicing variant models from approximately 16,500 gene loci and additionally identify approximately 700 previously unannotated genes. Among the variants, approximately 12,000 represent new gene models. Intron retention (IR) is the most frequently observed form of variants, and many IR-containing AS variants show evidence of engagement in translation. Our study reveals the formation of heterodimers of transcription factors composed of annotated and IR-containing AS variants. Moreover, transgenic plants overexpressing the IR forms of two B-BOX DOMAIN PROTEINs exhibits light-hypersensitive phenotypes, suggesting their regulatory roles in modulating optimal light responses.

**Conclusions:**

This study provides an accurate and comprehensive portrait of full-length transcript isoforms and experimentally confirms the presence of de novo synthesized AS variants that impose regulatory functions in photomorphogenic development in Arabidopsis.

**Supplementary Information:**

The online version contains supplementary material available at 10.1186/s13059-022-02620-2.

## Background

Light signals are perceived through different photoreceptors, including phytochromes, cryptochromes, phototropins, and UVB-RESISTANCE 8 [1, 2]. During photomorphogenic development, light triggers the inhibition of hypocotyl elongation, apical hook and cotyledon opening, and the initiation of photoautotrophic development [3, 4]. These physiological changes are the result of light-induced transcriptome and translatome shifts by gene expression regulation at the transcriptional, post-transcriptional, translational, and post-translational levels [review see [5]].

Light triggers the translocation of PHYTOCHROME A and PHYTOCHROME B into the nucleus to attenuate negative regulators of photomorphogenesis, PHYTOCHROME INTERACTING FACTORs (PIFs) [6–8], and to promote the expression of positive photomorphogenic regulators such as ELONGATED HYPOCOTYL 5 (HY5), B-BOX DOMAIN PROTEIN 21 (BBX21), and BBX22/LIGHT-REGULATED ZINC FINGER PROTEIN 1 (LZF1) [9–11]. Light also induces the translocation of CONSTITUTIVE PHOTOMORPHOGENIC1 (COP1), a central repressor of photomorphogenesis, from the nucleus to the cytosol, resulting in the stabilization of the positive photomorphogenic regulators HY5, BBX21, and BBX22/LZF1 [12–16]. BBX20, BBX21, and BBX22 are essential partners of HY5 to promote photomorphogenesis in Arabidopsis [17]. Other BBX members, such as BBX24 and BBX25, function as negative regulators to fine-tune photomorphogenesis by interacting with HY5 to downregulate the expression of *BBX22* [18]. The complex interplay of both positive and negative transcriptional regulators shapes transcriptional activation and repression in response to the intensity and quality of light signals.

In addition to transcriptional activation or repression, alternative selection of transcription start/end sites (alt-TSSs or alt-TESs) and alternative polyadenylation (APA) sites contribute to the transcriptomic complexity. The genome-wide study of red light-regulated alt-TSSs in Arabidopsis de-etiolating seedlings reported that alt-TSS affects the N-terminal coding sequence of proteins, thus leading to altered subcellular localization of proteins and their biological functions [19]. In contrast, APA represents the dominant class of the 3′ transcript variants that can contribute to the stability and translation efficiency of a transcript. When an APA event occurs in an exon or an intron 5′ to the stop codon, the resulting transcript variant can produce a C-terminal truncated protein isoform with altered subcellular localization and/or biological functions [20–23].

At the post-transcriptional level, nascent pre-mRNA undergoes several processing steps to generate mature mRNA. Alternative splicing (AS) is an important post-transcriptional regulatory mechanism ensuring transcriptome plasticity to achieve optimal plant development and proper responses to environmental stimuli [24, 25]. In Arabidopsis, more than 60% of multi-exon genes undergo AS, and many of these genes can generate multiple splicing variants [26, 27]. Only sporadic studies reported the biological functions of these AS variants in light responses and photomorphogenesis [28–33].

In response to various internal and external stimuli, splicing regulators or splicing factors function to determine the AS site usage, leading to production of AS variants [25, 34]. Splicing regulators reported to control pre-mRNA processing during photomorphogenesis include REDUCED RED-LIGHT RESPONSES IN CRY1CRY2 BACKGROUND 1, SNW/SKI-INTERACTING PROTEIN, SPLICING FACTOR FOR PHYTOCHROME SIGNALING, heterogeneous nuclear ribonucleoprotein H1, and pre-mRNA-processing factor 39-1 [35–41]. These splicing factors/regulators help seedlings adapt to various light environments by modulating the production of transcript variants, their relative abundance, and diversity [review see [42]].

The comprehensive FL transcriptomes have allowed for the identification of 5′ and 3′ regulatory elements and an estimation of proteome complexity. Post-transcriptional regulations via alt-TSS, APA, and AS in most genomic studies were mostly inferred by short-read RNA sequencing. However, because of the limitation of sequencing length, short-read sequencing typically loses the actual configurations of the transcript variants, thereby prohibiting an accurate determination of FL transcript isoforms. PacBio SMRT and Nanopore sequencing techniques offer the advantage of long-read sequencing. PacBio SMRT sequencing has been used for direct observation of FL transcripts, with lower sequencing error rates for constituting highly accurate FL transcriptomes [43–48].

In the present study, we used PacBio isoform sequencing (Iso-seq) to assess the impact of AS on the transcriptomic shift for one of the key development processes in plants, photomorphogenesis. The successful integration of normalized cDNA libraries and Iso-seq allowed us to achieve unprecedented comprehensiveness of transcriptomic analyses in de-etiolating Arabidopsis seedlings. We obtained a high complexity of FL transcriptomes with fully annotated AS, TSSs/TESs, and APAs and identified ~ 12,000 new gene models and ~ 700 unannotated genes in this important developmental process. We also experimentally validated that AS variants of selected transcription factors can function as positive or negative regulators of their annotated cognate partners to ensure optimal light responses during photomorphogenic development.

## Results

### A strategic combination of normalized cDNA libraries and Iso-seq maximized the identification of full-length cDNAs in de-etiolating Arabidopsis seedlings

To maximize the identification of FL transcriptomes in early de-etiolating seedlings, we used PacBio Iso-seq with normalized cDNA libraries created with RNA isolated from 4-day-old etiolated seedlings treated with 4-h dark (D4h) or 4-h white light (L4h) (Additional file 1: Fig. S1). By normalizing the cDNAs, we could avoid redundant sequencing of abundant transcripts and therefore increase the representation of rare transcripts. The efficiency of cDNA normalization was confirmed by the markedly reduced representation of *UBQ10* (an abundant transcript) in both the normalized D4h and L4h cDNA libraries (Additional file 1: Fig. S2). Large cDNAs could be well represented by performing Iso-seq with size-fractionated cDNA libraries (1–2 and 2–4 kb) (Additional file 1: Fig. S3).

We obtained a total of 411,305 and 380,525 read-of-inserts (ROIs) from D4h and L4h samples, respectively. Additional file 2: Table S1 lists the summarized statistics of Iso-seq results. A data-filtering pipeline was used to identify FL transcript reads as shown in Additional file 1: Fig. S4 and detailed in “Methods”. We obtained a total of 136,643 and 168,454 FL non-chimeric reads from D4h and L4h samples, respectively (Table 1). These reads corresponded to > 16,000 gene loci, comparable to the number of expressed genes reported previously [49].

**Table 1:** Summary of D4h and L4h Iso-seq

cDNA library	FL-NC reads^a^	HQ FL reads^b^	80% HQ FL reads	Gene^c^	Novel transcripts^d^	Splicing isoforms	APA sites^e^
**Iso-seq_D4h**	**136,643**	**73,746**	**57,833**	**14,176**	**392**	**20,805**	**26,179**
**Iso-seq_L4h**	**168,454**	**88,303**	**69,636**	**14,821**	**398**	**23,345**	**28,951**

^a^FL non-chimeric reads (FL-NC) as described in “Methods”

^b^High-quality FL reads (HQ FL) as described in “Methods”

^c^Number of gene loci mapped by 80% HQ FL reads to TAIR10

^d^Novel transcripts: unannotated in TAIR10

^e^Alternative polyadenylation (APA) sites

**Fig. 1:**
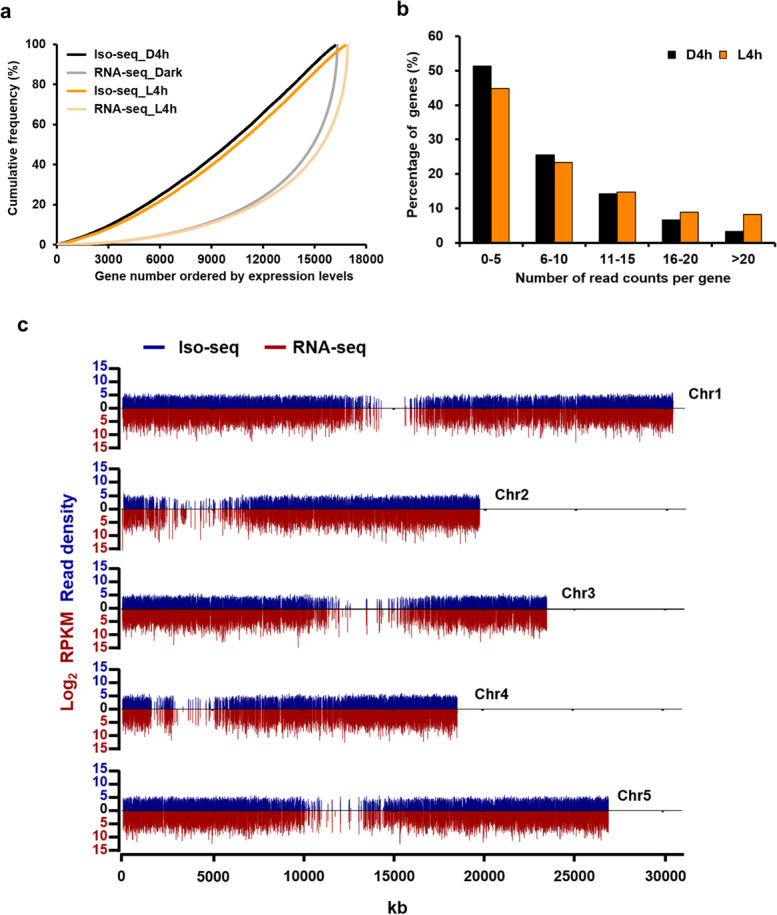
Normalization efficiencies and read coverage of normalized D4h and L4h Iso-seq FL datasets. **a** Cumulative frequencies of gene expression levels in Iso-seq (this study) and RNA-seq datasets (49). **b** Frequency of read counts per gene in D4h and L4h Iso-seq datasets. **c** Distribution of Iso-seq reads (read densities) and RNA-seq expression (log_2_RPKM) in 5-kb windows along the 5 Arabidopsis chromosomes

A few attributes also indicated the success of cDNA normalization. First, the top 10 most frequently sampled transcripts in our Iso-seq only represented 0.3% of total FL reads, despite their medium to high expression levels observed in a previous RNA-seq data from samples at the same developmental stages [49] (Additional file 3: Table S2). Second, when the cumulative frequencies were plotted against the expression levels (in read counts), linear curves were observed for Iso-seq results, whereas exponential curves were seen for RNA-seq data, skewed by the highly expressed transcripts (Fig. 1a). Third, when the numbers of read counts per gene locus were tabulated, those with ≤ 5 FL read counts per gene represented at least 45% of mapped FL reads (Fig. 1b). Fourth, when the read counts for Iso-seq and RNA-seq data were plotted onto the 5 Arabidopsis chromosomes, the Iso-seq reads were more evenly distributed than those from RNA-seq (Fig. 1c).

Taken together, the normalized cDNA libraries used in our Iso-seq studies greatly compensated for the relatively lower throughput of PacBio Iso-seq technology by reducing the read counts of highly expressed genes for effective detection of rare transcripts and maximizing gene coverage.

### Expressed genes were broadly represented in the Iso-seq transcriptomes

By clustering the FL non-chimeric reads, we obtained 73,746 (D4h) and 88,303 (L4h) high-quality (HQ) FL reads (Table 1). Three major types of errors associated with Iso-seq were assessed by aligning the HQ FL reads to the TAIR10 genome sequence. A ~ 0.1% error rate per nucleotide was observed (Additional file 1: Fig. S5), which was considerably lower than from previous Iso-seq studies of sorghum and humans [46, 50].

**Fig. 2:**
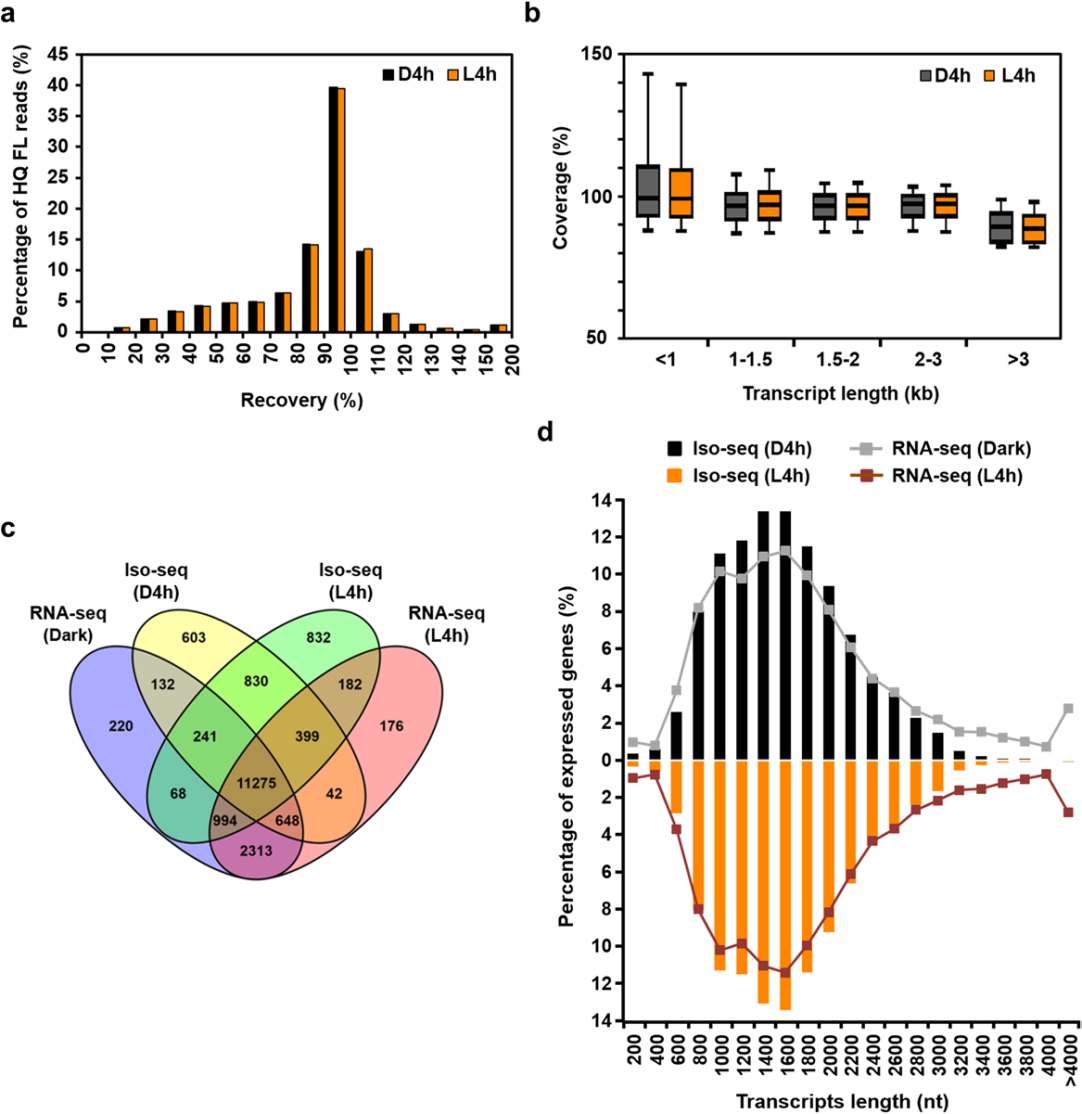
Distribution of transcript and expressed transcriptome coverages. **a** Frequency plot for the relative coverage of the D4h and L4h HQ FL reads against the transcripts annotated in TAIR10. **b** Box plots showing the coverage distribution of D4h and L4h HQ FL reads by transcript length. Boxes show medium values and 25–75 percentile range with whiskers indicating the 10–90 percentile range. **c** Venn diagram showing shared and distinct transcripts detected by RNA-seq (49) and Iso-seq datasets. **d** Iso-seq and RNA-seq data (49) have comparable coverage of expressed transcripts of different lengths

By aligning the HQ FL reads with the transcript models annotated in TAIR10, 40% of HQ FL reads in both D4h and L4h samples overlapped 90–100% with the annotated transcripts and ~ 20% exceeded the annotated lengths (Fig. 2a). Most had sequence extensions in the 5′ and/or 3′ ends of currently annotated transcripts, but AS accounted for > 3000 longer transcripts in both D4h and L4h datasets (Additional file 4: Table S3).

For the residual 40% of HQ FL reads that were shorter than the annotated transcript models, we analyzed their 5′ and 3′ ends relative to the transcript start site (TSS) and transcript end site (TES) of the annotated transcripts. Some HQ FL reads missed part of the 5′ end but not 3′ end of reads (Additional file 1: Fig. S6), possibly because of partially truncated transcripts, alternative TSS selection, or incomplete reverse transcription during cDNA synthesis. When 80% coverage rate was applied to filter the reads, the inclusion of these HQ FL reads with 5′ end truncation was greatly minimized, as shown by the highly superimposed plots of Iso-seq reads and annotated transcripts at both the TSS and TES (Additional file 1: Fig. S6). Most of these reads matched or had 5′ extensions as compared with the 5′ end revealed by nanoPARE sequencing [51] (Addition file 1: Fig. S7), therefore with high degree of 5′ end completeness. These reads have high sequence coverage across different sizes of annotated transcripts (Fig. 2b), with 96% mean coverage for transcripts < 1, 1–1.5, 1.5–2, and 2–3 kb, and 88% for transcripts > 3 kb.

The 80% HQ FL reads (57,833 and 69,636 for D4h and L4h, respectively) were used for further analyses and represented a total of 14,176 (D4h) and 14,821 (L4h) genes (Table 1). To determine whether our Iso-seq results had good coverage of the expressed transcriptome, we compared our data with the expressed genes defined previously by short-read RNA-seq [49]. The Venn diagram in Fig. 2c shows that genes identified by Iso-seq or RNA-seq largely overlapped (~ 80%), with some genes showing evidence of expression revealed only by Iso-seq (2265) or RNA-seq (2709) (Fig. 2c). Genes identified only by Iso-seq of normalized cDNAs were those with low expression levels in our previous study [49], similar to the detection of low-expressed transcripts by normalizing cDNAs described recently [52]. The overall size distribution of expressed genes identified by Iso-seq or RNA-seq was largely similar between the two methods (Fig. 2d). However, for transcripts < 800 bp or > 3000 bp, expressed genes identified by Iso-seq had slightly lower coverage than those by RNA-seq (Fig. 2d), possibly because of size selection during library construction or sequencing bias.

Taken together, our Iso-seq HQ FL reads constituted a comprehensive repertoire of FL transcriptomes and expanded the 5′/3′ cDNA ends for genes expressed in early de-etiolating Arabidopsis seedlings.

### Construction of AS FL gene models by Iso-seq results

Processed Iso-seq reads allowed us to construct gene models for splicing variants of any given gene with high confidence. The reads were first mapped to the TAIR10 genome to detect splicing junction sites for creating gene models. Reads that differed only in the first or last exon were considered redundant. The redundant reads were clustered, and only the longest version was retained to create splicing gene models. We classified the resulting splicing gene models into three groups: TAIR10 gene models, Iso-seq gene models, and TAIR10 unannotated genes. TAIR10 gene models are reads matching TAIR10 annotated transcripts at all splice junctions (18,343 in this study) (Additional file 5: Table S4). Those with at least one splice junction difference from the TAIR10 gene models were designated Iso-seq gene models (11,902 in this study) (Additional file 5: Table S4). Finally, the Iso-seq reads mapped to the previously unannotated genomic regions were defined as TAIR10 unannotated genes (Additional file 6: Table S5).

**Fig. 3:**
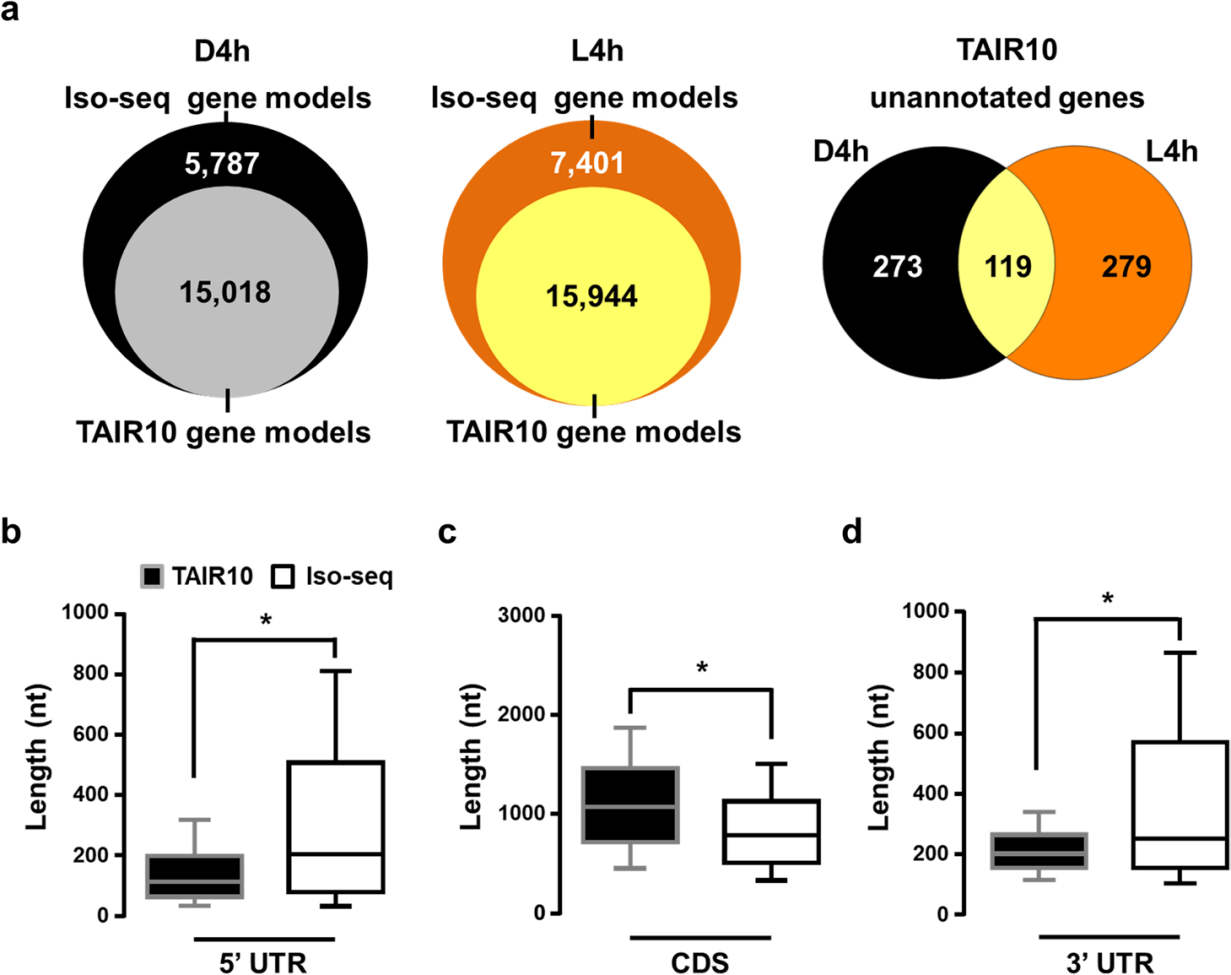
Comparisons of gene models identified by Iso-seq and those annotated in TAIR 10. **a** Number of genes in TAIR10, Iso-seq, and novel (TAIR-10 unannotated) gene models. Results of gene models from D4h and L4h Iso-seq datasets were plotted separately. **b–d** Box plots showing the 5′ UTR length (**b**), the coding sequence (CDS) length (**c**), and the 3′ UTR length (**d**) distribution of transcripts identified by Iso-seq and annotated in TAIR10. The diagrams compare lengths of gene structure of TAIR10 annotated gene models (*n* = 18,343) and Iso-seq gene models (*n* = 11,902). Boxes show medium values and 25–75 percentile range with whiskers indicating the 10–90 percentile range. * *p* < 0.001 (Kolmogorov-Smirnov test)

A total of 21,197 and 23,743 gene models were identified in D4h and L4h datasets, respectively (Fig. 3a, Additional file 5: Table S4 and Additional file 6: Table S5). Among them, ~ 30% were Iso-seq gene models and 671 genes were previously unannotated in TAIR10 (Fig. 3a). The authenticity of the gene models derived in this study was cross validated by comparison with long-read datasets by Pacbio Iso-seq [53] or Nanopore direct RNA sequencing [54]. By adopting the same mapping and processing pipeline, 13,493 and 75,096 gene models were generated from these two datasets (Additional file 7: Table S6), respectively. Pair-wise comparison showed that 13,284 (72.4%) of TAIR10 gene models and 1462 (12.3%) of the Iso-seq and novel gene models combined in this study have counterparts in at least 1 of the 2 previous datasets (Addition file 1: Fig. S8, and Additional file 7: Table S6). Most gene models (29,250; ~ 95%) reported in this study can also be reconstructed based on short-read RNA-seq data generated from the same developmental stages [49] by using cufflinks (Addition file 1: Fig. S9, and Additional file 8: Table S7).

We also examined whether the unique AS events of TAIR10 unannotated gene models identified in this study possess the canonical dinucleotide feature (GU-AG) in the splicing junctions. Results showed that 79.4% of our Iso-seq gene models have canonical splicing sites (GU-AG), comparable to 89.4% for RNA-seq data from normalized cDNA libraries [27] and 88.9% for a Pacbio Iso-seq dataset [53], and significantly higher than the ~ 31% for the Nanopore direct RNA sequencing dataset [54] (Addition file 1: Fig. S10). The low ratio of canonical splicing sites in Nanopore gene models is likely a result of uncorrected sequencing errors as discussed previously [48, 53, 54]. Taken together, high-quality gene models derived in our study significantly expanded the complexity of the expressed transcriptomes during the de-etiolation process.

We next analyzed whether sequence features differentiated the TAIR10 and Iso-seq gene models by comparing their length distribution in the 5′ untranslated regions (UTRs), coding sequences (CDS), and 3′ UTRs. The median length of 5′ UTR and 3′ UTR was slightly but significantly longer for Iso-seq than TAIR10 gene models, but Iso-seq gene models had shorter CDS, which suggests that the genes will encode shorter polypeptides than those from TAIR10 gene models (Fig. 3b–d).

### Novel transcripts revealed by Iso-seq

The mean length of the 671 unannotated transcripts identified by our Iso-seq results was 962 bp, with the longest and shortest transcripts being 2796 and 332 bp, respectively (Additional file 6: Table S5). When we mapped short-read RNA-seq data of the same developmental stages from a previous study [49] to these novel loci, the mean expression was significantly greater for these novel loci (3.09 and 2.50 reads per kilobase of transcript, per million mapped reads [RPKM] for D4h and L4h, respectively) than those of the intergenic region (0.18 and 0.16 RPKM for D4h and L4h, respectively) (Additional file 6: Table S5), so these novel loci are indeed transcribed in these developmental stages. Among the 671 novel transcripts, 122 were also detected in RNA prepared from 2-week-old Arabidopsis by Nanopore sequencing [54].

We next examined whether these unannotated transcripts are unique in Arabidopsis or conserved in additional plant species by using a blastn search against reference genome sequences in NCBI. With the threshold of e-value ≤ 10^−50^, 279 of the 671 novel transcripts (42%) had at least one significant match by blastn, which suggests their authenticity as previously undescribed genes in the Arabidopsis genome. Additional file 6: Table S5 lists the best match result for each gene model.

**Fig. 4:**
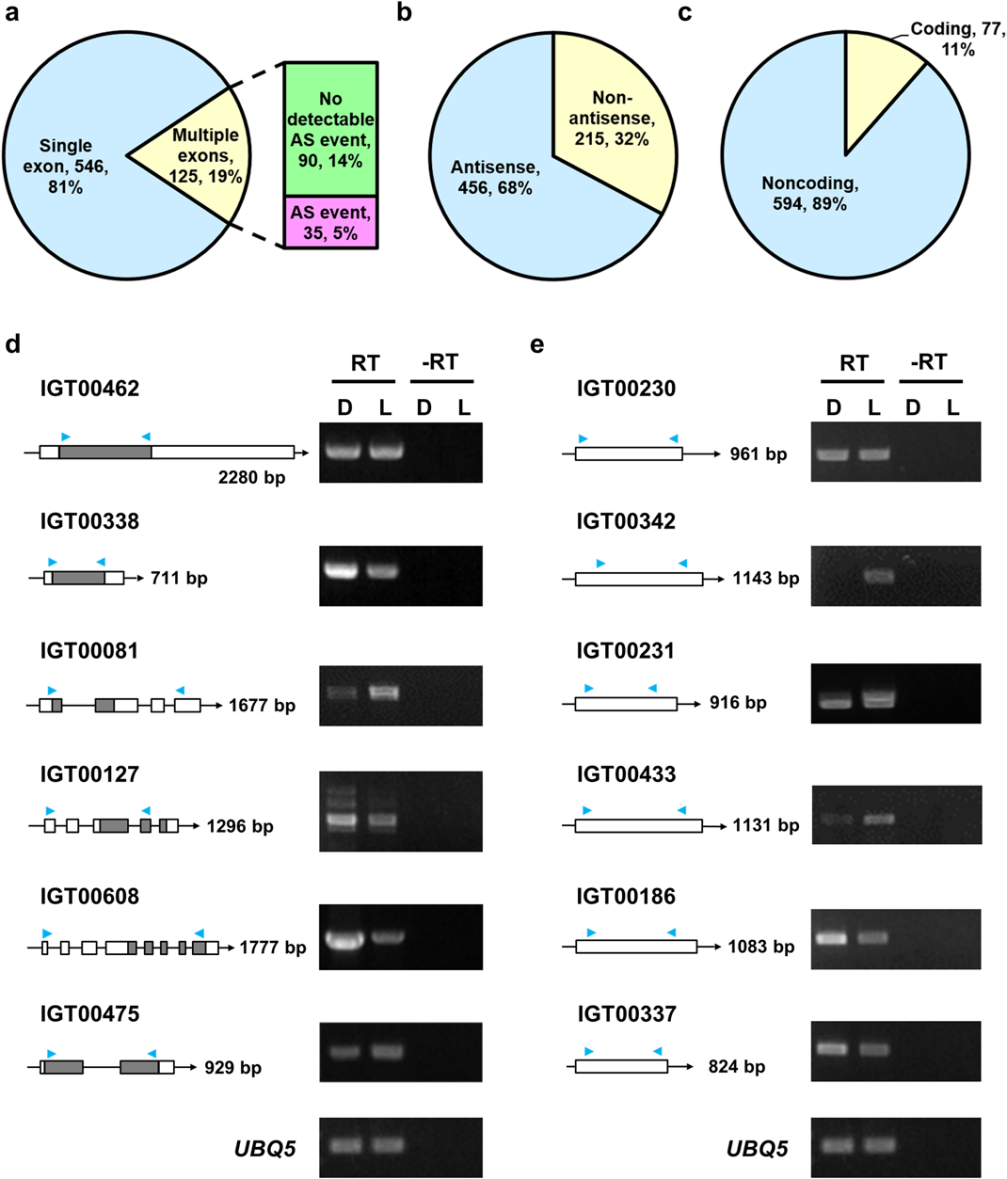
Classification and validation of the novel transcripts identified by Iso-seq. **a** Pie chart showing percentages of novel transcripts with single or multiple exons, with or without detectable alternative splicing events. **b** Pie chart showing percentage of novel transcripts from the antisense strand of annotated genes. **c** Pie chart showing novel transcripts that are likely non-coding or with coding potentials. Those with coding potential scores (> 0) calculated by Coding Potential Calculator 2 software were considered coding. **d, e** RT-PCR validation of novel transcripts with coding potential (**d**) and non-coding novel transcripts (**e**). The left panels show diagrams of selected transcripts. White boxes, gray boxes, and lines represent the untranslated regions, CDS, and introns, respectively. Forward and reverse primers for PCR are showed as blue arrowheads. Right panels: gel images showing amplified RT-PCR products from D4h (D) and L4h (L) cDNAs. RT and -RT, reverse transcription to produce cDNA with or without reverse transcriptase, respectively. *UBQ 5* was a RT-PCR control

Among these 671 novel genes, 546 (81%) had single exons and 125 (19%) multiple exons (Fig. 4a). AS variants were found for 35 (5%) of the multi-exon novel gene models (Fig. 4a). Close to 70% (456) of these novel transcripts were derived from the antisense strands of previously annotated transcripts (Fig. 4b and Additional file 6: Table S5). Among the remaining 215 novel transcripts, 6 matched miRNAs from a blast search of the miRNA stem-loop sequences at miRBase (Additional file 6: Table S5).

The coding potential of these novel gene models was assessed by using Coding Potential Calculator 2 [55]: 594 (89%) likely represent non-coding transcripts and 77 (11%) have the potential to encode protein products (Fig. 4c). Approximately half of the 77 predicted coding transcripts can encode proteins with orthologs found in the other plant species according to a blastx search (Additional file 6: Table S5), which suggests that these are bona fide transcripts conserved during the evolution of land plants.

Among the transcripts with the high or low coding potential, we selected 6 each to validate their expression in etiolated/de-etiolating seedlings by RT-PCR. Results shown in Fig. 4d, e confirmed the expression of all selected novel transcripts. Of note, some novel transcripts accumulated to different levels in etiolated (D) or de-etiolating (L) seedlings (Fig. 4d, e) as well as in the previous RNA-seq results [49] (Additional file 6: Table S5), which indicates that light imposes regulatory roles in the expression of these novel transcripts.

### Comprehensive analyses of AS, TSSs/TESs, and APAs of FL transcript isoforms in early de-etiolating seedlings

**Fig. 5:**
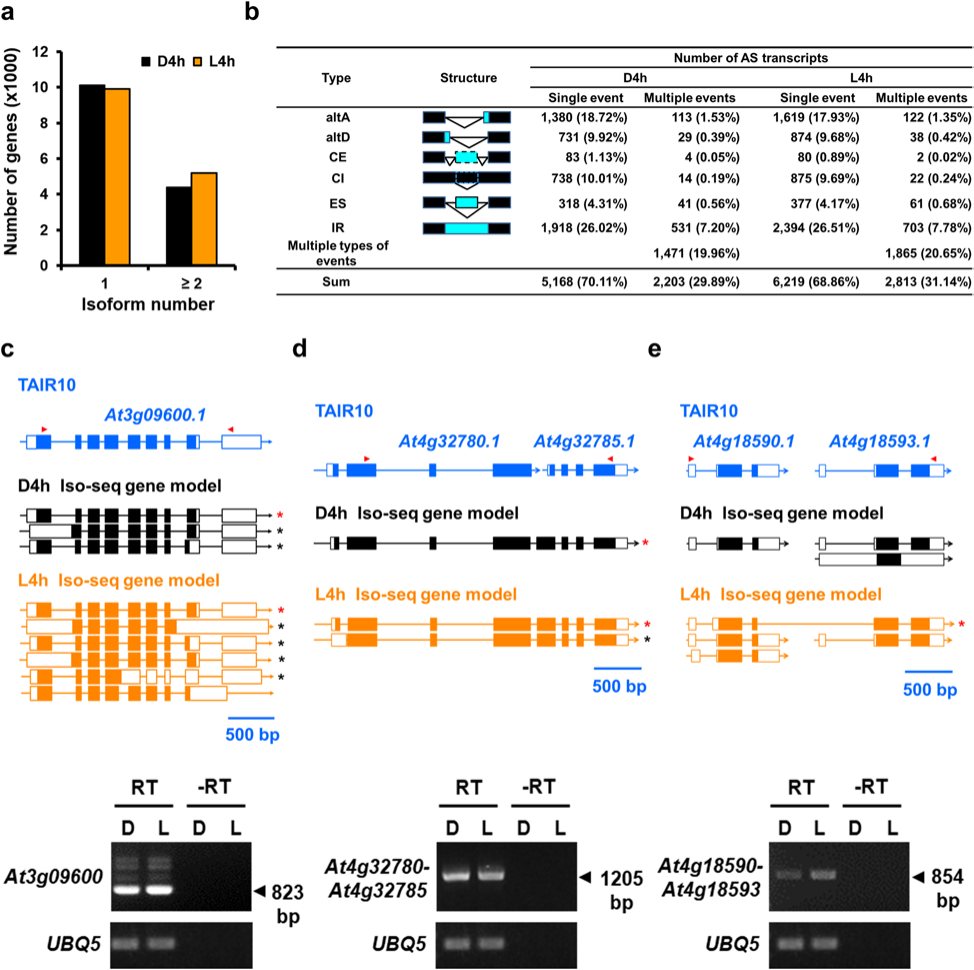
Alternative splicing events revealed by Iso-seq. **a** Number of genes with one or more splicing isoforms in D4h and L4h Iso-seq datasets. **b** Number, frequency, and types of AS events identified by Iso-seq. altA, alternative acceptor site; altD, alternative donor site; CE, cryptic exon; CI, cryptic intron; ES, exon skipping; IR, intron retention. **c–e** Illustration and RT-PCR validation of *At3g09600 (RVE8)* gene models (**c**), and two novel gene models with *At4g32780-At4g32785* fusion transcript (**d**) and *At4g18590*-*At4g18593* fusion transcript (**e**). The representative TAIR10 gene model is shown in blue. D4h or L4H Iso-seq gene models are shown in black and orange, respectively. White boxes, black boxes, and lines represent the untranslated regions, CDS, and introns, respectively. RT-PCR was performed with forward and reverse primers marked with red arrowheads. Sizes of the amplified fragments of *At3g09600* (*RVE8*), *At4g32780-At4g32785*, and *At4g18590-At4g18593* fusion transcripts are 823 bp, 1205 bp, and 854 bp, respectively. AS variants confirmed by Sanger sequencing are marked with asterisks (red or black for TAIR10 or Iso-seq gene model, respectively). RT or -RT represents that RT-PCR with or without reverse transcription. -RT as a RT-PCR negative control. *UBQ 5* as a RT-PCR control. Scale bar, 500 bp

Overall, 20,805 and 23,345 TAIR10 and Iso-seq gene models were detected in D4h and L4h datasets, respectively (Table 1 and Fig. 3a). In the D4h (L4h) datasets, a single transcript was observed for ~ 10,000 (~ 9900) genes, and ~ 4400 (~ 5200) genes producing ≥ 2 AS variants, constituting ~ 11,000 (~ 13,800) splicing variants in the D4h (L4h) dataset (Fig. 5a).

We next analyzed the frequency of occurrence of major types of AS, including intron retention (IR), alternative donor (altD), and alternative acceptor (altA) sites, exon skipping (ES), cryptic intron (CI), and cryptic exon (CE). Approximately 30% of the AS variants had altA or altD sites (Fig. 5b). CE, CI, and ES represented 1%, 10%, and 5% of AS isoforms and IR the most frequently observed AS form (34%) (Fig. 5b). For both D4h and L4h samples, 30% of the AS transcripts had multiple AS events (altA/D, CE/CI, ES, IR, or mixed AS types) (Fig. 5b). Additional file 1: Figure S11 illustrates 19 AS variants for *At3g02600*, representing the highest number of AS variants detected for a single gene by our Iso-seq results. With RT-PCR, we experimentally validated the presence of multiple AS variants derived from *At3g09600*, which encodes a circadian clock-related MYB transcription factor, REVEILLE 8 (RVE8) (Fig. 5c).

A significant number of AS variants from ~ 2000 gene loci had alternative transcription start exon(s) (TSEs) or alternative transcription end exon(s) (TEEs) (Additional file 5: Table S4). Among them, 202 showed evidence of sequences spanning separately annotated transcripts in TAIR10. These were possibly results of alternative transcription start/end sites (TSSs/TESs), transcription readthrough, or mis-annotation in TAIR10. Two observations supported that at least some of these are fusion transcripts. First, RNA-seq data [49] showed junction reads (≥ 10 nt spanning the two neighboring genes) for 106 of the fusion transcripts (Additional file 9: Table S8). Second, according to > 5000 RNA-seq datasets, the coexpression coefficient of the two neighboring genes that potentially produce fusion transcripts were much more prominent than those that do not (Pearson’s correlation, *p* < 0.0001) (Addition file 1: Fig. S12 and Additional file 9: Table S8). Figure 5 d, e show examples of such fusion transcripts spanning *At4g32780* and *At4g32785*, *At4g18590* and *At4g18593*, respectively. RT-PCR with primers corresponding to the fusion transcripts amplified cDNAs of expected sizes (Fig. 5d, e), and the sequences were verified by Sanger sequencing. In addition, these two fusion transcripts can encode protein orthologs with homologs in the other plant species according to a blastx search. Thus, Iso-seq can help improve the current annotation of the Arabidopsis genome.

Alternative polyadenylation (APA) represents another form of gene expression regulation by producing transcript variants with different 3′ ends that can increase the complexity of transcriptomes, impose translation regulation, or change the stability of mRNAs [review see [56]]. Most of the polyadenylation sites (PASs) derived from the 3′ end of D4h and L4h HQ FL reads matched those annotated in TAIR10 (Additional file 1: Fig. S6 and S13) as well as those inferred by direct RNA sequencing (DRS) [57], Nanopore sequencing [54], and Pacbio Iso-seq [53] and those included in the plant alternative polyadenylation sites database (PlantAPAdb) [58]. Overall, 7797 (53.7%) and 8776 (58.1%) genes had two or more polyadenylation sites in D4h and L4h FL datasets, respectively (Additional file 1: Fig. S14a). The mean number of PASs per gene was 2.1 in this study. As an example, 5 APA sites were observed for transcripts derived from *At1g01210* (Additional file 1: Fig. S14b).

When we analyzed the flanking sequences (+/– 50 nt) of the APA sites, similar sequence compositions were observed for D4h and L4h Iso-seq transcripts, with the predominance of A/U, an upstream uracil (U) and downstream adenine (A) relative to the predicted cleavage sites (Additional file 1: Fig. S14c, d). We searched for over-represented sequence features in both the near upstream elements (NUEs) and cleavage elements (CEs) at positions -10 to -25 and -10 to -1 of the PASs, respectively (Additional file 1: Fig. S15a-g). MEME analyses indicated that AAUAAA and UUUUUU were over-represented in the NUEs and CEs, respectively, which is consistent with previous studies in Arabidopsis and rice [59, 60].

In summary, our Iso-seq datasets benefited the genome-wide identification of FL transcript isoforms resulting from AS, choice of TSSs/TESs or APAs. This work greatly expanded the transcriptome diversity and offers a wealth of sequence resources for future mechanistic studies of transcript processing, structure, stability, and their contributions to the de-etiolation process.

### Light regulates the expression of both annotated and IR variants of key transcription factors

Although premature termination codons are commonly seen in AS variants, recent studies indicated that thousands of AS transcripts do not elicit nonsense-mediated decay (NMD) [61] and AS transcripts were found to associate with the translation machinery polysomes [62, 63]. This observation implied that AS isoforms could encode protein variants to expand the proteome repertories. Indeed, protein variants encoded by AS transcripts were reported to exert regulatory functions in plant development [28, 64, 65].

**Fig. 6:**
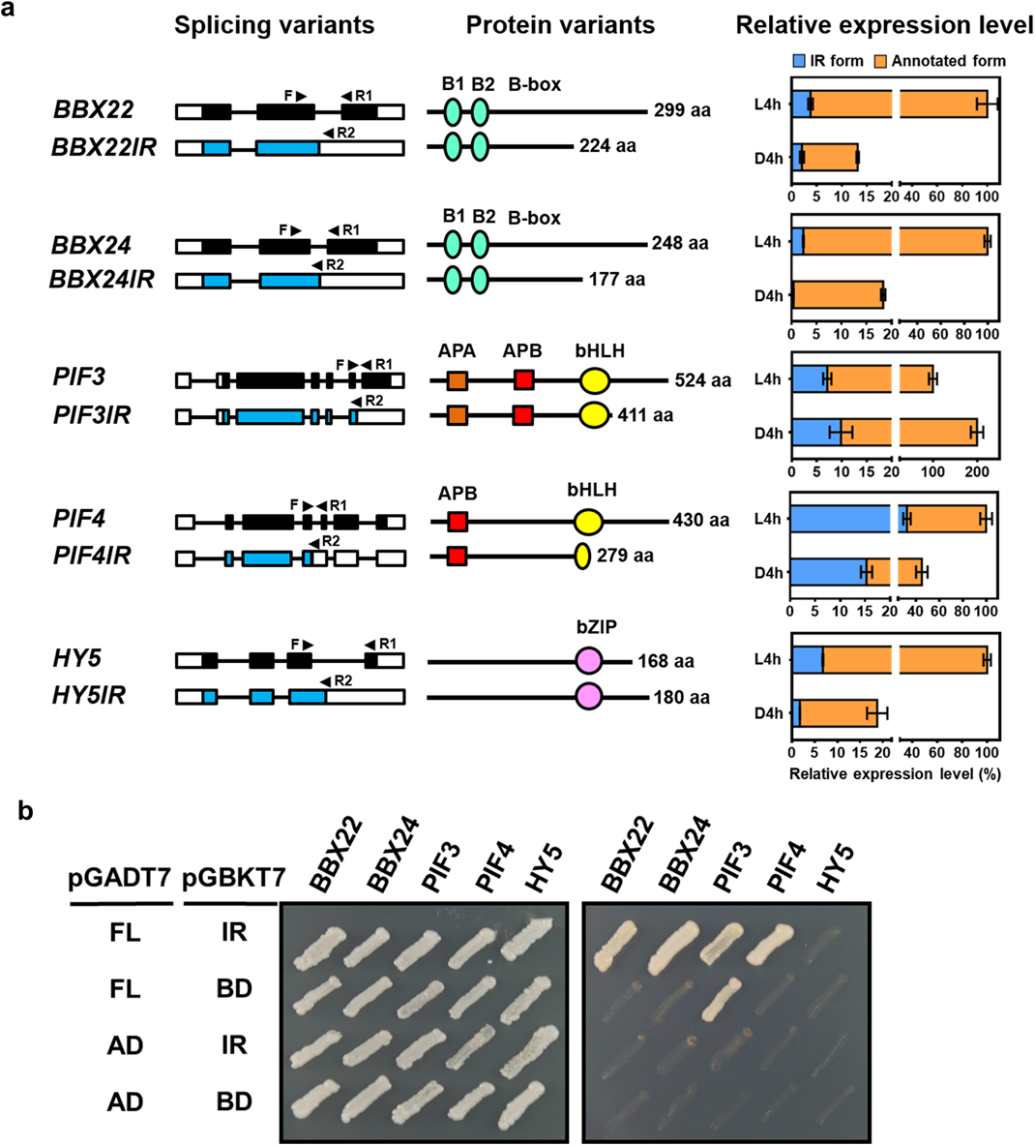
Expression and protein–protein interaction of annotated and IR isoforms of light-regulated transcription factors. **a** Illustrations of mRNA and protein isoforms for light-regulated transcription factors in annotated (black) and isoforms with retained intron (IR) (blue). Right panel shows the relative expression of annotated and IR transcripts in D4h and L4h samples. The 5 IR transcripts of the light-regulated transcription factors *BBX22IR*, *BBX24IR*, *PIF3IR*, *PIF4IR*, and *HY5IR* were selected. White boxes, filled boxes, and lines represent the untranslated regions, coding sequences (CDS), and introns, respectively. Forward and reverse primers are marked with arrowheads. Recognizable domains of the deduced proteins are marked. Total transcript levels for each transcript in the L4h sample were set to 100%. **b** Protein–protein interaction analyses of 5 light-regulated transcription factors between annotated and their IR forms by yeast two-hybrid assays

To assess whether AS variants constitute regulatory roles in photomorphogenic development, we focused on loci encoding transcription factors (TFs) with IR variants (the most prevalent AS form) and with variants showing evidence of sequences protected by ribosomes in the early stage of Arabidopsis photomorphogenesis [49]. In total, ribosome-protected IR events were observed for transcripts from 212 TFs in etiolated or de-etiolating seedlings (Additional file 10: Table S9). We analyzed 5 IR variants, *BBX22IR*, *BBX24IR*, *PIF3IR*, *PIF4IR*, and *HY5IR*, because their annotated counterparts encode TFs of known regulatory roles in photomorphogenesis [5]. Structures of both the transcript and protein variants for these 5 TFs are shown in Fig. 6a. The predicted functional domains are also marked. Except for HY5IR, all IR variants encode truncated protein products (Fig. 6a). The relative expression of the annotated and IR transcripts in etiolated and de-etiolating seedlings was determined by quantitative RT-PCR. Light enhanced both the annotated and IR transcripts for *BBX22*, *BBX24*, *PIF4*, and *HY5* but not *PIF3* (Fig. 6a).

### Heterodimerization of FL and IR variants

TFs commonly function as homo- or heterodimers. We hypothesized that a TF IR variant could form a heterodimer with its annotated FL protein to impose regulatory roles. This was first tested by yeast two-hybrid assays, which found physical interactions for BBX22 and BBX22IR, BBX24 and BBX24IR, PIF3 and PIF3IR, and PIF4 and PIF4IR protein pairs. The protein pair of PIF3 and BD showed auto-activation activities and was not further pursued (Fig. 6b). No detectable interactions were detected for HY5 and HY5IR even though both proteins were successfully expressed (Fig. 6b and Additional file 1: Fig. S16).

### Functional studies of BBX22IR and BBX24IR in photomorphogenic development

The above results showed that truncated proteins, BBX22IR, BBX24IR, and PIF4IR, can interact with their FL counterparts and may play regulatory roles in photomorphogenic development. We next characterized the biological functions of BBX22IR and BBX24IR by overexpressing HA-tagged BBX22IR and BBX24IR in wild-type Arabidopsis. The expression of HA-BBX22IR and HA-BBX24IR in transgenic lines was confirmed (Additional file 1: Fig. S17).

**Fig. 7:**
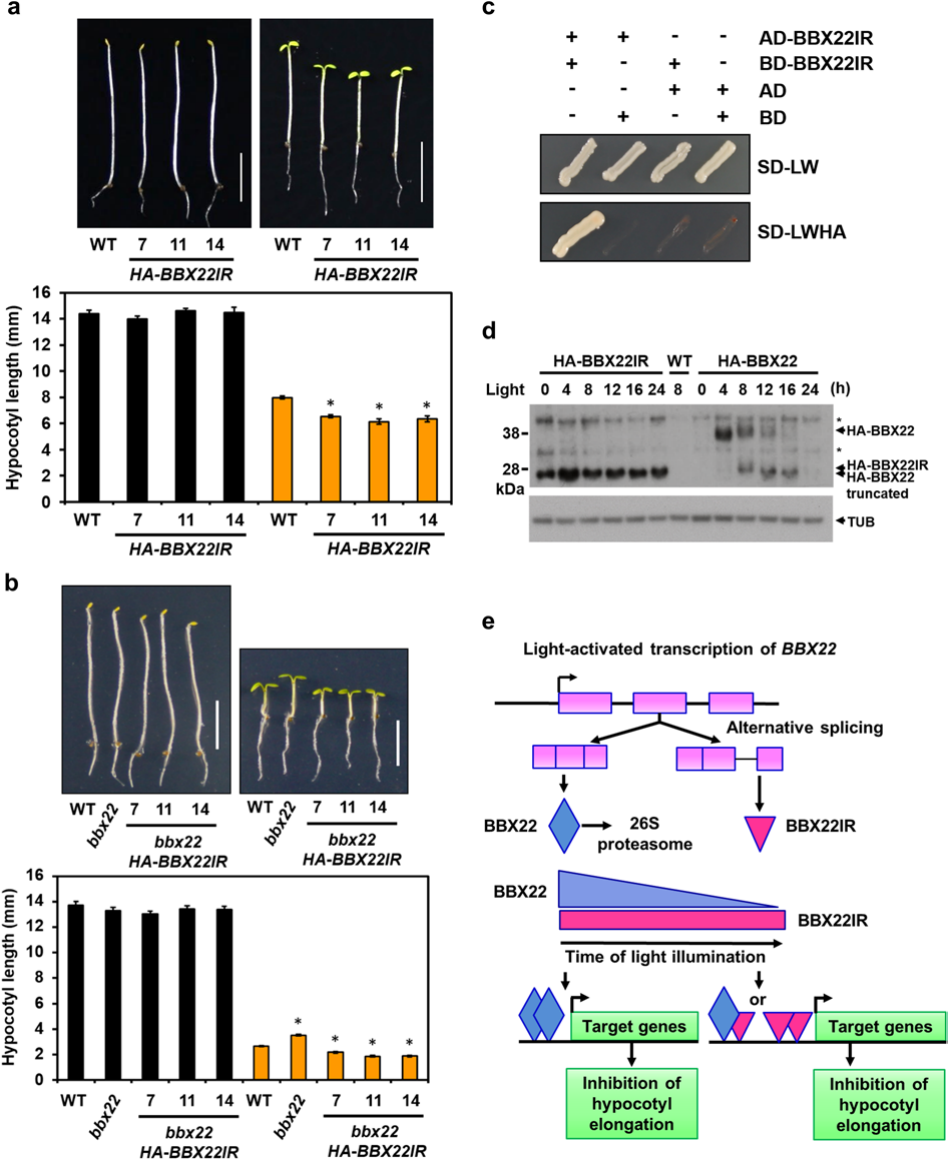
BBX22IR positively regulates photomorphogenic development. **a, b** Images and bar graphs of hypocotyl lengths for wild-type (WT) and 3 independent *35S::HA-BBX22IR* transgenic lines grown under dark (black bars) or continuous white light (10 μmole/ m^−2^ s^−1^) (orange bars) for 4 days (**a**), and WT, *bbx22* mutant and 3 independent *bbx22 HA-BBX22IR* lines under dark (black bars) or short-day condition (8-h 100 μmole/ m^−2^ s^−1^ white light and 16-h dark) (orange bars) for 4 days (**b**). The representative results from 3 biological replicates are shown. Data are mean ± SEM. * *p* < 0.001 (Student’s *t* test). Scale bar, 5 mm. **c** BBX22IR dimer formation by yeast two-hybrid assay. **d** Detection of BBX22 and BBX22IR proteins in 4-day-old etiolated Arabidopsis seedlings treated with 0, 4, 8, 12, 16, or 24 h of 75 μmole/ m^−2^ s^−1^ white light. The immunoblot was performed with anti-HA antiserum (upper panel) and anti-α tubulin antiserum (bottom panel) as loading controls. Asterisks indicate the non-specific bands. Arrowheads mark HA-BBX22, HA-BBX22 truncated, and HA-BBX22IR. **e** Model illustrates the biological function of BBX22IR in photomorphogenic development

BBX22 is a positive regulator of photomorphogenesis [11, 15]. We found no noticeable phenotypic differences for 4-day-old dark-grown wild-type (WT) and transgenic plants overexpressing BBX22IR (Fig. 7a). However, hypocotyls were significantly shorter for *HA*-*BBX22IR* lines than the WT when grown under continuous WL for 4 days (cWL), so *HA*-*BBX22IR* lines were hypersensitive to light (Fig. 7a). Thus, BBX22IR, like BBX22, may function as a positive regulator for the light-mediated inhibition of hypocotyl elongation during photomorphogenesis.

The positive role of BBX22IR in photomorphogenesis may depend on heterodimerization with BBX22, or homodimers of BBX22IR may be biologically functional. To assess the functional dependence of BBX22IR on BBX22, we introduced *BBX22IR* into the *bbx22* mutant by crossing to generate *bbx22HA*-*BBX22IR* lines. As compared with the WT, *bbx22* had long hypocotyls under short-day conditions as described previously [12, 15], whereas the *bbx22HA*-*BBX22IR* lines were light hypersensitive (Fig. 7b). Yeast two-hybrid assay also confirmed that BBX22IR expressed in yeast could form homodimers (Fig. 7c and Additional file 1: Fig. S18). Therefore, BBX22IR may function as a homodimer and positively regulate photomorphogenesis independent of the FL BBX22.

The degradation of BBX22/LZF1 in the dark and under prolonged light irradiation was reported [12]. We next investigated whether BBX22IR protein level is also subjected to selective degradation. In contrast to BBX22, BBX22IR protein level lost the time-dependent degradation pattern in the light and remained stable in the dark (Fig. 7d). The results also implied that the C-terminal part of BBX22 is required for the time-dependent and selective degradation of BBX22 protein. A simplified model in Fig. 7e depicts the role of BBX22IR in photomorphogenic development of Arabidopsis seedlings.

**Fig. 8:**
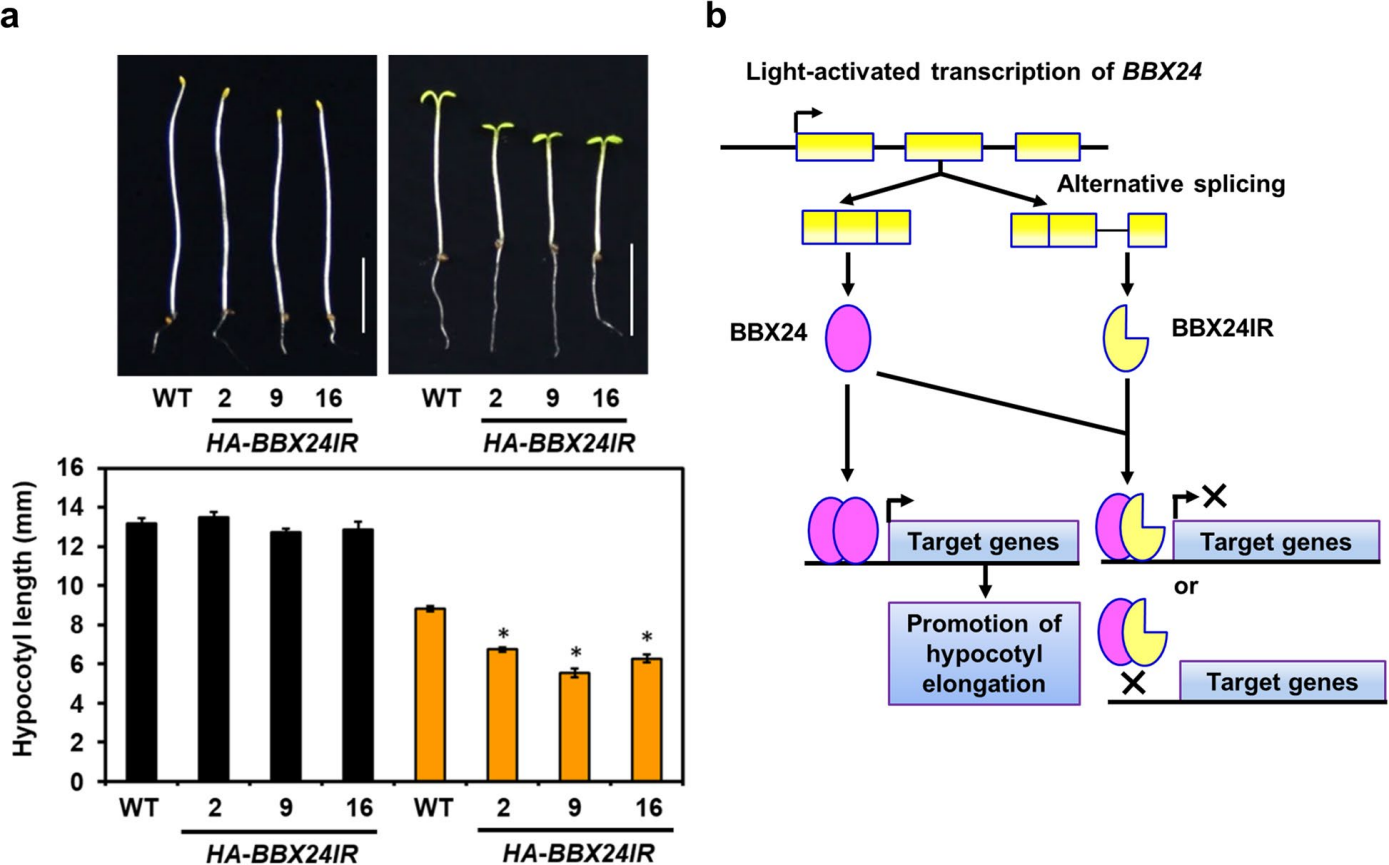
BBX24IR suppresses the function of BBX24 in photomorphogenic development. **a** Images and bar graphs of hypocotyl lengths for wild-type (WT) and 3 independent *35S::HA-BBX24IR* transgenic lines grown under dark (black bars), or continuous white light (10 μmole/m^−2^ s^−1^) (orange bars) for 4 days. The representative results from 3 biological replicates are shown. Data are mean ± SEM. * *p* < 0.001 (Student’s *t* test). Scale bar, 5 mm. **b** Model illustrates the biological function of BBX24IR in photomorphogenic development

We also investigated the biological functions of BBX24IR during photomorphogenesis. We examined the seedling de-etiolation phenotype of three *HA*-*BBX24IR* overexpression lines and the WT under dark or cWL for 4 days. Seedlings with increased *HA*-*BBX24IR* expression showed significantly shorter hypocotyls than the WT in the light, with no significant phenotype observed when seedlings were grown in the dark (Fig. 8a). This light-hypersensitive phenotype of *HA*-*BBX24IR* seedlings indicated that in contrast to the negative role of BBX24 in photomorphogenic development [66], BBX24IR functions as a positive regulator in this process. A model illustrating the dominant negative role of BBX24IR over the annotated FL BBX24 during photomorphogenic development is shown in Fig. 8b.

## Discussion

### Normalized cDNA libraries improve isoform representation by Iso-seq

Our data from normalized cDNA libraries could identify thousands of gene models missing in the non-normalized long-read datasets prepared from different Arabidopsis tissues [53, 54] (Addition file 1: Fig. S8). Normalized cDNA libraries clearly outperformed the non-normalized cDNA libraries prepared from tissues of the same developmental stages, despite the latter with a 1.7-fold Iso-seq output (Addition file 1: Fig. S19). Thus, normalization of the cDNA libraries clearly maximized the capacity of PacBio Iso-seq.

Combining cDNA normalization and size selection significantly reduced the representation of abundant genes (Fig. 1a and Addition file 1: Fig. S20). The top 10 highly expressed genes represented only 0.3% of total read counts in our normalized datasets (Additional file 3: Table S2), compared with 5–8% of the short-read RNA-seq [49] or non-normalized cDNA libraries sequenced by Iso-seq (this study). In total, our study identified 80% of the ~ 16,200 expressed genes in these developmental stages (Fig. 2c, d), an efficiency surpassing those using non-normalized libraries for PacBio Iso-seq [46, 47, 53, 67] (Addition file 1: Fig. S19 and Additional file 11:Table S10.).

Despite a clear benefit in expanding the repertoire of gene models, “quantitative” information, such as changes of expression levels, would be lost by cDNA normalization. Differential gene expression results indeed can be more readily possible with data generated from non-normalized Iso-seq libraries but discordant gene expression changes between short- and long-read sequencing platforms have been reported previously [68]. Indeed, as compared with RNA-seq, the non-normalized Iso-seq dataset prepared from tissues of the same developmental stages gave a skewed representation of abundant transcripts (Addition file 1: Fig. S20a). In addition, fold changes of gene expression in non-normalized Iso-seq data were somewhat exaggerated as compared with those derived from short-read RNA-seq (Addition file 1: Fig. S20b). The combination of short-read RNA-seq and Iso-seq with cDNA normalization likely offers the best benefits of quantitative gene expression changes and the discovery of new AS variants.

### Molecular features of transcript variants identified by full-length Iso-seq

Among different AS forms, the percentage of altA, altD, CE, ES, and IR in our results were similar to previous reports based on short-read RNA-seq [27, 69]. Notably, CIs represented ~ 10% of AS in our Iso-seq gene models, significantly higher than that described in previous studies [27, 70]. This finding may be due to intrinsic differences between sequencing methods used or the thresholds set for assembling RNA-seq reads into AS isoforms, as previously described [71, 72].

CIs are also defined as exonic introns (exitrons) [73]. A comparison of our Iso-seq results (Additional file 5: Table S4) with previously identified exitrons [73] revealed 170 genes (13.5%) shared by the two studies. However, our results revealed CIs in 1093 additional genes. The lengths for 39.7% of the exonic introns are multiples of 3, which suggests that the exclusion of these CIs will lead to a missing domain/motif in the protein encoded. For instance, a CI was detected in the *AT1G54270* transcript variant that encodes a DEAD-box RNA helicase isoform lacking the ATP-binding domain. This CI event was found conserved in Arabidopsis and humans [73]. The functional roles of these CI-containing isoforms are worthy of further investigation.

Our data revealed hundreds of transcripts spanning two separately annotated genes (Additional file 9: Table S8). We cannot rule out the possibility of transcriptional readthroughs, but a blastx search of these fusion variants indicated that many have homologs in other plant species. For example, the fusion transcript spanning *At4g32780* and *At4g32785* (Fig. 5d) is conserved and encodes a phosphoinositide-binding protein (VAN3-like protein) in the rosids clade. The fusion transcript spanning *At4g18590* and *At4g18593* (Fig. 5e) encodes a dual-specific protein phosphatase and is conserved in the Brassicaceae family. Also, the *At4g18590-At4g18593* fusion transcript is light-induced, which implies its differential roles in etiolated and de-etiolating seedlings. The underlying mechanism regulating the selective production of these transcripts requires future study.

Transcripts with variable 3′ UTRs were common in our study. Approximately 36,000 APA sites were detected in genes expressed in etiolated and de-etiolating seedlings. Consistent with previous studies [20, 54, 60, 74], the AAUAAA and UUUUUU motifs were predominant in the NUE and CE regions, respectively, in our Iso-seq results (Additional file 1: Fig. S15). In mammals, AAUAAA (~ 60%) is a major polyadenylation signal [75]. AAUAAA-like motifs were identified in the 3′ UTRs of rice transcripts [60]. In mammals, CPSF30 and WDR33/FY are responsible for recognizing and binding to the AAUAAA motif [76]. Similarly, in Arabidopsis, two protein isoforms of CPSF30 were found to regulate polyadenylation site decisions [77] and isoforms of WDR33/FY could bind to different AAUAAA-like motifs [78]. Both Araport11, a database assembled from deep short-read RNA-seq datasets [79], and our study identified AS variants of the *WDR33*/*FY* homolog in Arabidopsis.

In addition to AAUAAA (14%), our results revealed additional over-representative motifs, such as AUAAAA (12%), AUAUAU (10%), UAAUAA (8%), and AAAAAA (12%) in the NUE region, and UUUUUU with an internal U substituted by A/G/C in the CE region. The AUAUAU motif has been found enriched in transcripts from reproductive tissues in both maize and sorghum [74]. The identification of these motifs opens avenues for identifying their binding proteins and offers promise for delineating their roles in gene expression regulation.

### Comparisons of Iso-seq gene models with those in TAIR10 and Araport11

By mapping our Iso-seq results to Araport11, we identified a total of 30,418 gene models (Additional file 12: Table S11), 10,182 unannotated in Araport11. Also, 505 novel genes could be found only in our data but were unannotated in Araport11 (Additional file 13: Table S12). For the new gene models and novel genes revealed by mapping our Iso-seq data to TAIR10, 85% and 75%, respectively, were still missing in Araport11. Similar observations occurred when mapping the Nanopore long-read sequencing datasets to Araport11 and AtRTD2 databases [54]. This finding suggests that, despite efforts in increasing sequencing depth and tissue-type coverage for creating Araport11, a significant number of transcripts/genes remain to be discovered. Possible explanations include the limitations in gene assembly algorisms to distinguish between transcriptional noise and lowly expressed but bona fide transcripts and the use of short-read RNA-seq data without strand-specific information, etc.

In addition to alternative splicing, TSSs and PASs contribute to the complexity of transcript variants. TSSs and PASs identified by our Iso-seq gene models were cross validated by their presence in several orthogonal datasets (Addition file 1: Fig. S7 and Fig. S13). When we compared all gene models derived from our study with those in TAIR10 or Araport11, our gene models were slightly shorter than those in Araport11 that was based on short-read RNA-seq assembly (Addition file 1: Fig. S21 and Additional file 12: Table S11), similar to a few previous studies [48, 51, 54, 80].

### Biological evidence of BBX22IR and BBX24IR in de-etiolating Arabidopsis

The thousands of AS variants we identified may engage in translation by their association with the polysome fractions [62, 69, 81] or protection by ribosomes. Light also imposes expression regulation of AS variants identified in this study (Fig. 6a). Inspired by previous studies showing that AS transcript variants of photomorphogenic regulators can function to modulate photomorphogenic fitness [28–31, 33], we investigated and confirmed that the BBX22IR and BBX24IR protein isoforms can interact with their annotated FL proteins, BBX22 and BBX24, respectively (Fig. 6b), to regulate the photomorphogenic development (Fig. 7a, b and Fig. 8a).

The expression of BBX22 and BBX24 is controlled at both the transcriptional and post-translational levels [11, 12, 15, 82]. BBX22IR and BBX24IR both encode C-terminal truncated protein isoforms, lacking the VP-like motif. The VP motif has been identified as a COP1-interacting motif that regulates protein stability of BBX24 [82]. Indeed, BBX22IR has a long half-life during photomorphogenic development (Fig. 7d). A previous report showed that BBX23 and BBX22 were functionally redundant in mediating photomorphogenesis [83]. BBX23 has 2 B-box domains in the N-terminus but lacks VP motif and flanking sequences in the C-terminus, resembling BBX22IR. We have shown that, similar to BBX23, BBX22IR functions as a positive regulator (Fig. 7a, b). Thus, BBX22IR and BBX23 have both sequence and functional resemblance.

The C-terminal domain of BBX24 plays a decisive role in promoting hypocotyl growth [84]. BBX24IR does not likely possess this function because it encodes a C-terminal truncated protein. Consistent with this notion, our study showed that BBX24IR has a dominant negative role over BBX24 by sequestering BBX24 via heterodimer formation (Fig. 6b and Fig. 8a). BBX24 can heterodimerize with HY5 or HYH to repress the activation of *BBX22* expression [18, 85]. By sequestering BBX24, the overexpression of BBX24IR may lead to release of HY5 and HYH for activating *BBX22*, thus resulting in the light-hypersensitive phenotype observed.

Together with previous studies, we found that the expression of both positive and negative regulators of photomorphogenesis, BBX22 and BBX24, respectively, was sophisticatedly regulated at the transcriptional, post-transcriptional, and post-translational level. The various stability and functionalities of BBX isoforms can allow for a highly versatile regulation of photomorphogenic development in response to the everchanging light environment.

## Conclusions

The combination of normalized cDNA libraries and PacBio Iso-seq successfully potentiates the identification of genes/transcript variants in two key developmental stages in plants, skotomorphogenesis, and early photomorphogenesis. More than one third of the gene models detected in our study are new gene models or were previously unannotated in the Arabidopsis genome. The full-length nature of Iso-seq allowed for accurate annotation of AS, alt-TSS/alt-TES, or APA for each transcript sequenced, thus enabling an unequivocal determination of gene structure and functional analyses of regulatory elements or protein coding. Isoform-specific transcriptomes also open a new window to identify new players regulating photomorphogenic development. Indeed, our results revealed regulatory functions of 2 selected IR forms of light-regulated BBX family members, BBX22IR and BBX24IR, in photomorphogenic development. Our approach provides a blueprint for future studies aimed at elucidating the functional consequences of AS isoforms in developmental processes, responses to environmental stimuli, and functional diversity of evolutionarily closely related species.

## Methods

### Plant material and growth conditions

*Arabidopsis thaliana* ecotype Columbia-0 (Col-0) was used in this experiment. Seeds were surface-sterilized, evenly distributed on half-strength MS medium plates (0.8% agar, pH 5.7), and kept at 4 °C in darkness for 4 days for stratification. After exposure to 2 h light for synchronizing germination, plates were covered with multi-layered aluminum foil and put in a dark box placed in a growth chamber at 22 °C for 4 days for growing etiolated seedlings. Etiolated seedlings were illuminated with 100 μmole m^−2^s^−1^ white light for 4 h (L4h) or remained in the dark for 4 h (D4h).

### PacBio Iso-seq library preparation and sequencing

Total RNA was extracted by using Plant RNA reagent (Invitrogen) and subjected to polyA RNA purification with Dynabeads (Thermo). The purity and quantity of RNA samples were analyzed with RNA 6000 Nano LabChip by a 2100 Agilent Bioanalyzer (Agilent Technologies).

Double-stranded cDNA libraries were constructed from 1.5 μg polyA RNA by using SMARTScribe reverse transcriptase (Clontech) with 3′ SMART CDS Primer II A and 5′ SMART II A oligonucleotide following SMART PCR cDNA synthesis procedures (Clontech). A total of 5 cycles of PCR amplification were performed with 5′ PCR Primer II A by using KAPA HiFi DNA polymerase (Kapa Biosystems). PCR products were purified by using AMPure PB Beads and quantified by using Agilent DNA 1000 Chip (Agilent Technologies).

Normalization of D4h and L4h cDNA libraries involved using the Trimmer-2 cDNA normalization kit (Evrogen). Briefly, 400 ng double-stranded cDNAs were denatured at 98 °C for 2 min and hybridized at 67 °C for 6 h. The hybridized cDNAs were treated with 0, 0.75, 1, or 1.25 unit duplex-specific nuclease (DSN) at 68 °C for 25 min and the DSN was inactivated with the DSN stop buffer. Efficiency of the normalization was checked by real-time RT-PCR with primer pairs for high- and low-expressed genes, *UBQ10* and *DNAJ*, respectively (Additional file 1: Fig. S2). For amplification of normalized cDNA, 1 unit DSN-treated cDNA libraries were subjected to 9 cycles of PCR amplification with 5′ PCR Primer II A by using KAPA HiFi DNA polymerase (Kapa Biosystems).

For size fractionation, 3 μg normalized cDNA was separated on a 0.75% Agarose Marker 75 cassette (Sage Science) by using the SageELF (Sage Science). cDNAs at 1–2, 2–3, and 3–4 kb were further amplified with 8, 9, and 12 PCR cycles with KAPA HiFi DNA polymerase, respectively. SMARTbell libraries of 1–2 and 2–4 kb cDNAs were prepared separately by pooling fractions 9 and 10 at a ratio of 7:3 (1–2 kb, 500 ng for library I) and fractions 6, 7, and 8 at a ratio of 1:3:6 (2–4 kb, 1 μg for library II) (Additional file 1: Fig. S3), according to size distribution of expressed genes.

SMRTbell libraries, two each for libraries I and II, were prepared by following the protocols of repairing the DNA damage, blunt end-ligation with SMRTbell hairpin adapters, and removal of failed ligation products by using DNA Template Prep Kit 2.0 (Pacific Biosciences). After purification, the quality and quantity of the four libraries were assessed by the Agilent 2100 Bioanalyzer system (Agilent Technologies) with DNA 12000 chip and Qubit dsDNA BR assay. SMRTbell libraries were prepared for sequencing by using the DNA polymerase binding kit P6 v2 primers (Pacific Biosciences) and the Magbead Binding Kit (Pacific Biosciences). The sequencing was performed on a PacBio RS II sequencer platform with eight v3 SMRTcells (Pacific Biosciences). Each of the four libraries was sequenced on two cells by C4 sequencing reagent (Pacific Biosciences) with 240-min signal collections.

For non-normalized libraries, RNAs were isolated from 4-day-old etiolated seedlings (D4h) or 4-day-old etiolated seedling treated with 4-h light (L4h). PacBio libraries were prepared according to the Pacific Biosciences’s protocol using the SMARTer PCR cDNA Synthesis kit (Clontech) and amplification by the KAPA DNA polymerase. PacBio SMRT cDNA libraries were prepared with the SMRTbell Template Prep Kit 1.0 (Pacific Biosciences) and sequenced on the PacBio Sequel I with Sequel DNA polymerase and binding kit and sequencing chemistry version 2.1 for 20 h on individual SMRT cell.

### Iso-seq data analyses for the identification of novel transcripts and AS variants

The PacBio raw reads were initially assembled by using Pacific Bioscience SMRT analysis software v2.3.0. Read of Insert (ROI) was generated by using the default number of polymerase full passes from each zero-mode waveguide (ZMW). Reads that did not contain the 5′ or 3′ adapters, a polyA tail or had read length < 300 nt were removed by using an Iso-Seq classify tool [44]. Full-length (FL) non-chimeric reads were subjected to clustering by using the Iterative Clustering for Error Correction (ICE) algorithm to facilitate sequence accuracy. The non-FL non-chimeric reads were used to polish the FL consensus sequences to produce high-quality (HQ) consensus sequences by using the Quiver algorithm [86] packaged in SMRT analysis software (version 2.3.0). Only HQ FL consensus reads with ≥ 99% post-correction accuracies were further analyzed.

All HQ reads were mapped to the TAIR10 genome by using three approaches to determine the best matching results: (1) mapped to the TAIR10 transcriptome by using Bowtie2 [87], (2) mapped to the TAIR10 genome by using GMAP, and (3) mapped to the TAIR10 genome by using BLAT. During the mapping processes, HQ FL reads were corrected by restoring mismatching bases and ignoring minor indels < 10 nt. Reads were considered from novel genes if they did (1) not overlap with any annotated TAIR10 genes or (2) had < 50% overlapping and did not match the splicing junctions of the annotated genes in the opposite strand.

Orthogonal long-read datasets, Pacbio Iso-seq (BioSample Name: SAMN13510395, file name: SRX729046) [53] and Nanopore direct RNA sequencing (European Nucleotide Archive (ENA) Project: PRJEB32782, file name: ERX3766449 and ERX3766453) [54] were downloaded from NCBI and designated as Pacbio Iso-seq_2020 and Nanopore_2020, respectively, for comparisons. The downloaded data were processed following the same procedures described above to generate gene models except introns ≤ 20 nt were filtered for Nanopore_2020 to remove gene models with small indels.

Reads that varied only in the 5′ or 3′ extensions were not considered splicing isoforms. Reads of intron retention (IR) were those with introns fully subsumed by the neighbor exons. Overlapping exons that differed at their splice junctions were considered alternative splicing donor or acceptor events (AltD or AltA), respectively. Isoforms lacking annotated exons were considered as exon skipping (ES). The splicing events within annotated exons or intron were labeled as cryptic introns (CIs) or cryptic exons (CEs). Transcripts with start or end exons different from the representative models were defined as alternative start exons or alternative end exons.

### Sequence analyses of novel transcripts

The search for novel transcripts involved examining (1) the sequence similarities to nucleotide sequences in the databases and (2) their coding potential. Sequence corrected reads were used to search the NCBI refseq RNA database and the TAIR10 annotated gene and miRNA stem-loop sequences in miRbase [88]. Novel transcripts had to have homologs in other species with E-value < 1e^−50^ by using BLASTN. Any reads with at least 50 nt complementary pairings to the annotated genes in TAIR10 were defined as antisense transcripts [89]. Those novel transcripts were considered as “coding” with coding potential score > 0 or as “non-coding” with score ≤ 0 by using Coding Potential Calculator 2 [55]. Those determined as “coding” and with the longest deduced peptides > 30 a.a [90]. were used to query the NCBI database for plant species excluding Arabidopsis by using BLASTP.

### Assembly of gene models based on short-read RNA-seq

RNA-seq reads [49] were preprocessed using Trimmomatic [91] to remove adapter sequences. TopHat2 [92] was used for read mapping guided by TAIR10 and Iso-seq gene models. Alignment results were processed using Cufflinks 2.2.0 (http://cole-trapnell-lab.github.io/cufflinks/manual/) [93] for isoform identifications guided by TAIR10 and Iso-seq models.

### Alternative polyadenylation site analyses

Alternative polyadenylation site detection involved aligning the 3′ ends by using the following probabilistic approach. For a given gene model, aligned sequences were centered at the 3′ ends of its alignments to form normal distributions with standard deviations of 10 bp. Peaks were then identified from the summation curves of these normal distributions as the predicted polyadenylation sites. When the value of the other summation curve at the same position was ≤ 0.001, it was considered an alternative polyadenylation site.

For flanking sequence analyses, the 50-nt upstream and downstream regions were extracted from the HQ FL corrected reads and the reference genome, respectively. Over-representative motifs were revealed by analyzing a 6-nt window in a − 50 to + 10 region surrounding the poly-A sites by SignalSleuth2 [94]. The 6-mer patterns with KS test (*p* < 10^−5^) against a first-order Markov chain model were selected, and those with the top 20% total frequencies were clustered by using the MACCU toolkit [95]. To reveal over-representing motifs, the sequences at the two enriched regions were extracted for motif discovery by using MEME [96] with an ACGU-equal background. The best-resulting motif was used to scan against its corresponding region and those with *p* < 0.01 were assembled into hexamer patterns shown in Additional file 1: Fig. S 15 by using weblogo3 [97].

### Computation of gene expression correlations

Correlation values between two neighboring Arabidopsis genes were computed based on read count data downloaded from the DEE2 database [98]. The corresponding metadata file was downloaded for data selection with high-quality and unambiguous BioSamples. The 5557 BioSamples for ecotype Col-0 were collected, and read counts were normalized using the TMM method [99]. Pearson correlation coefficients between pairs of consecutive-and-nonfused genes and pairs of predicted fusion genes were computed based on the normalized and log transformed read counts.

### RT-PCR

Total RNA was isolated from D4h and L4h by using the Plant RNA reagent (Invitrogen) and then treated with DNase I (Promega). RT-PCR reactions involved using Superscript IV (Invitrogen) and GenTaq DNA Polymerase (GenMark) with the gene-specific primers listed in Additional file 14: Table S13. For novel transcripts, six coding transcripts with the highest coding potential scores and with coding sequence > 300 nt and six non-coding transcripts with the lowest coding potential scores and transcript length > 800 nt underwent validation by RT-PCR. For AS or alternative transcription start/end transcripts, three representative genes, *At3g09600*, *At4g32780-At4g32785*, and *At4g18590-At4g18593*, were selected for validation. PCR products were confirmed by Sanger sequencing.

### Quantitation of AS variants

The annotated and IR variants of the 5 light-regulated transcription factors *BBX22*, *BBX24*, *PIF3*, *PIF4*, and *HY5* were amplified and cloned. The intron retained and spliced fragments of known quantities were used to established standard curves for quantitative RT-PCR with gene-specific primers within or spanning the retained intron (Additional file 14: Table S13) by using the Power SYBR green PCR master mix (Applied Biosystem). *UBQ10* was used as an internal control for normalization. Quantification results were replicated three times by measuring the cycle threshold (CT) value and compared with established standard curves. The 100% was defined as the total expression level in L4h samples. Three independent biological replicates were performed.

### Yeast two-hybrid and protein expression analyses

The coding sequences of the FL representative transcripts for *BBX22*, *BBX24*, *PIF3*, *PIF4*, and *HY5* and their intron retained transcripts *BBX22IR*, *BBX24IR*, *PIF3IR*, *PIF4IR*, and *HY5IR* (Fig. 6a) were cloned into the pGAD-T7 (Clontech) and pGBK-T7 vectors (Clontech), respectively. For the yeast two-hybrid system, the *S*. *cerevisiae* strain AH109 (Clontech) was transformed with the pGAD-T7 vector or pGAD-T7 vector carrying FL cDNA and the pGBK-T7 vector or pGBK-T7 vector carrying its IR isoforms. Transformants were selected on drop-out medium lacking leucine and tryptophan leucine (DO-LW) (Clontech) as a control and DO-LWHA (–Leu –Trp –His –Adenine) medium for selecting interactions at 28 °C for 2 days.

Yeast total proteins were extracted from 5 ml cultures with yeast lysis buffer (40 mM Tris-HCl, pH 7.5, 8 M Urea, 5% SDS, 1 mM DTT, 1 mM EDTA, 4 mM PMSF complete EDTA-free protease inhibitor cocktail (Roche) by a beat beater (Biospec)). Approximately 30 to 50 μg total protein was separated by 4–12% NuPAGE Bis-Tris gel (Invitrogen) and transferred to PVDF membranes (Amersham, Hybond). Western blot analyses were performed with the SNAP i.d. 2.0 system according to the manufacturer’s instructions by using anti-Gal4-BD antiserum (Abcam, PN: ab135397) and anti-Gal4-AD antiserum (Clontech, PN:630402) and horseradish peroxidase-conjugated anti-mouse antiserum (Santa Cruz Biotechnology). An ultra-sensitive enhanced chemiluminescent (ECL) horseradish peroxidase substrate (Thermo, PN: 34095) and Amersham hyperfilm ECL (GE Healthcare) were used for signal detection.

### Construction of transgenic plants

The coding sequences of *BBX22IR* and *BBX24IR* were amplified by RT-PCR by using Phusion DNA polymerase (NEB) and gene-specific primers (Additional file 14: Table S13) and cloned into the pCAMBIA 1390 vector to create in-frame fusion to the epitope HA under control of the 35S promoter and called p1390 *35S*::*HA*-*BBX22IR* and p1390 *35S*::*HA*-*BBX24IR*. *Agrobacterium GV3101* harboring the constructs were used for generating *HA*-*BBX22IR* and *HA*-*BBX24IR* transgenic plants by floral dipping [100]. HA-BBX22IR independent lines were crossed to the *bbx22-1/lzf1-1* mutant line [11] to assess the biological function of BBX22IR in the *bbx22* mutant background. The expression of BBX22IR or BBX24IR was detected by western blot analyses of isolated proteins as described [12] and with anti-HA antiserum (Sigma) and horseradish peroxidase-conjugated anti-mouse antiserum (Santa Cruz Biotechnology)

### Hypocotyl length measurement

T3 or F3 homozygous seedlings were grown in the dark or illuminated with 10 μmole m^−2^ s^−1^ continuous white light (cWL) for 4 days or 100 μmole/ m^−2^ s^−1^ WL under short-day conditions for 4 days. The hypocotyl length was measured by using ImageJ v1.47. Student’s *t* test was used to evaluate differences in hypocotyl lengths between wild-type and transgenic seedlings. Three biological replicates were performed.

## Supplementary Information

**Additional file 1: Figure S1**. Schematic illustration of experimental design. **Figure S2**. cDNAs were effectively normalized. **Figure S3**. Size fractionations of normalized D4h and L4h cDNA libraries. **Figure S4**. A flowchart of data analysis pipeline. **Figure S5**. Rates of error types in this study. **Figure S6**. Iso-seq reads with at least 80% coverage aligned well with TAIR10-annotated transcript start sites (TSSs) and transcript end sites (TESs). **Figure S7**. Pair-wise alignments for 5’ ends of orthologous transcripts in Iso-seq (this study), TAIR10, and nanoPARE. **Figure S8**. Comparison of gene models identified in three long-read sequencing datasets. **Figure S9**. Comparison of gene models identified by Iso-seq and those assembled by RNA-seq data. **Figure S10**. Analyses of canonical GU-AG splicing sites in unique AS events identified in three long-read sequencing datasets. **Figure S11**. Nineteen alternative splicing isoforms of *At3g02600.* **Figure S12**. Co-expression analysis of fused or non-fused transcripts. **Figure S13**. Authenticity of poly(A) sites identified from Iso-seq in this study. **Figure S14**. Analysis of alternative polyadenylation sites (APAs) in the Iso-seq datasets. **Figure S15**. The enriched near upstream elements (NUEs) and cleavage elements (CEs). **Figure S16**. Expression analyses of AD- and BD-fusion proteins in yeast. **Figure S17**. Expression analyses of HA-BBX22IR and HA-BBX24IR in independent transgenic Arabidopsis plants. **Figure S18**. Expression analyses of AD-BBX22IR and BD-BBX22IR in yeast. **Figure S19**. A direct comparison of gene models identified from Iso-seq libraries from cDNA-normalized and non-normalized Iso-seq datasets in gene and gene model identifications. **Figure S20**. A direct comparison of gene expression and differential gene expression in RNA-seq, normalized Iso-seq and non-normalized Iso-seq data. **Figure S21**. Alignments of 5’ and 3’ ends of gene models from Iso-seq datasets with those in TAIR10 and Araport11.

**Additional file 2: Table S1.** Statistics of the Iso-seq data in this study.

**Additional file 3: Table S2.** Expression levels of the top 10 most abundant transcripts and their representation in D4h and L4h Iso-seq datasets.

**Additional file 4: Table S3.** A summary of Iso-seq gene models exceeding annotated transcript length.

**Additional file 5: Table S4.** A summary of full-length gene models identified in this study.

**Additional file 6: Table S5.** A list of 671 TAIR10 unannotated genes.

**Additional file 7: Table S6.** A comparison of gene models identified in three long-read datasets.

**Additional file 8: Table S7.** A comparison of gene models based on Iso-seq and RNA-seq.

**Additional file 9: Table S8.** A summary of fusion transcripts identified in this study.

**Additional file 10: Table S9.** Read counts of ribosome protected fragments for introns of transcription factor genes.

**Additional file 11: Table S10.** A comparison of Iso-seq gene models from libraries with or without cDNA normalization.

**Additional file 12: Table S11.** A summary of Iso-seq-identified full-length gene models based on Araport11.

**Additional file 13: Table S12.** Novel genes identified in this study and their Araport11 annotations.

**Additional file 14: Table S13.** Primers used in this study.

**Additional file 15.** Uncropped blots for Figures 4, 5 and 7.

**Additional file 16.** Review history.

## Data Availability

The raw data generated in this study have been deposited at the NCBI’s Sequence Read Archive (SRA) under the BioProject number: PRJNA726863 and SRR accessions: SRR14400342 to SRR14400349 for normalized D4h and L4h datasets, and SRR15010481/SRR15010480 for non-normalized D4h/L4h datasets [101], respectively. The published data downloaded and used in this study include the following: RNA-seq datasets for seedling under the same developmental stages: accession number GSE43703 [49], nanoPARE datasets: accession number GSE112869 [51], Pacbio Iso-seq datasets: accession number GSE141641 [53], Nanopore direct RNA sequencing datasets: accession number PRJEB32782 [54] and direct RNA sequencing (DRS) datasets: accession number PRJEB2761 [57]. The file of Arabidopsis high-confidence poly(A) site clusters is available on PlantAPAdb [58] at http://www.bmibig.cn/plantAPAdb/Bulkdownload.php.
