# Supplementary material for: An improved repertoire of splicing variants and their potential roles in Arabidopsis photomorphogenic development
Source: Genome Biol. 2022 Feb 9;23:50. doi: 10.1186/s13059-022-02620-2 (PMC8827149; doi:10.1186/s13059-022-02620-2)
Supplement: Supplementary file 1 — Additional file 1: Figure S1. Schematic illustration of experimental design. Figure S2. cDNAs were effectively normalized. Figure S3. Size fractionations of normalized D4h and L4h cDNA libraries. Figure S4. A flowchart of data analysis pipeline. Figure S5. Rates of error types in this study. Figure S6. Iso-seq reads with at least 80% coverage aligned well with TAIR10-annotated transcript start sites (TSSs) and transcript end sites (TESs). Figure S7. Pair-wise alignments for 5’ ends of orthologous transcripts in Iso-seq (this study), TAIR10, and nanoPARE. Figure S8. Comparison of gene models identified in three long-read sequencing datasets. Figure S9. Comparison of gene models identified by Iso-seq and those assembled by RNA-seq data. Figure S10. Analyses of canonical GU-AG splicing sites in unique AS events identified in three long-read sequencing datasets. Figure S11. Nineteen alternative splicing isoforms of At3g02600. Figure S12. Co-expression analysis of fused or non-fused transcripts. Figure S13. Authenticity of poly(A) sites identified from Iso-seq in this study. Figure S14. Analysis of alternative polyadenylation sites (APAs) in the Iso-seq datasets. Figure S15. The enriched near upstream elements (NUEs) and cleavage elements (CEs). Figure S16. Expression analyses of AD- and BD-fusion proteins in yeast. Figure S17. Expression analyses of HA-BBX22IR and HA-BBX24IR in independent transgenic Arabidopsis plants. Figure S18. Expression analyses of AD-BBX22IR and BD-BBX22IR in yeast. Figure S19. A direct comparison of gene models identified from Iso-seq libraries from cDNA-normalized and non-normalized Iso-seq datasets in gene and gene model identifications. Figure S20. A direct comparison of gene expression and differential gene expression in RNA-seq, normalized Iso-seq and non-normalized Iso-seq data. Figure S21. Alignments of 5’ and 3’ ends of gene models from Iso-seq datasets with those in TAIR10 and Araport11. [file 13059_2022_2620_MOESM1_ESM.pdf]

An improved repertoire of splicing variants and their potential roles in Arabidopsis photomorphogenic development

Chun-Kai Huang, Wen-Dar Lin and Shu-Hsing Wu

Additional File 1:

Figure S1. Schematic illustration of experimental design..................... **3**
Figure S2. cDNAs were effectively normalized.................................... **4**
Figure S3. Size fractionations of normalized D4h and L4h cDNA libraries..…………………………………………………………………... **5**
Figure S4. A flowchart of data analysis pipeline.…............................. **6**
Figure S5. Rates of error types in this study....................................... **7**
Figure S6. Iso-seq reads with at least 80% coverage aligned well with TAIR10-annotated transcript start sites (TSSs) and transcript end sites (TESs)………………………………………………………………… **8**
Figure S7. Pair-wise alignments for 5' ends of orthologous transcripts in Iso-seq (this study), TAIR10, and nanoPARE …………..…….…….…….. **9**
Figure S8. Comparison of gene models identified in three long-read sequencing datasets.…..………… **10**
Figure S9. Comparison of gene models identified by Iso-seq and those assembled by RNA-seq data.………...**11**
Figure S10. Analyses of canonical GU-AG splicing sites in unique AS events identified in three long-read sequencing datasets.................... **12**
Figure S11. Nineteen alternative splicing isoforms of *At3g02600*........ **13**
Figure S12. Co-expression analysis of fused or non-fused transcripts.**14**
Figure S13. Authenticity of poly(A) sites identified from Iso-seq in this study...**15**
Figure S14. Analysis of alternative polyadenylation sites (APAs) in the Iso-seq datasets..**16**
Figure S15. The enriched near upstream elements (NUEs) and cleavage elements (CEs)...**17**,**18**

Figure S16. Expression analyses of AD- and BD-fusion proteins in yeast..**19**
Figure S17. Expression analyses of HA-BBX22IR and HA-BBX24IR in independent transgenic Arabidopsis plants.......................................**20**
Figure S18. Expression analyses of AD-BBX22IR and BD-BBX22IR in yeast.. **21**
Figure S19. A direct comparison of gene models identified from Iso-seq libraries from cDNA-normalized and non-normalized Iso-seq datasets in gene and gene model identifications……………..**22**
Figure S20. A direct comparison of gene expression and differential gene expression in RNA-seq, normalized Iso-seq and non-normalized Iso-seq data...**23**
Figure S21. Alignments of 5' and 3' ends of gene models from Iso-seq datasets with those in TAIR10 and Araport11.................................... **24**

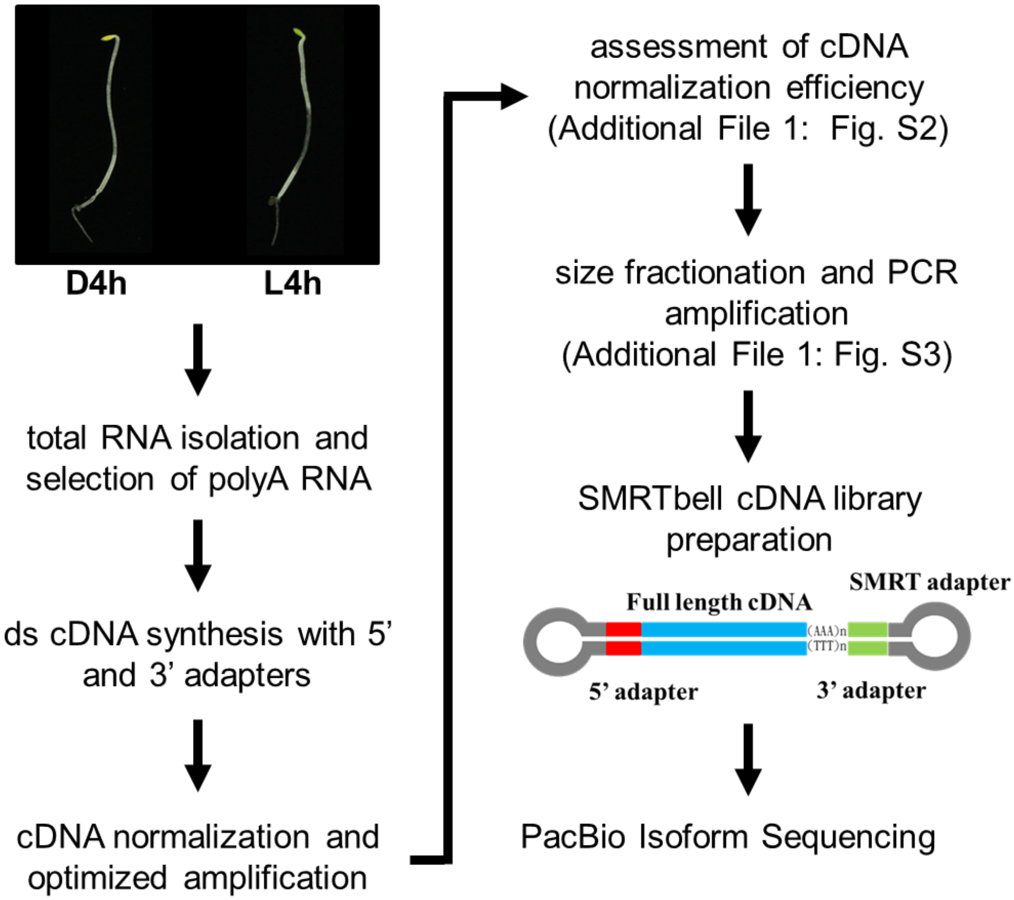

Figure S1. Schematic illustration of experimental design.
Detailed descriptions of experiments are in Methods.

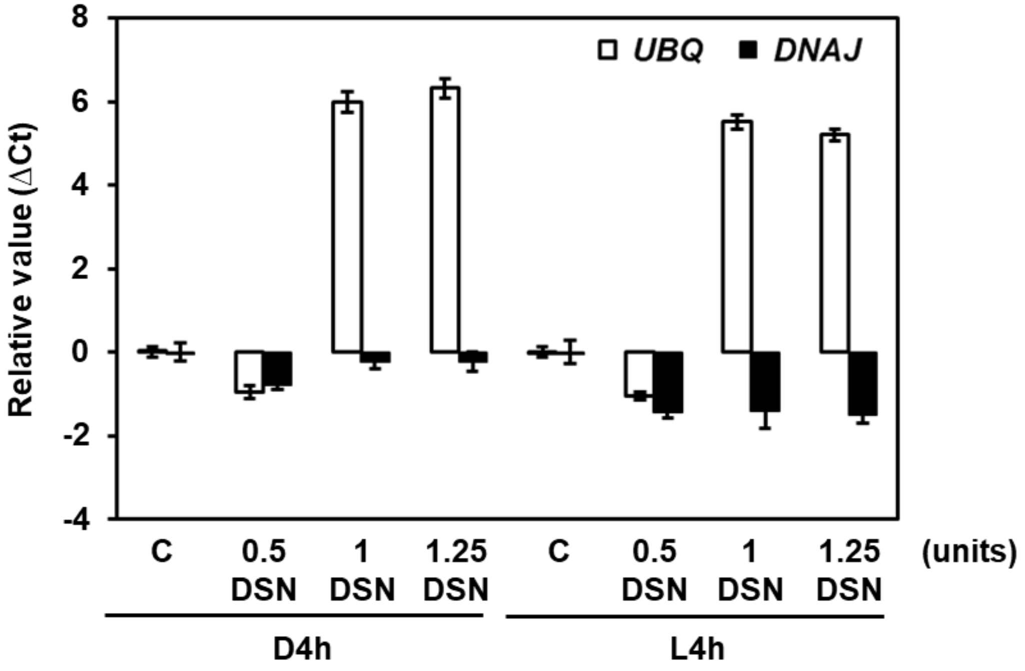

Figure S2. cDNAs were effectively normalized.
Quantitative RT-PCR analyses of the normalized D4h and L4h cDNA treated with various units of dsDNA-specific nuclease (DSN) treated samples. *UBQ10* (white bar) and *DNAJ* (black bar) represent a high- and low-abundance gene, respectively. The relative value (ΔCt) of each gene was calculated between control (C) and DSN-treated samples. $\Delta Ct > 5$ for the abundant transcript in a given cDNA library was considered successfully normalized. Data are mean ±SD from three technical repeats.

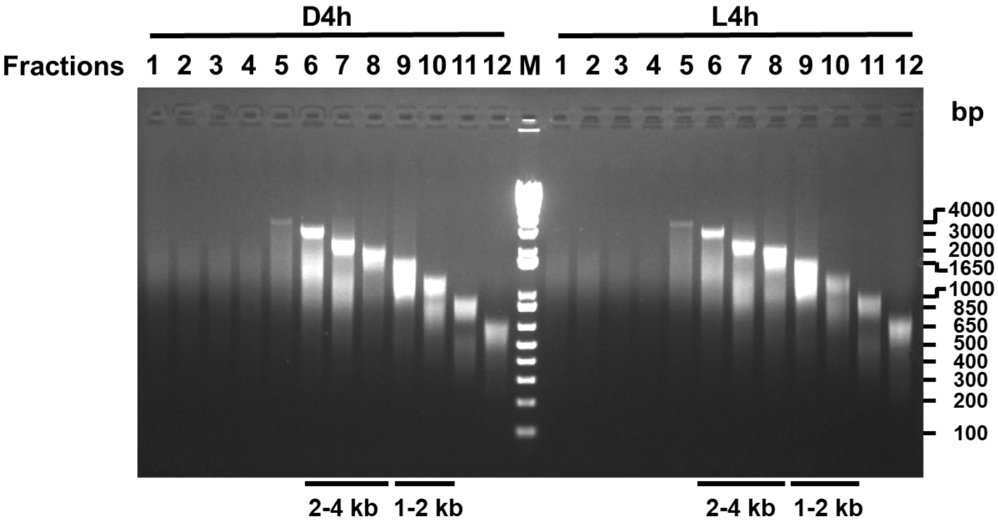

Figure S3. Size fractionations of normalized D4h and L4h cDNA libraries.
The gel image shows 12 size fractions of normalized D4h and L4h cDNAs on 0.75% Agarose Maker 75 cassette with SageELF. Fractions 9 and 10 were used for Iso-seq cDNA libraries of 1-2 kb in size and fractions 6-8 for 2-4 kb.

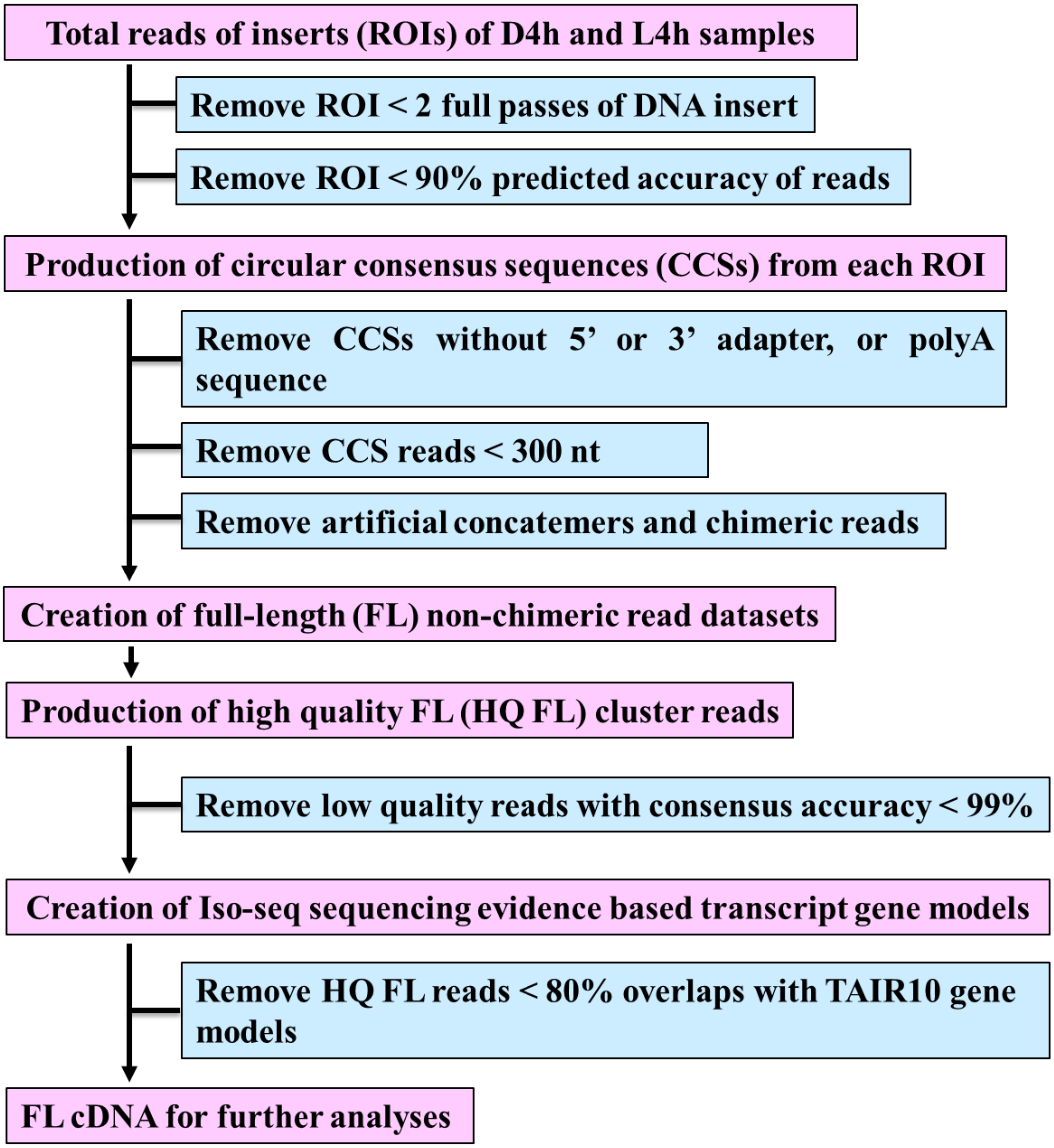

Figure S4. A flowchart of data analysis pipeline.
The pipeline outlines the steps and filtering criteria used for the identification of high-quality full-length (HQ FL) transcripts and the creation of transcript gene models in this study.

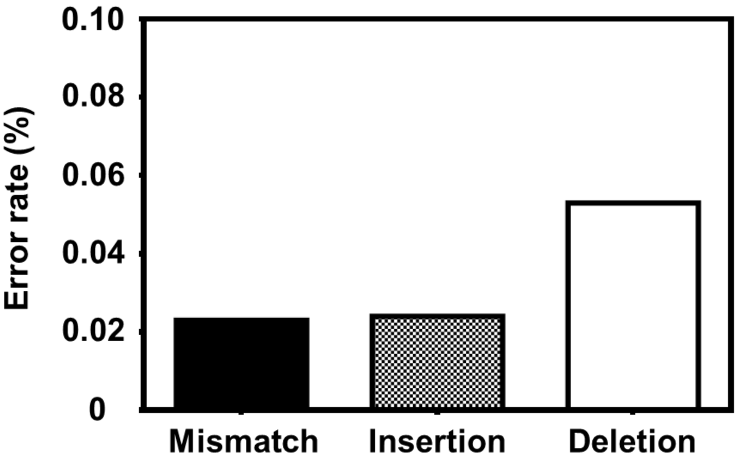

Figure S5. Rates of error types in this study.
A total of 162,049 HQ FL reads were mapped to the TAIR10 reference genome by GMAP. Error rates for mismatch, insertion or deletion were calculated and plotted.

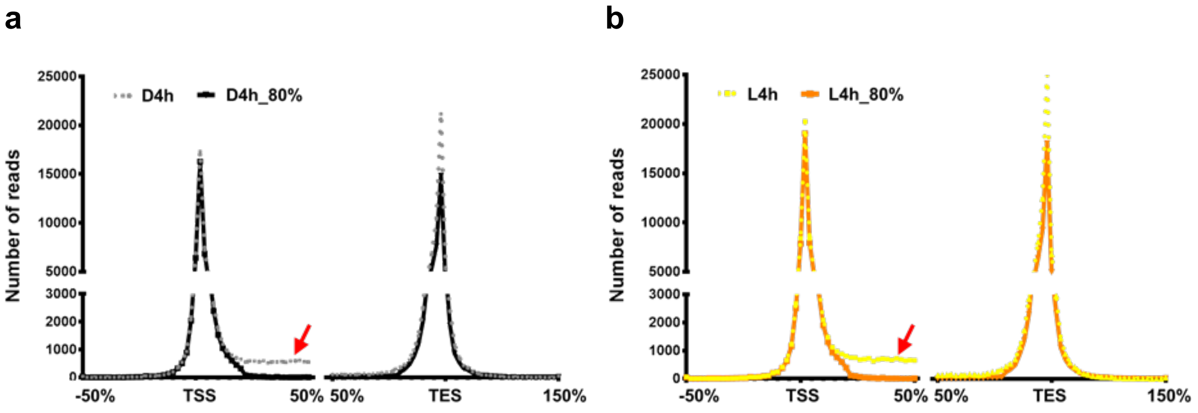

Figure S6. Iso-seq reads with at least 80% coverage aligned well with TAIR10-annotated transcript start sites (TSSs) and transcript end sites (TESs).
a, b The 5’ and 3’ nucleotides for the total Iso-seq HQ reads (D4h: grey, L4h: yellow) or Iso-seq HQ reads with ≥ 80% transcript coverage (D4h: black, L4h: orange) were plotted to coordinates related to the annotated TSS and TES. The length of each annotated transcript was set to 100%. Red arrowhead indicates reads with potential 5’-end truncations in D4h (**a**) and L4h (**b**) Iso-seq datasets.

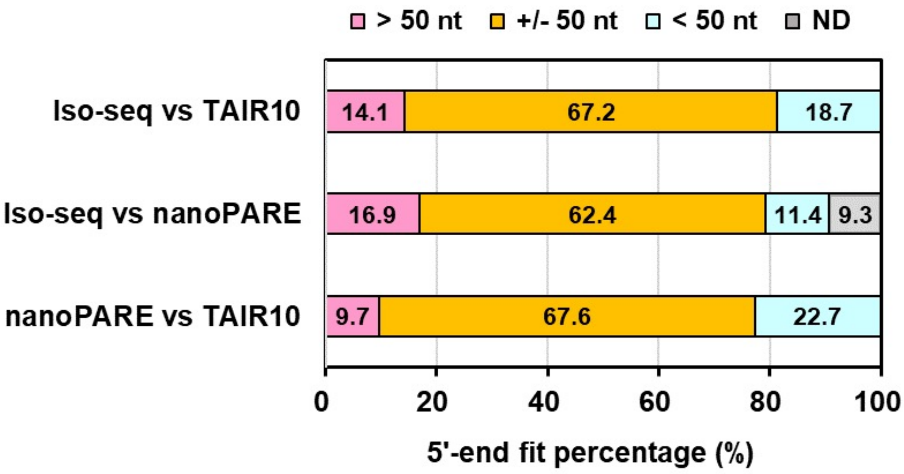

Figure S7. Pair-wise alignments for 5' ends of orthologous transcripts in Iso-seq (this study), TAIR10, and nanoPARE.

Percentage showing the extent of 5' end overlap between Iso-seq HQ FL reads and the reference transcripts in TAIR10 or nanoPARE datasets (51). Results were calculated based on a 50-nt window used previously. ND indicates reads detected in Iso-seq but undetected in nanoPARE datasets. This possibly is due to the differential gene coverage in tissues used to generate the cDNA libraries: young seedlings for Iso-seq and floral buds for nanoPARE (51).

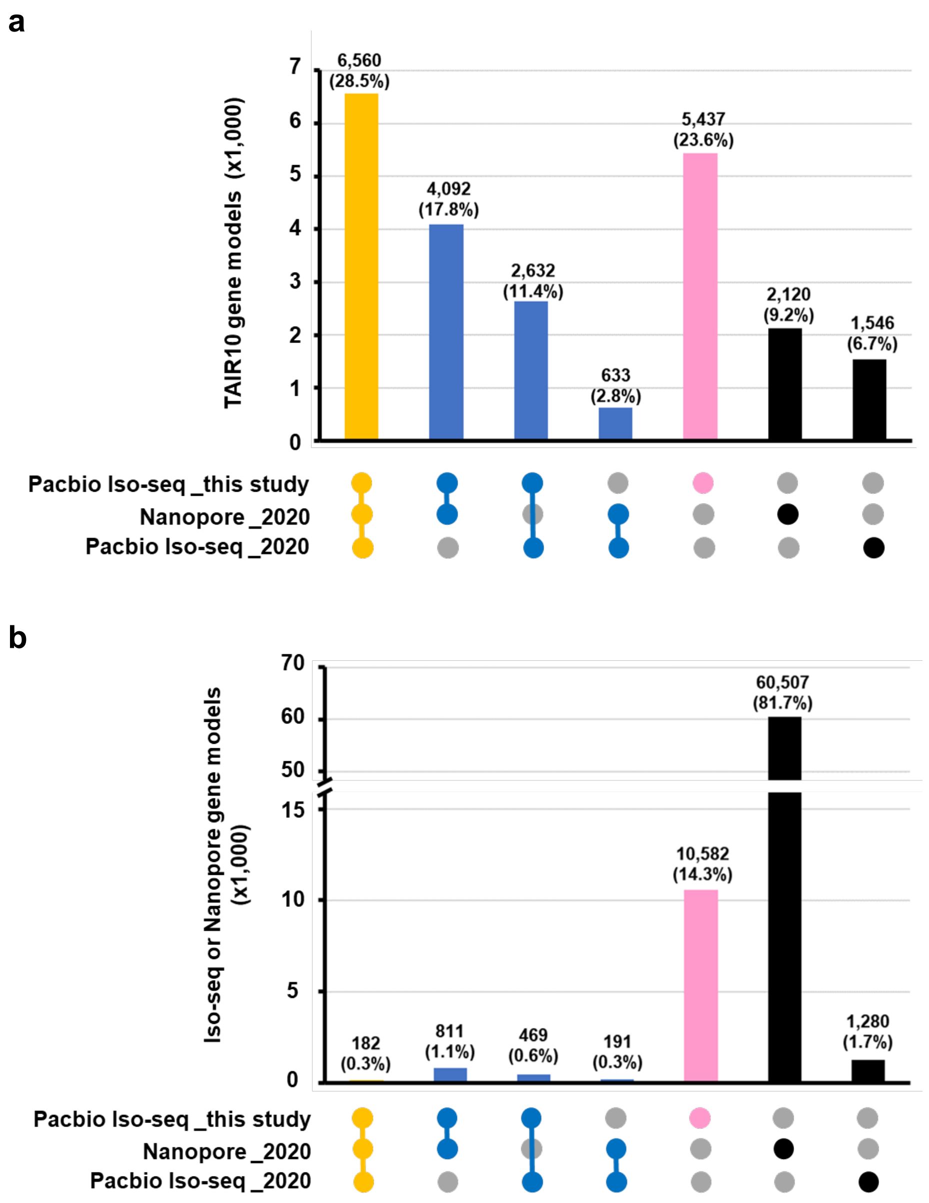

Figure S8. Comparison of gene models identified in three long-read sequencing datasets.

Sequence data from Pacbio Iso-seq_2020 (53) and Nanopore_2020 (54) were analyzed to generate 13,493 and 75,096 gene models, respectively, as described in Methods. Pairwise comparisons of Iso-seq (this study), Pacbio Iso-seq_2020 and Nanopore_2020 were performed for TAIR10 annotated gene models (**a**), Iso-seq or Nanopore gene models **(b)**. Colored filled circles represent datasets compared. Numbers (percentages of all gene models in 3 datasets) of shared or unique gene models for each category are marked on top of each bar.

.

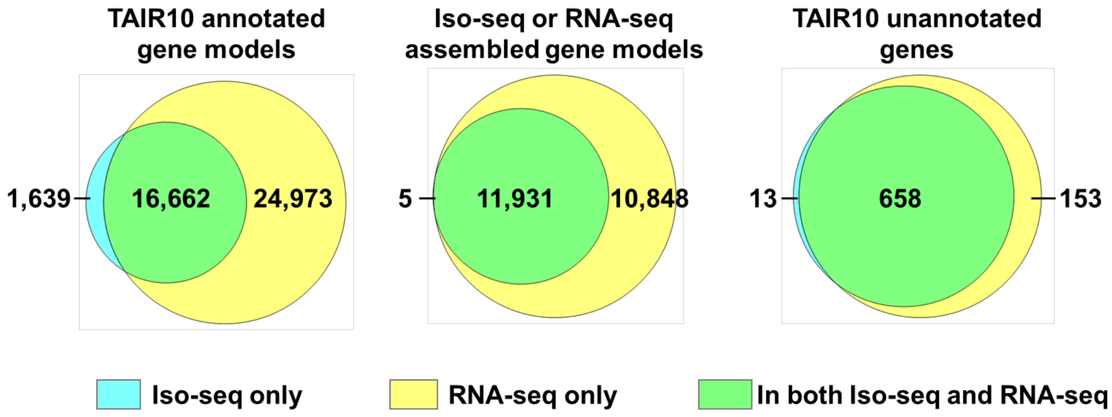

Figure S9. Comparison of gene models identified by Iso-seq and those assembled by RNA-seq data.

RNA-seq data (49) of the same developmental stages were used to assemble gene models as described in Methods. Venn diagrams showing comparisons of gene models identified from Iso-seq or RNA-seq for TAIR10 annotated gene models, Iso-seq or RNA-seq identified gene models and TAIR10 unannotated genes. Approximately ~95% of the Iso-seq derived gene models were supported by RNA-seq.

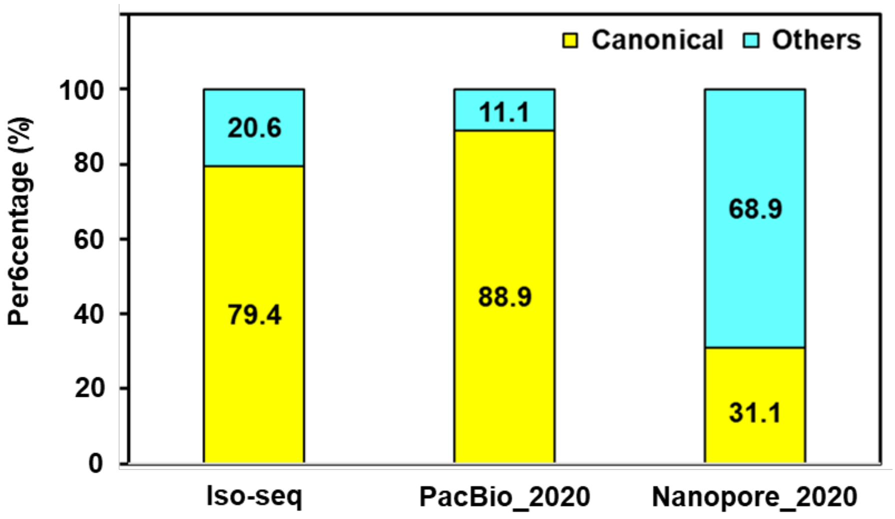

Figure S10. Analyses of canonical GU-AG splicing sites in unique AS events identified in three long-read sequencing datasets.
Percentages of AS events with or without canonical dinucleotide pairs (GU-AG) are shown. Iso-seq (n=9,932), PacBio_2020 (n=2,530), and Nanopore_2020 (n= 60,131).

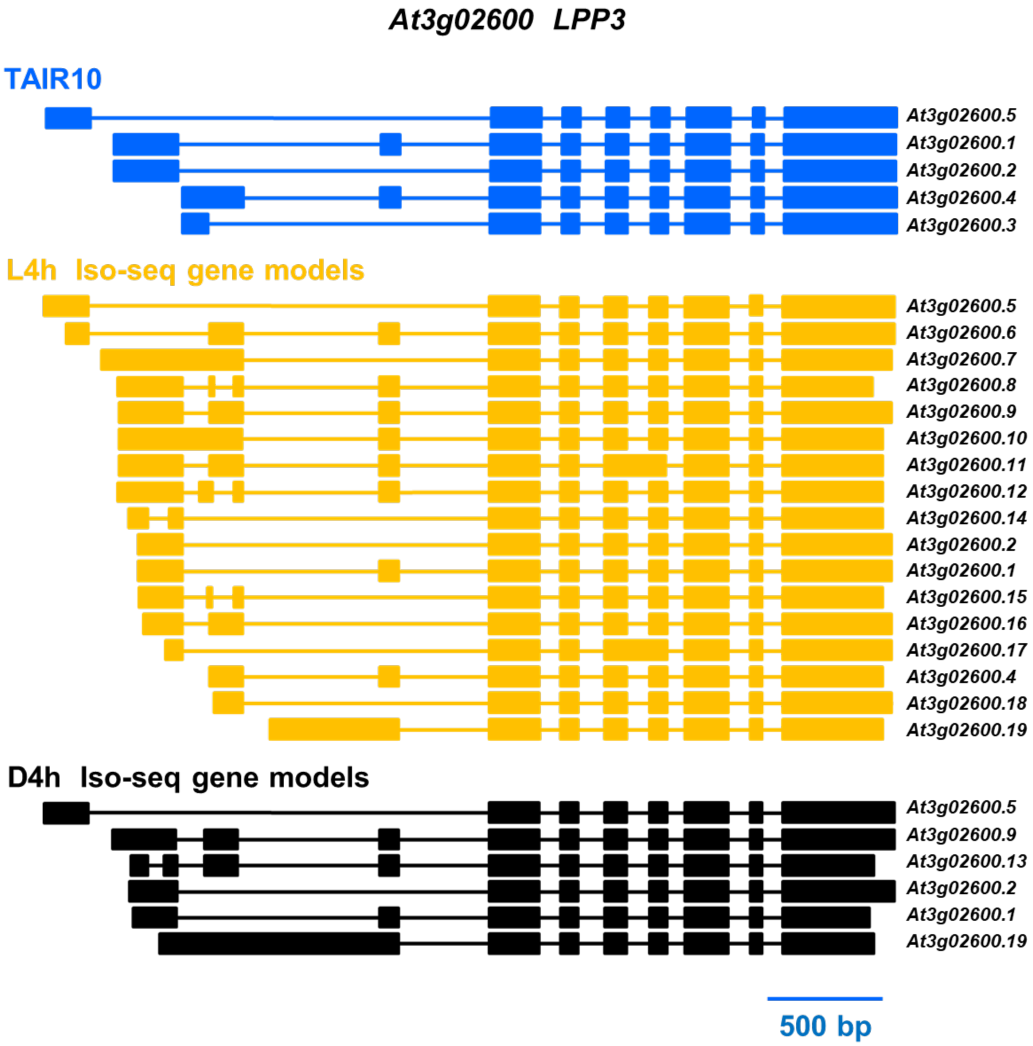

Figure S11. Nineteen alternative splicing isoforms of *At3g02600*.
Five gene models annotated in TAIR10 for *At3g02600*, *LIPID PHOSPHATE PHOSPHATASE 3* (*LPP3*), are shown in blue at the top of the illustration. A total of 17 splicing gene models were detected in the L4h Iso-seq dataset (orange) and 6 in the D4h dataset (black). Exons are represented by filled boxes and introns by lines. Scale bar, 500 bp.

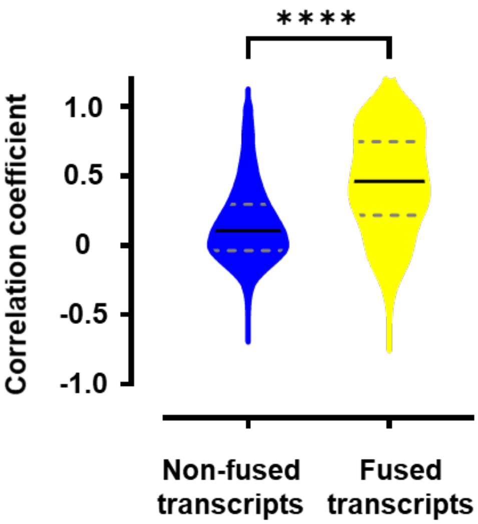

Figure S12. Co-expression analysis of fused or non-fused transcripts.
Greater than 5,000 RNA-seq datasets were download and used for calculating correlation coefficient as described in Methods. Upper dashed line, solid line and lower dashed line represent the 25th, 50th, and 75th percentiles in the distribution of the correlation coefficients. **** p-value <0.0001 (one-way ANOVA with unpaired t-test).

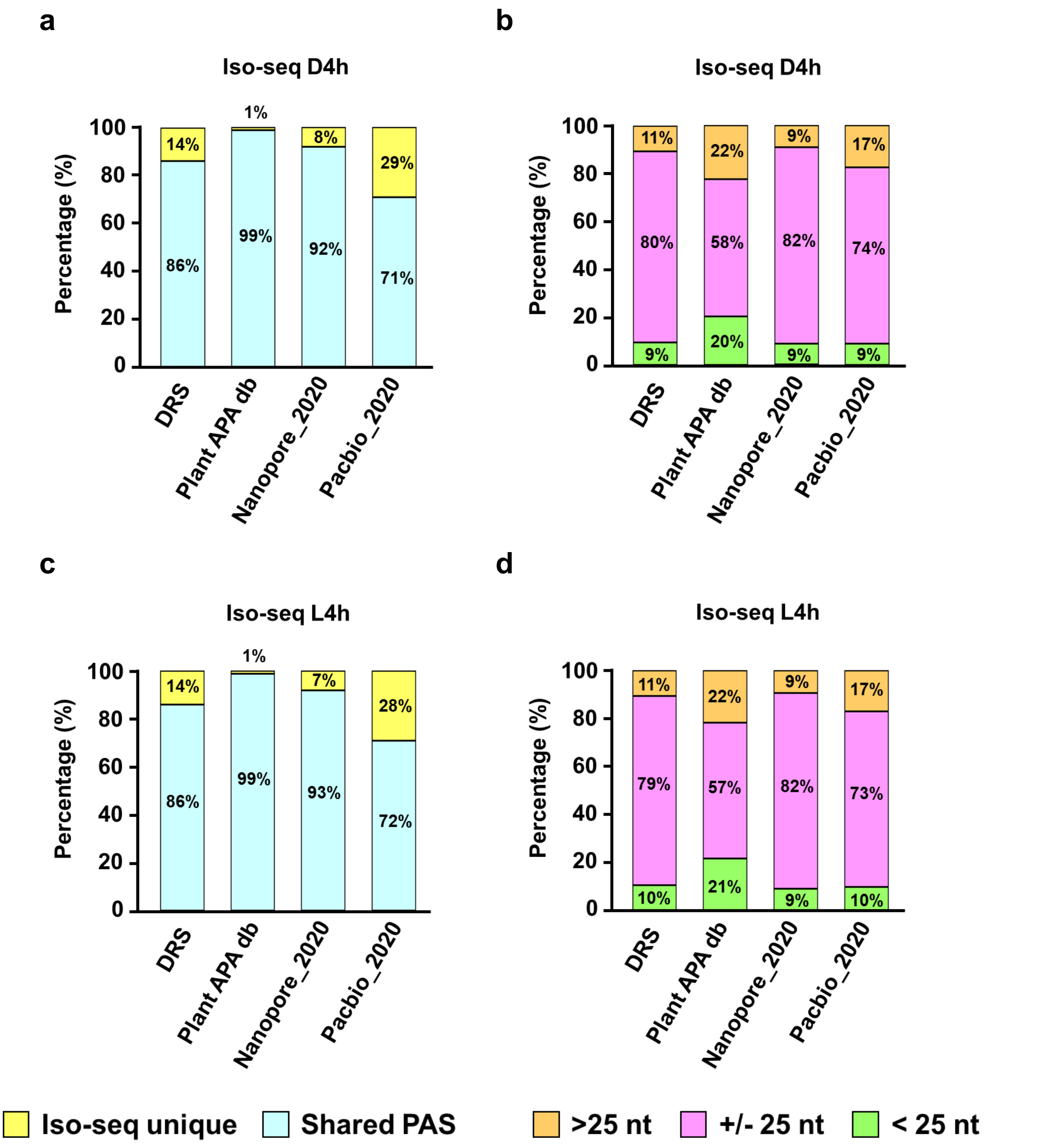

Figure S13. Authenticity of poly(A) sites identified from Iso-seq in this study. Poly(A) sites (PASs) identified from D4h **(a, b)** or L4h **(c, d)** Iso-seq data in this study were compared with those from 4 orthogonal datasets: direct RNA sequencing (DRS) (57), Plant alternative poly-adenylation sites database (Plant APAdb) (58), Nanopore direct RNA sequencing (Nanopore_2020) (54) and Pacbio Isoform sequencing (Pacbio_2020) (53). Shared PASs and those unique in our Iso-seq data are shown in **a, c. b** and **d** show relative PAS positions for the shared ones relative to the other 4 datasets

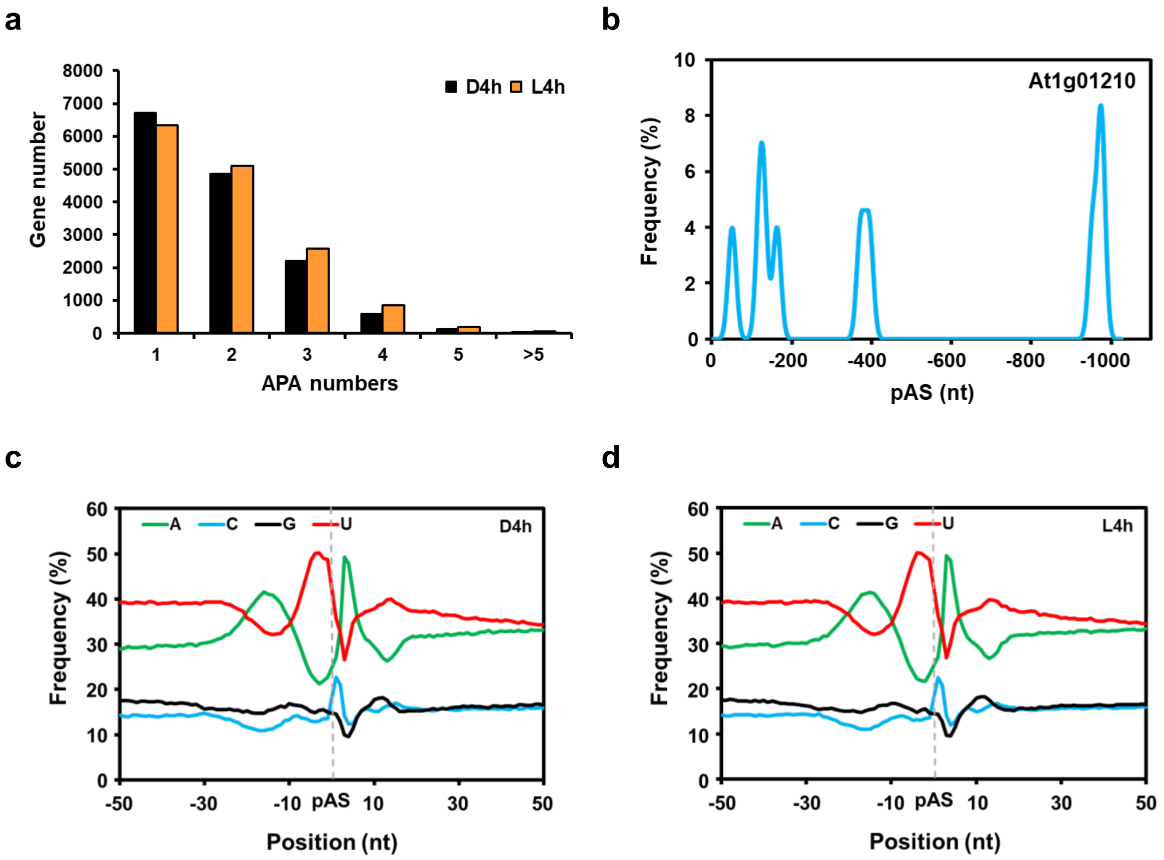

Figure S14. Analysis of alternative polyadenylation sites (APAs) in the Iso-seq datasets.

a Number of APAs per gene. **b** *At1g01210* with 5 polyadenylation site clusters shown as an example. pAS: polyadenylation site. **c, d** Frequency of A, C, G and U surrounding pAS in D4h (**c**) and L4h (**d**) FL datasets.

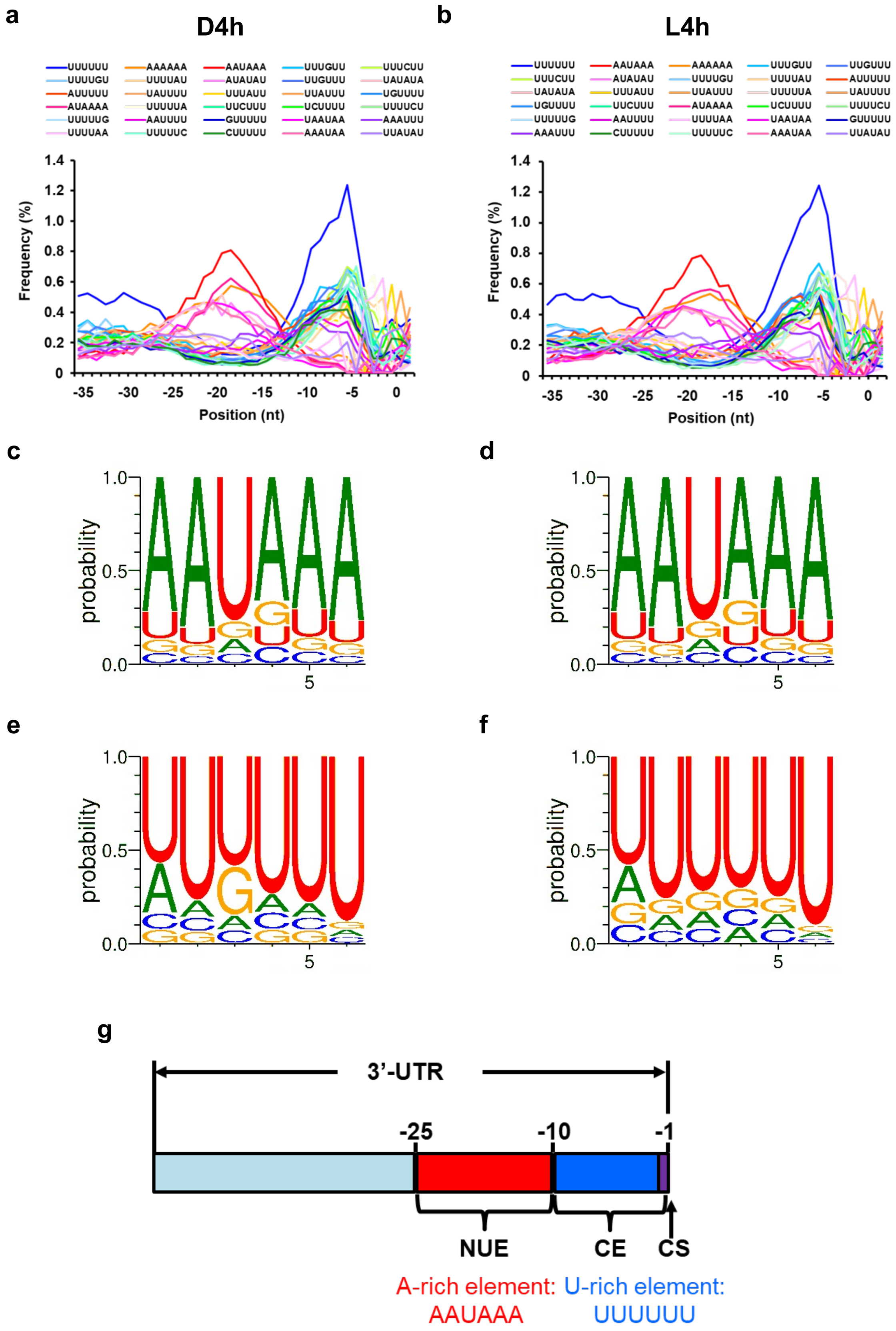
a
D4h
b
L4h
Frequency (%)
Position (nt)
c
d
e
f
probability
g
3'-UTR
-25
-10
-1
NUE
CE
CS
A-rich element:
AAUAAA
U-rich element:
UUUUUU

Figure S15. The enriched near upstream elements (NUEs) and cleavage elements (CEs).

The 72,893 HQ reads from D4h and 87,341 HQ reads from L4h FL dataset were scanned at 6-nt window size across the -35 to +8 positions relative to pAS by using SignalSleuth 2 (86). **a, b** Position distribution of top 30 enriched 6-nt patterns in -35 to +8 relative to pAS. Pattern clusters were enriched at -25 to -10 and - 10 to -1 for both D4h (**a**) and L4h (**b**) datasets. **c, d** An AAUAAA motif as an NUE identified in both D4h (**c**) and L4h (**d**) datasets. **e, f** A UUUUUU motif as a CE identified in both D4h (**e**) and L4h (**f**) datasets. **g** An illustration showing the relative positions and sequences for NUEs and CEs relative to the cleavage site (CS) in 3’ UTRs of transcripts detected in D4h and L4h datasets.

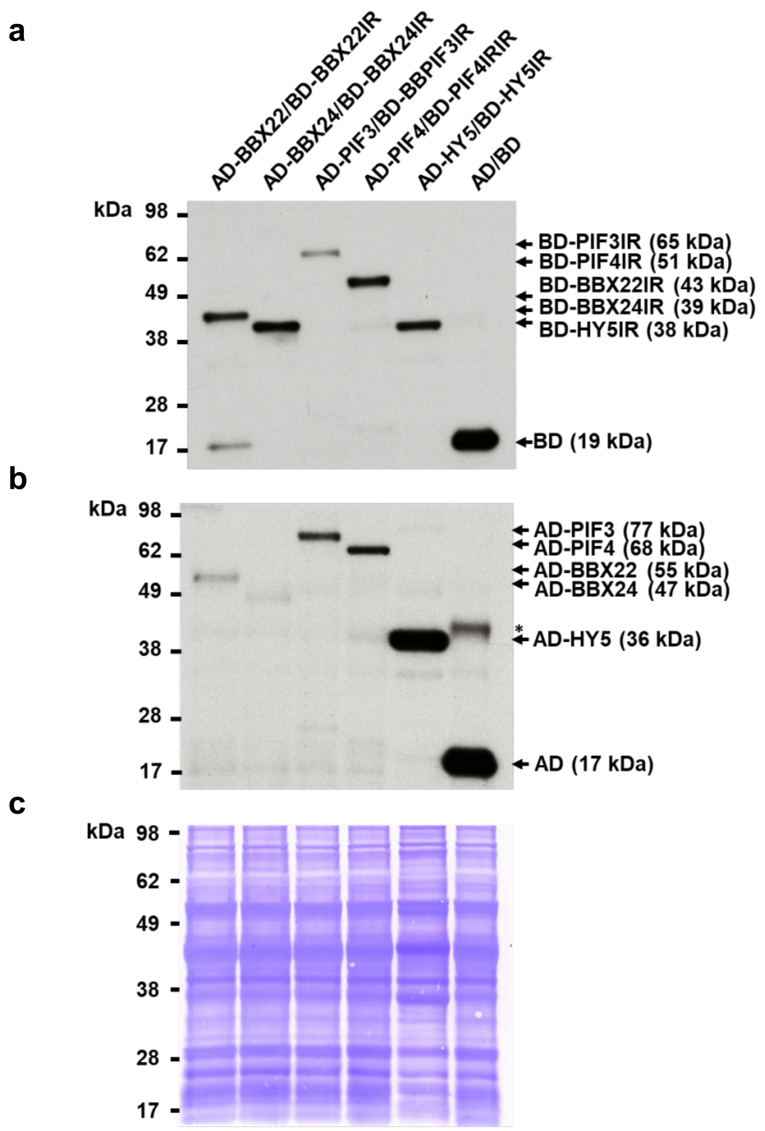

Figure S16. Expression analyses of AD- and BD-fusion proteins in yeast. Total protein lysates were prepared from indicated yeast transformants expressing AD-ABBX22/BD-BBX22IR, AD-BBX24/BD-BBX24IR, AD-PIF3/BD-PIF3IR, AD-PIF4/BD-PIF4IR or AD-HY5/BD-HY5IR and grown in DO-L-W media. **a** Expression of BD-fused IR-form transcription factors. **b** Expression of AD-fused annotated form of transcription factors. **c** Coomassie blue-stained membrane shown as a loading control. Immunoblot analyses were performed with anti-BD (**a**) or anti-AD antisera (**b**). Arrowheads indicate BD, AD, BD- or AD-fusion proteins with expected Mr labeled.

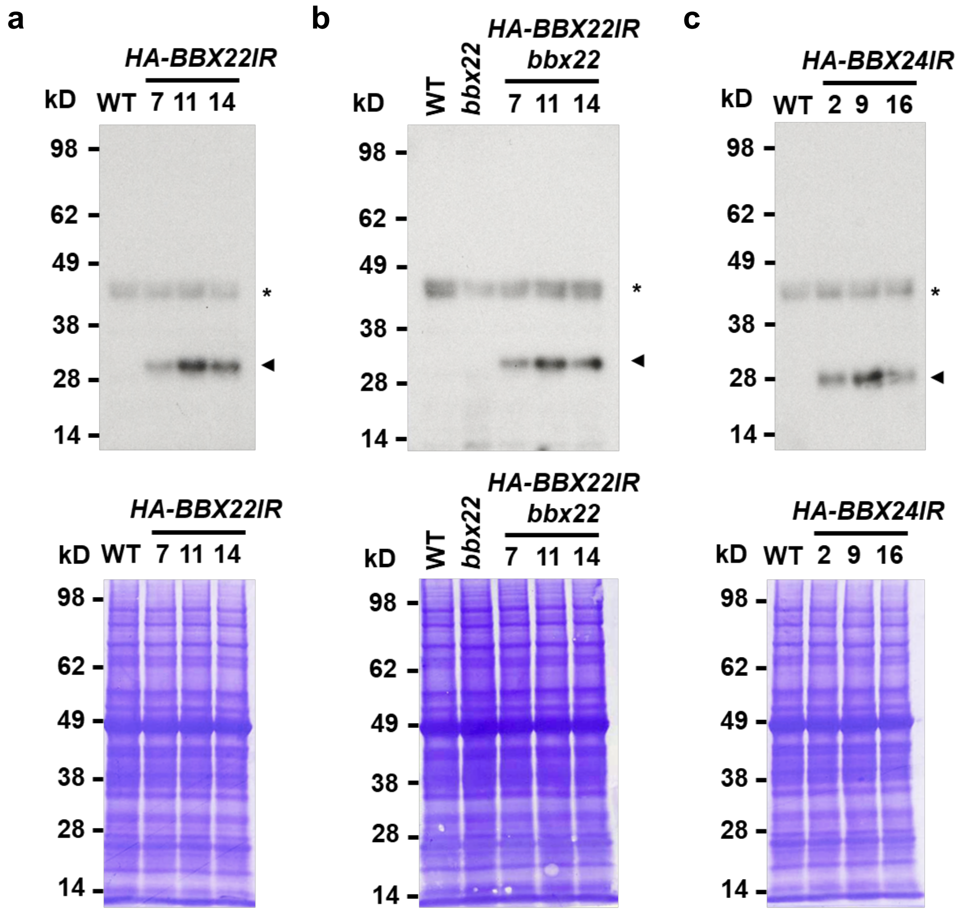

Figure S17. Expression analyses of HA-BBX22IR and HA-BBX24IR in independent transgenic Arabidopsis plants.
Total protein lysates were prepared from 4-h white light-treated 4-d-old de-etiolating seedlings. **a** Expression of BBX22IR in 3 independent *35S:: HA-BBX22IR* transgenic lines. Wild-type (WT) was a negative control. **b** Expression of BBX22IR in 3 independent *bbx22 HA-BBX22IR* cross lines. WT and *bbx22* mutant were negative controls. **c** Expression of BBX24IR in 3 independent *35S::HA-BBX24IR* transgenic lines. WT plant was a negative control. Immunoblot analyses with anti-HA antiserum to detect expression of HA-BBX22IR (**a, b**) and HA-BBX24IR (**c**) indicated by arrowheads. Coomassie blue-stained (CBS) membranes are shown as protein loading controls.

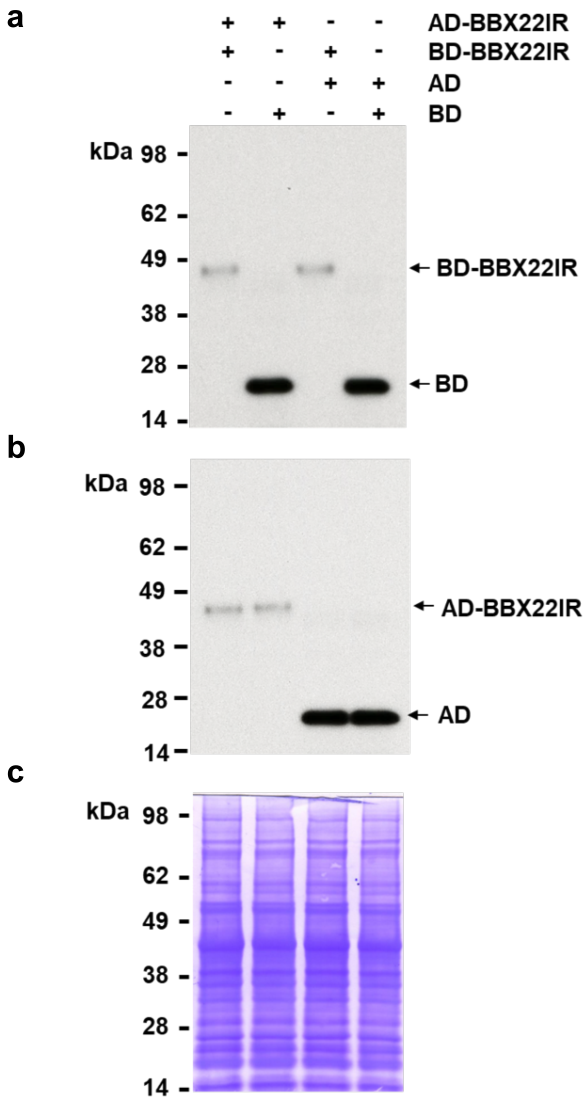

Figure S18. Expression analyses of AD-BBX22IR and BD-BBX22IR in yeast. Total protein lysates were prepared from indicated yeast transformants expressing AD-BBX22IR/BD-BBX22IR, AD-BBX22IR/BD, AD/BD-BBX22IR, or AD/BD grown in DO-L-W medium. **a** Expression of BD and BD-fused BBX22IR. **b** Expression of AD and AD-fused BBX22IR. **c** Coomassie blue-stained membrane as a loading control. Immunoblot analyses were performed with anti-BD (**a**) or anti-AD antisera (**b**). BD or BD-BBX22IR in (**a**) and AD or AD-BBX22IR in (**b**) are marked.

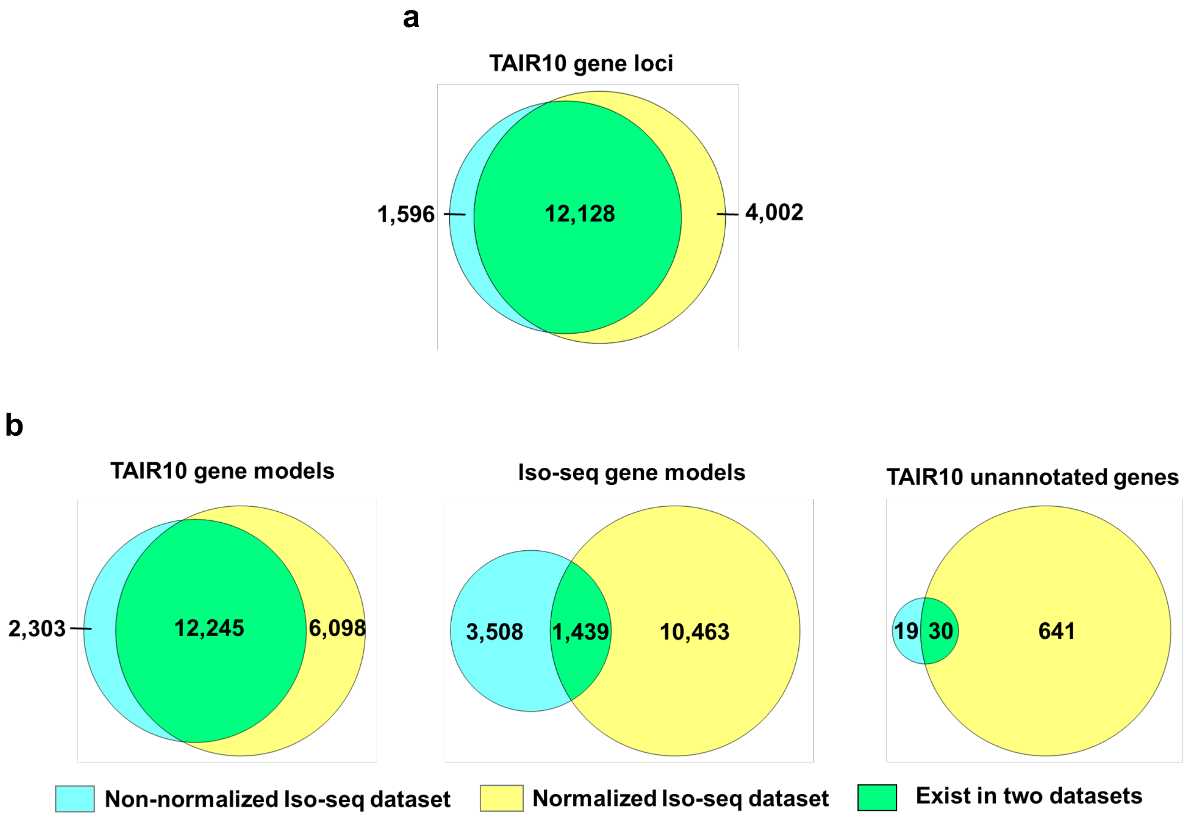

Figure S19. A direct comparison of gene models identified from Iso-seq libraries from cDNA-normalized and non-normalized Iso-seq datasets in gene and gene model identifications.

The total of 1.2 M reads for normalized Iso-seq and 2 M reads for non-normalized Iso-seq (including D4h and L4h) were used to identify FL transcript isoforms in two biological replicates. **a** Comparison of identified gene loci between cDNA-normalized and non-normalized Iso-seq datasets. **b** Comparison of identified gene models and unannotated genes between cDNA-normalized and non-normalized Iso-seq datasets.

a

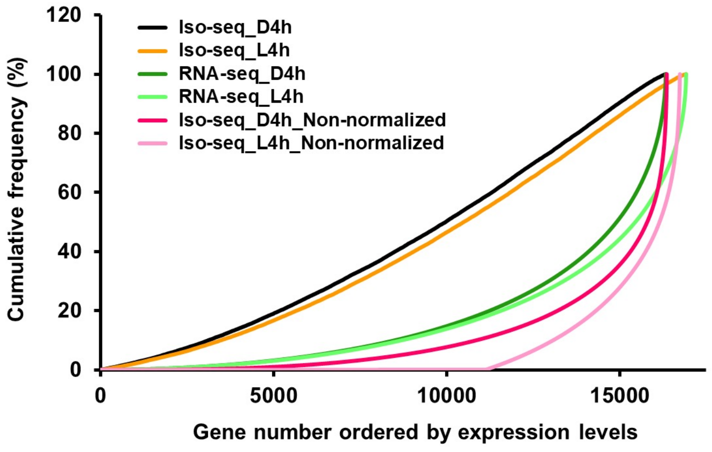

b

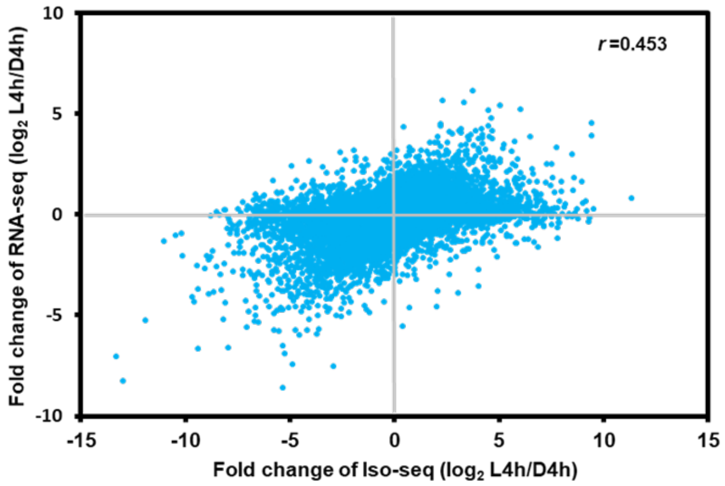

Figure S20. A direct comparison of gene expression and differential gene expression in RNA-seq, normalized Iso-seq and non-normalized Iso-seq data.

a Cumulative frequencies of expressed genes in RNA-seq (49), cDNA-normalized and non-normalized Iso-seq datasets were plotted. **b** Correlation of differential gene expression (L4h/D4h, log2 transformed) between RNA-seq and non-normalized Iso-seq data. The gene expression levels of non-normalized D4h/L4h Iso-seq datasets were calculated by counts per million reads (CPM). The gene expression levels of RNA-seq datasets were calculated by reads per kilobase per million (RPKM). r: Pearson correlation coefficient (r).

a

b

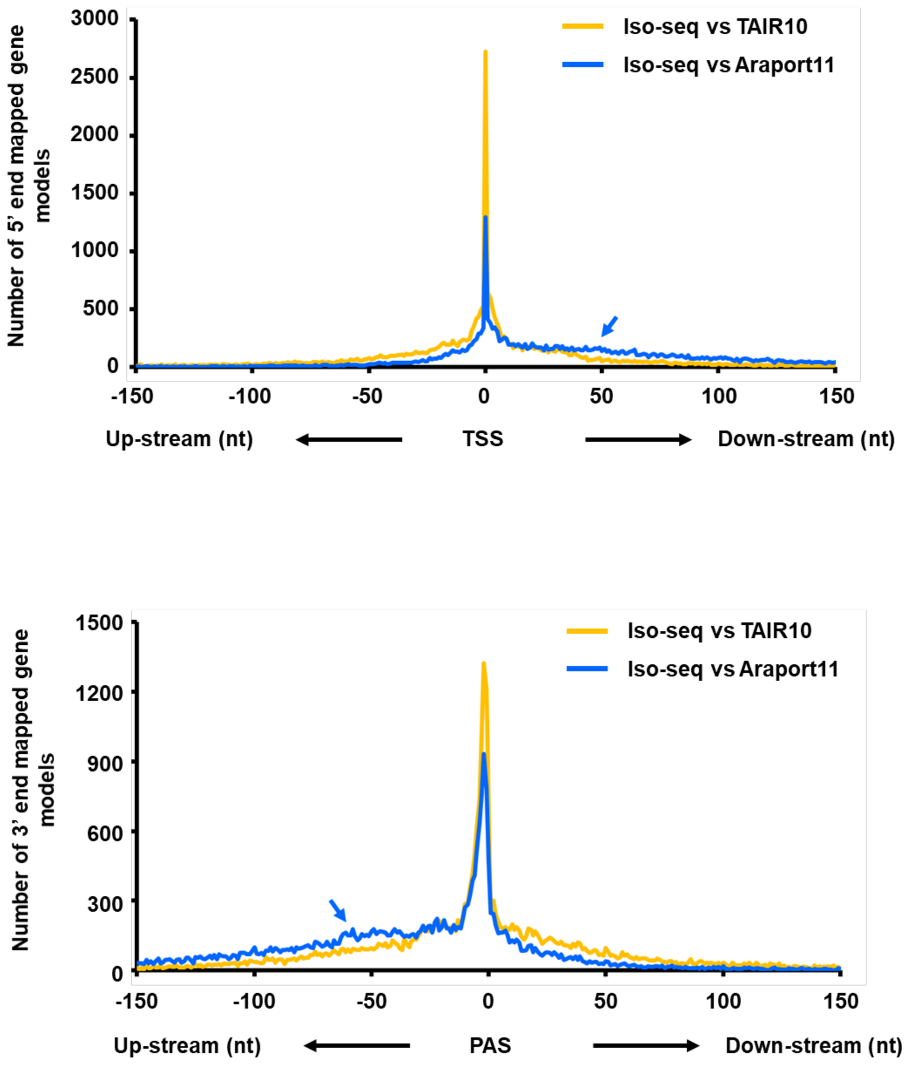

Figure S21. Alignments of 5' and 3' ends of gene models from Iso-seq datasets with those in TAIR10 and Araport11.

All gene models from Iso-seq data in this study were used to compare with those in TAIR10 or Araport11 for the relative positions of the transcription start site (TSS) in 5' ends (**a**) or polyadenylation site (PAS) in 3' ends (**b**). Blue arrowheads indicate that the total gene models identified in Iso-seq are shorter than those annotated in the Araport11.
