# Supplementary material for: An improved repertoire of splicing variants and their potential roles in Arabidopsis photomorphogenic development
Source: Genome Biol. 2022 Feb 9;23:50. doi: 10.1186/s13059-022-02620-2 (PMC8827149; doi:10.1186/s13059-022-02620-2)

Figure 4d uncropped images

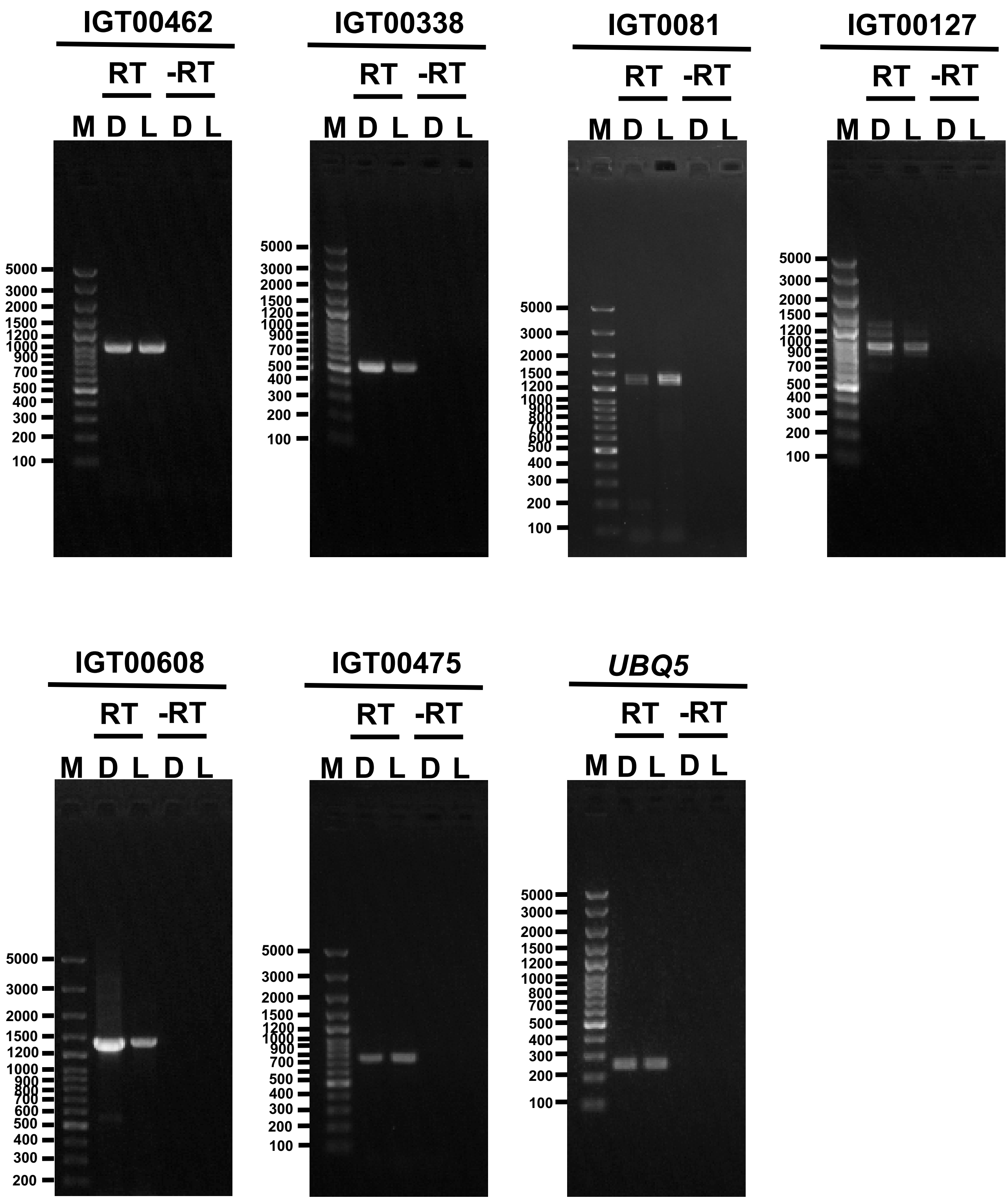

Figure 4e uncropped images

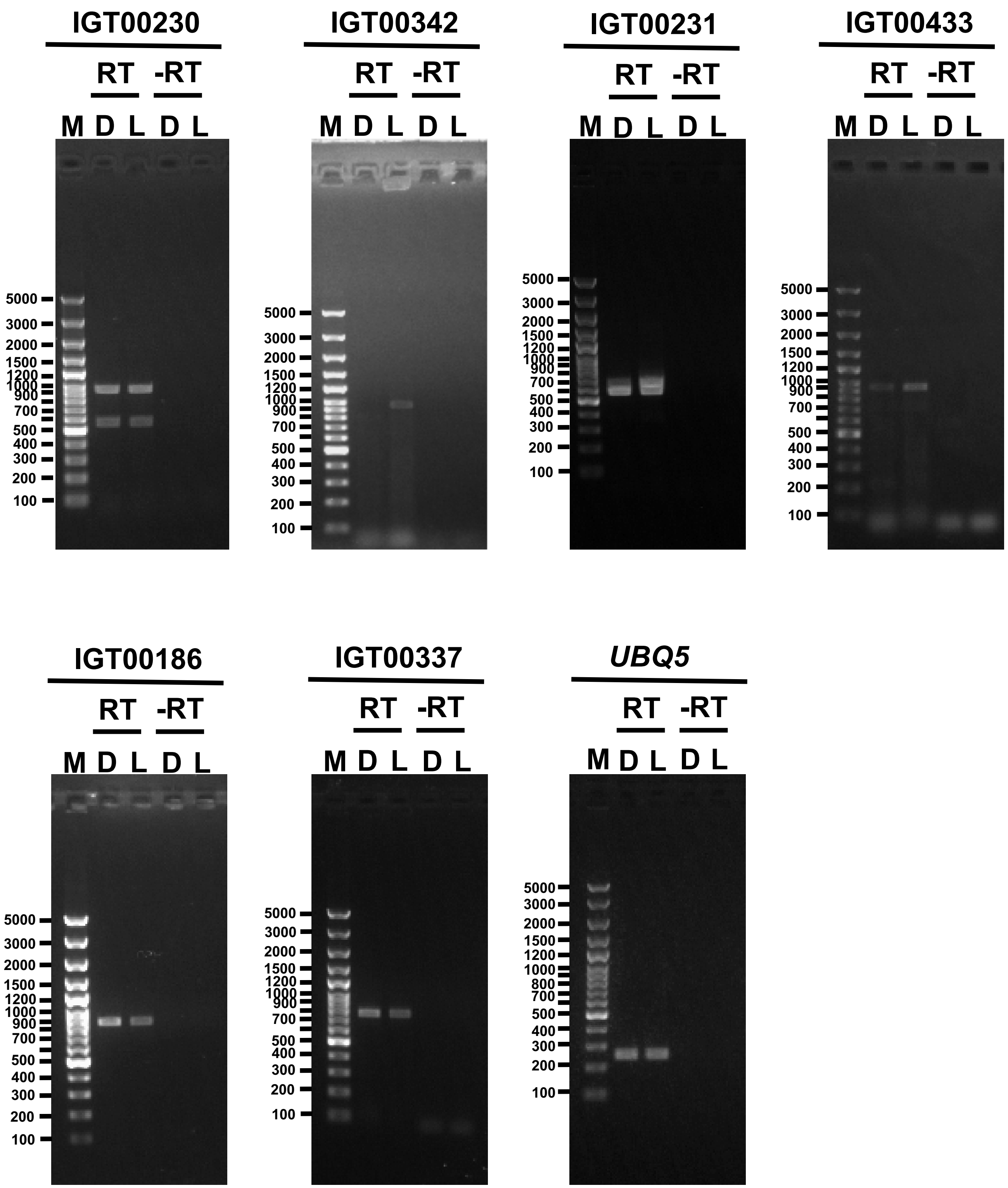

Figure 5c, d and e uncropped images

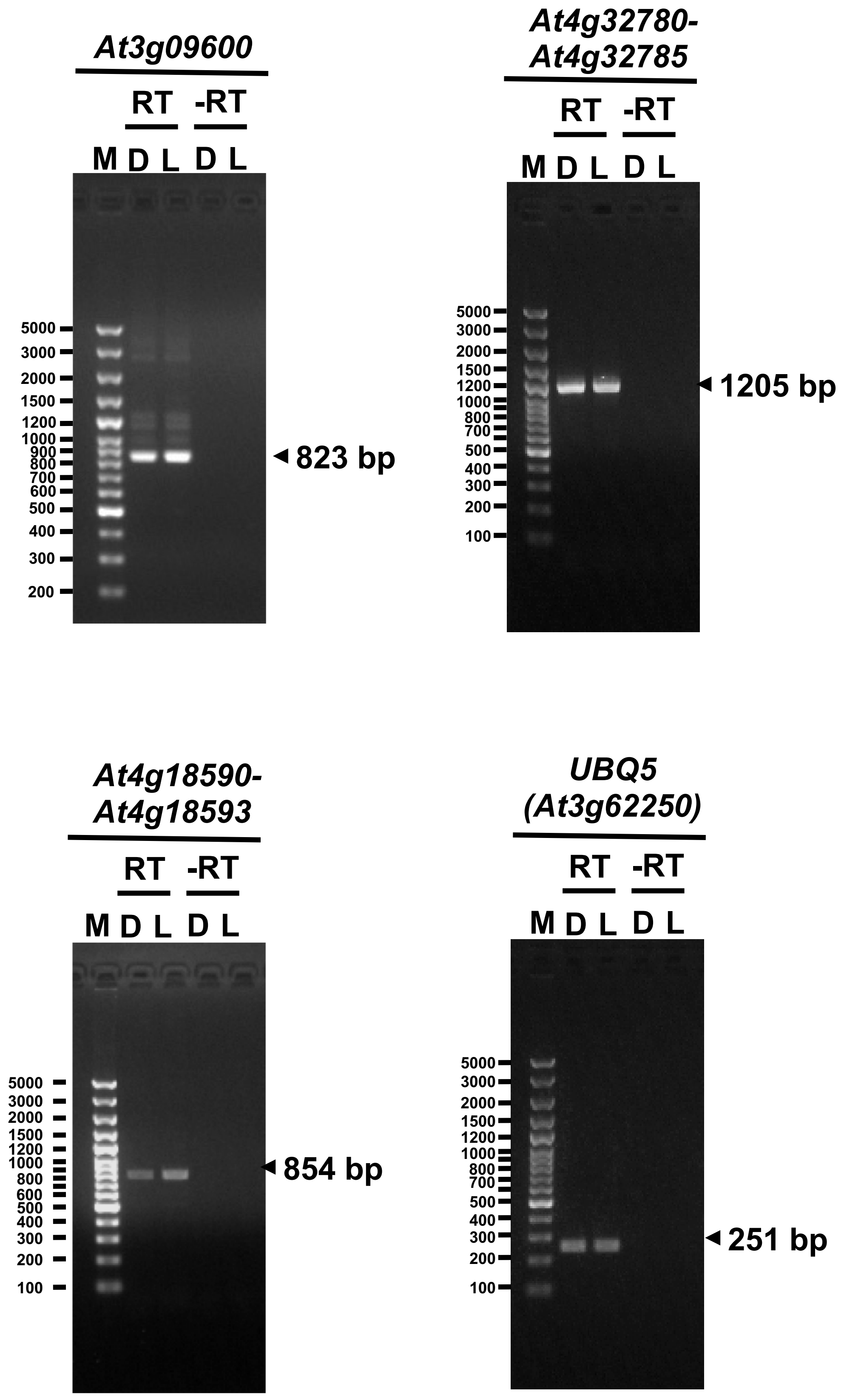

Figure 7d uncropped blots and gel image

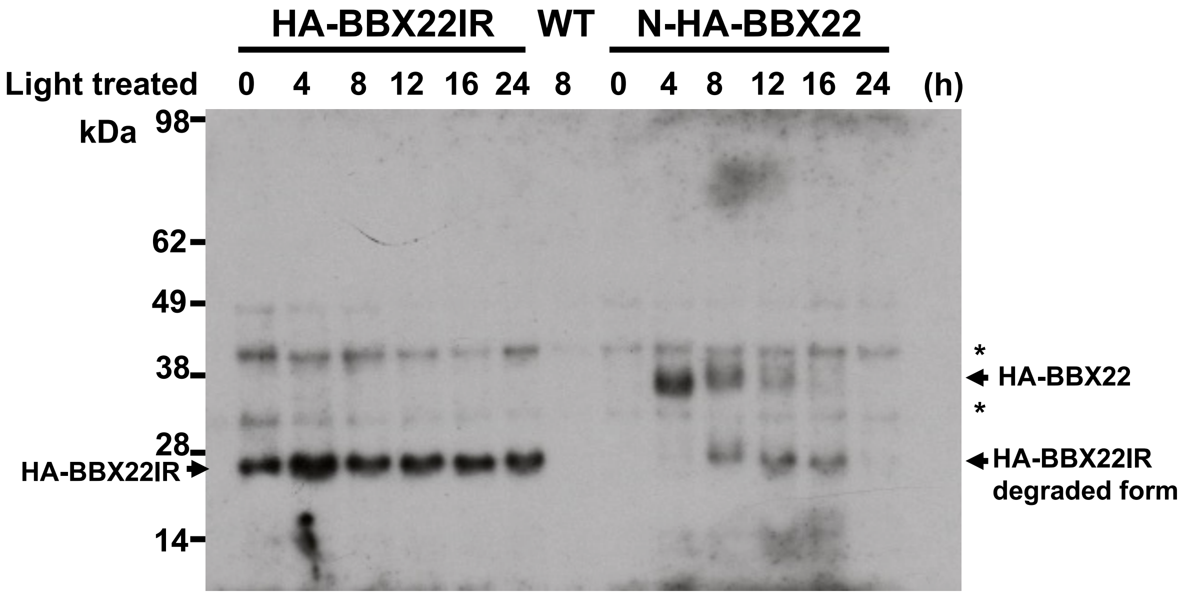

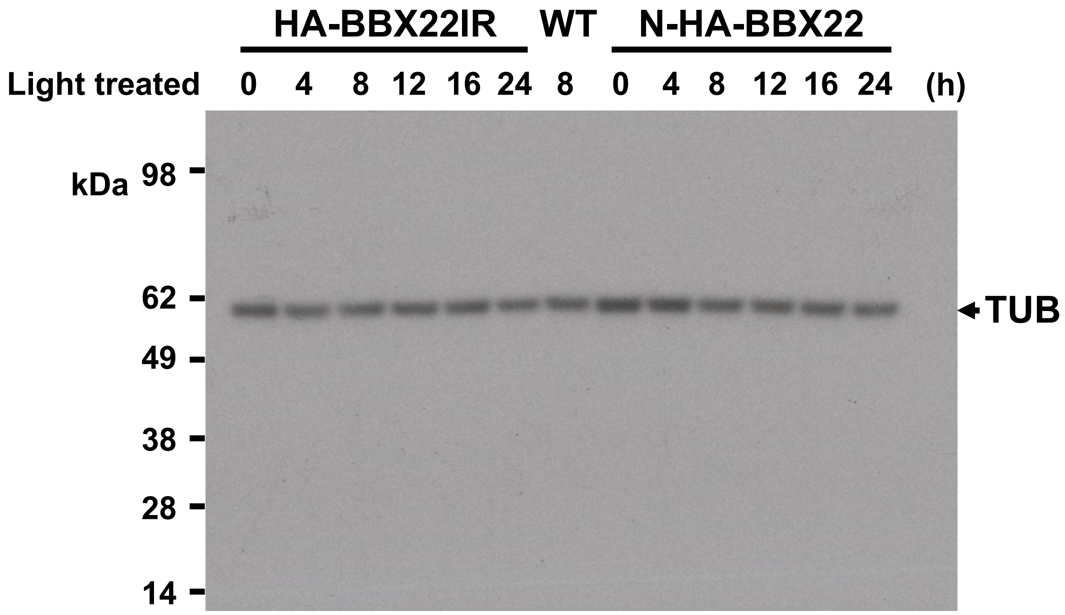

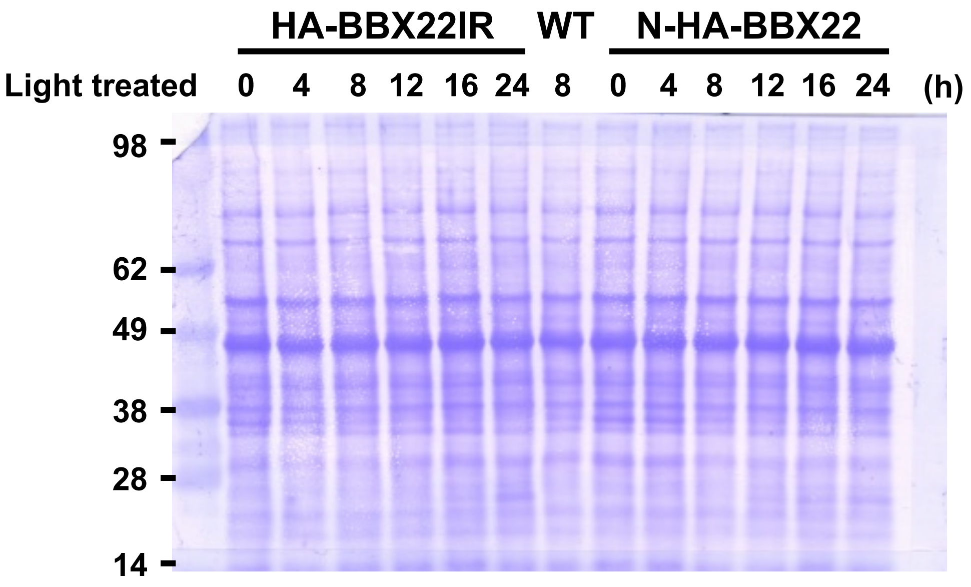

Supplement: Supplementary file 15 — Additional file 15. Uncropped blots for Figures 4, 5 and 7. [file 13059_2022_2620_MOESM15_ESM.pdf]
