# Supplementary material for: An improved repertoire of splicing variants and their potential roles in Arabidopsis photomorphogenic development
Source: Genome Biol. 2022 Feb 9;23:50. doi: 10.1186/s13059-022-02620-2 (PMC8827149; doi:10.1186/s13059-022-02620-2)
Supplement: Supplementary file 16 — Additional file 16. Review history. [file 13059_2022_2620_MOESM16_ESM.docx]

Review History

**First round of review**

**Reviewer 1**

**Are you able to assess all statistics in the manuscript, including the appropriateness of statistical tests used?**

There are no statistics in the manuscript.

**Comments to author:**

This manuscript describes a PacBio iso-seq analysis of light and dark-treated Arabidopsis seedlings. PacBio has been used in previous Arabidopsis RNA-seq studies. A double stranded cDNA degradation normalisation approach is used which has the advantage of enabling the detection of rarer or different transcripts, but the disadvantage that quantitative information is lost. The number of reads is relatively low (approx. 150K for each condition - p5- l51), but this is counterbalanced by the normalisation procedure, and evidence is provided for the detection of new Arabidopsis transcript isoforms and unannotated genes. Consequently, this is a useful resource for the Arabidopsis field.

The study includes some analysis of different 5' ends but is somewhat limited because it does not distinguish full-length capped RNAs from potential 5' truncated transcripts, resulting either from decay or library preparation (p6-l55) - ie there is no validation of such 5' ends. There are several datasets that could be used to validate whether the 5' ends of the transcripts they detect are genuine, in particular, nanoPARE, developed by Schon et al. 2018 (Genome Research).

The study also includes analysis of alternative polyadenylation. A limitation of the poly(A) site study (p10) is that a more in depth analysis of motifs associated not only with poly(A) sites but also ranked according to poly(A) site usage was already published way back in 2012 by Sherstnev et al., Nat Struct Mol Biol, but this study is not cited by the authors. If the authors intend only to validate the authenticity of the 3' ends identified by the PacBio approach, this previous work should be cited and possibly used as an orthogonal dataset.

The authors focus on the potential functional significance of an alternative splicing event. Intron retention is detected in BBX22. Intron retention is a common form of alternative splicing detected in Arabidopsis, but other studies have shown that it typically comprises a relatively small % of transcripts processed from the same gene (see papers from the Barta Lab, for example). This also appears to be the case here, with this isoform representing less than 5% of the BBX22 transcripts (Figure 6a). The authors test the potential significance of this intron retention by first assessing protein interactions using Y2H, but also using transgenic over-expression of the intron retained isoform. There are two main problems with this study. First, the authors do not demonstrate that the intron retained isoform actually expresses a protein in wild type Arabidopsis plants. This is important because there is evidence that intron retained transcripts are held in the nucleus. If this isoform was exported it may be targeted for decay by NMD. The second problem is that the phenotypic effects could simply be a gain of function from over-expression (especially considering that according to the authors' data this isoform is normally expressed at less than 5% of the spliced isoform). The better way to test the functional significance of alternative splicing would be to remove this intron from a test transgene, so that alternative splicing was not possible, introduce into the bbx22 mutant background and then ask if there is a detectable impact on phenotype. This is important because this is the conceptual focus of the paper and accounts for the title: "Splicing variants constitute novel regulators of photomorphogenic development in Arabidopsis"

A weakness of the RNA validation experiments is an absence of minus reverse transcriptase controls - see Figures 4d,e; 5c,d,e.

Indeed, the authors do not comment on whether some of the many apparently non-coding RNAs they detect could be explained by RT-dependent template switching, which is an established disadvantage of cDNA sequencing. No non-RT-based validation of novel ncRNAs is performed here.

**Reviewer 2**

**Are you able to assess all statistics in the manuscript, including the appropriateness of statistical tests used?**

Yes, and I have assessed the statistics in my report.

**Comments to author:**

This manuscript entitled "Splicing variants constitute novel regulators of photomorphogenic development in Arabidopsis" presents here a description about normalized cDNA libraries and PacBio Iso-seq to identify new gene models and post-transcriptional regulation. This study also revealed the regulatory function of two IR isoforms, BBX22IR and BBX24IR, in photomorphogenic development. This resource will likely inspire further research for functional characterization of splicing isoforms in photomorphogenic development.

I would like to recommend the following revision.

In this study, normalized cDNA libraries present great advantage for identification of rare isoforms with low abundance. However, normalized cDNA libraries also had great disadvantage in quantitative profiling of isoforms in response to light signals. Thus, common cDNA libraries without normalization for PacBio Iso-seq is highly required for D4h and L4h sample to find light-induced alt-TSSs or alt-TESs, alternative polyadenylation (APA), and Alternative splicing (AS).

This manuscript reported "~12,000 new gene models and ~700 unannotated models" by comparing with TAIR10 to classify it into TAIR10 gene models, Iso-seq gene models and TAIR10 unannotated genes. However, the gene models from TAIR10 were outdate. New gene models reported in this study might have been included in published studies using long-read technologies. The authors can compare these new models in this study with up-to-date studies using PacBio SMRT (Cui, Jiawen, et al. "Analysis and comprehensive comparison of PacBio and nanopore-based RNA sequencing of the Arabidopsis transcriptome." Plant Methods 16.1 (2020): 1-13) or Nanopore sequencing (Parker, Matthew T., et al. "Nanopore direct RNA sequencing maps the complexity of Arabidopsis mRNA processing and m6A modification." Elife 9 (2020): e49658.).

P5 Line 55 "Therefore, the cDNA normalization allowed for effective detection of rare transcripts." The authors cannot get this conclusion based on only the number of gene loci in this section, since this study did not sequence common cDNA libraries without normalization for PacBio Iso-seq as control. However, next section in the manuscript indeed verified the success of cDNA normalization.

Figure 3: New models (5787 D4h, 7401 L4h new and 671 TAIR10 unannotated genes) in this studies have great probability to be caused by tissue-specific expression in D4h and L4h, which did not annotated by TAIR10. For this situation, this kind of new model was caused by tissue-specific expression rather than the advantage of PacBio iso-seq long reads or the success of cDNA normalization. Thus Figure 3 can contain control from using published RNA-seq data (Liu, Ming-Jung, et al. "Translational landscape of photomorphogenic Arabidopsis." The Plant Cell 25.10 (2013): 3699-3710.) to reconstruct gene models using transcript assembly software, such as cufflinks or stringtie.

Page 8 Line 22 Is it possible that shorter CDS is caused due to the error of PacBio (Figure S5) if the author used PacBio sequencing for CDS prediction?

Page 9 Line 30 RT-PCR revealed several transcripts in etiolated or de-etiolating seedling. The author can present more useful information if they identify and present all novel transcripts in response to light (Differential transcripts between D4h and L4h) using published RNA-seq data (Liu, Ming-Jung, et al. "Translational landscape of photomorphogenic Arabidopsis." The Plant Cell 25.10 (2013): 3699-3710.) or adding common cDNA libraries without normalization for PacBio Iso-seq.

Figure 4d/e: Reference genes such as UBQ should be added in Figure 4d/e to normalize two samples.

From the gene structure in the left panel of Figure 4e, the transcript of IGT00230 generated from single exon gene. Why did RT-PCR present two bands?

Figure 5b: How many AS events were differential between D4h and L4h. RNA-seq from (Liu, Ming-Jung, et al. "Translational landscape of photomorphogenic Arabidopsis." The Plant Cell 25.10 (2013): 3699-3710.) or common cDNA libraries without normalization for PacBio Iso-seq can address this question.

Page 10, Line 9-10: "254 showed evidence of sequences spanning separately annotated transcripts in TAIR10.". This study only presented two case studies in Figure 5d, e to support the fusion of pair-wise transcripts. The author can provide comprehensive evidence for the 254 fusion transcripts. Using one pair-wise fusion transcripts (At4g32780 and At4g32785) as example, the author may observe that pair-wise fusion transcripts have similar expression profiles due to coordinate expression in different tissues. Thus, the author can calculate expression level (FPKM/RPKM) based RNA-seq from different studies for all the genes. Then Pearson's correlation coefficient (PCC) for each pair of fusion transcripts (such as At4g32780 and At4g32785) can be calculated. If PCC from most of the pair-wise fusion transcripts presents strong bias to positive correlation, which will further support that they indeed originate from fusion transcripts.

Fig. S8a How many APA events were supported by published PacBio Iso-seq (Cui, Jiawen, et al. "Analysis and comprehensive comparison of PacBio and nanopore-based RNA sequencing of the Arabidopsis transcriptome." Plant Methods 16.1 (2020): 1-13), Nanopore DRS (Parker, Matthew T., et al. "Nanopore direct RNA sequencing maps the complexity of Arabidopsis mRNA processing and m6A modification." Elife 9 (2020): e49658.) and PAT-seq (Wu, X., Liu, M., Downie, B., Liang, C., Ji, G., Li, Q. Q., & Hunt, A. G. (2011). Genome-wide landscape of polyadenylation in Arabidopsis provides evidence for extensive alternative polyadenylation. Proceedings of the National Academy of Sciences, 108(30), 12533-12538.) ?

Page 11, line 26-46, in this section, the author only presented 5 IR variants. In total, how many TFs with IR variants including AS events were light induced and were associated with translation machinery?

Page 12 44-46 The label of b and c should be switched in Figure7 or main document.

Methods:

The authors cloned FL representative transcripts (short isoforms) for BBX22, BBX24 and their intron retained transcripts (long isoforms) BBX22IR, BBX24IR into vectors. In fact, the retained intron from BBX22IR, BBX24IR included normal GT-AG splicing sites. Thus, overexpressing BBX22IR and BBX24IR line still can generate both retained transcripts and spliced transcripts (short isoforms). The author should check this potential possibility in BBX22IR and BBX24IR overexpressing lines. Mutation the 5′ and 3′ splice sites of the retained intron can only generate intron retained transcripts (long isoforms) without the interference of short isoforms.

The authors should deposit all the PacBio sequencing data in public database (such as NCBI) and cited the accession in manuscript.

**Authors Response**

**Point-by-point responses to the reviewers’ comments:**

**Reviewer #1:**

This manuscript describes a PacBio iso-seq analysis of light and dark-treated Arabidopsis seedlings. PacBio has been used in previous Arabidopsis RNA-seq studies. A double stranded cDNA degradation normalisation approach is used which has the advantage of enabling the detection of rarer or different transcripts, but the disadvantage that quantitative information is lost. The number of reads is relatively low (approx. 150K for each condition - p5- l51), but this is counterbalanced by the normalisation procedure, and evidence is provided for the detection of new Arabidopsis transcript isoforms and unannotated genes. Consequently, this is a useful resource for the Arabidopsis field.

The study includes some analysis of different 5' ends but is somewhat limited because it does not distinguish full-length capped RNAs from potential 5' truncated transcripts, resulting either from decay or library preparation (p6-l55) - ie there is no validation of such 5' ends. There are several datasets that could be used to validate whether the 5' ends of the transcripts they detect are genuine, in particular, nanoPARE, developed by Schon et al. 2018 (Genome Research).

*Response: We thank the reviewer for the very constructive comment. As suggested, we have compared the 5’ ends of our HQ FL reads with those in nanoPARE datasets [1] and in TAIR10. More than 80% of our Iso-seq reads matched or were longer than those in nanoPARE or TAIR10. The new analyses have been included in the revised manuscript (Additional file 1: Fig. S7).*

The study also includes analysis of alternative polyadenylation. A limitation of the poly(A) site study (p10) is that a more in depth analysis of motifs associated not only with poly(A) sites but also ranked according to poly(A) site usage was already published way back in 2012 by Sherstnev et al., Nat Struct Mol Biol, but this study is not cited by the authors. If the authors intend only to validate the authenticity of the 3' ends identified by the PacBio approach, this previous work should be cited and possibly used as an orthogonal dataset.

*Response: We thank the reviewer for pointing out this paper we neglected to cite. As suggested, we have cited this paper to further strengthen our observation in the revised manuscript. Sherstnev et al. (2012) [2] investigated the AAUAAA motif with a 1-nt substitution at the near upstream element (NUE) and the UUGUUU motif with a 1-nt substitution at the cleavage element (CE). However, our study aimed to identify overrepresented motifs by looking for hexamers enriched in NUE and CE. Despite the different approaches, among the top 5 elements we observed in NUE regions, AAUAAA (14%), AAAAAA (12%), AUAAAA (12%), AUAUAU (10%), and UAAUAA (8%) in NUE and the UUUUUU motif in CE, represent variants of the AAUAAA and UUGUUU as described by Sherstnev et al. (2012). The results are shown in Additional file 1: Fig. S15 of the revised manuscript.*

*We further compared whether the poly(A) sites identified in our study were consistent with those revealed by 4 datasets from direct RNA sequencing (DRS) [2], the Plant APA database (PlantAPAdb) [3], and Nanopore and Pacbio Iso-seq [4, 5]. Most of the PASs identified in our Iso-seq data have counterparts in these 4 datasets, which further supports the authenticity of the 3' ends identified by our PacBio approach. These analyses are included in Additional file 1: Fig. S13 of the revised manuscript.*

The authors focus on the potential functional significance of an alternative splicing event. Intron retention is detected in BBX22. Intron retention is a common form of alternative splicing detected in Arabidopsis, but other studies have shown that it typically comprises a relatively small % of transcripts processed from the same gene (see papers from the Barta Lab, for example). This also appears to be the case here, with this isoform representing less than 5% of the BBX22 transcripts (Figure 6a). The authors test the potential significance of this intron retention by first assessing protein interactions using Y2H, but also using transgenic over-expression of the intron retained isoform. There are two main problems with this study. First, the authors do not demonstrate that the intron retained isoform actually expresses a protein in wild type Arabidopsis plants. This is important because there is evidence that intron retained transcripts are held in the nucleus. If this isoform was exported it may be targeted for decay by NMD. The second problem is that the phenotypic effects could simply be a gain of function from over-expression (especially considering that according to the authors' data this isoform is normally expressed at less than 5% of the spliced isoform). The better way to test the functional significance of alternative splicing would be to remove this intron from a test transgene, so that alternative splicing was not possible, introduce into the bbx22 mutant background and then ask if there is a detectable impact on phenotype. This is important because this is the conceptual focus of the paper and accounts for the title: "Splicing variants constitute novel regulators of photomorphogenic development in Arabidopsis".

*Response: We thank the reviewer for the insightful comments. Indeed, previous studies reported that many AS variants are retained within the nuclei. However, the IR variants we analyzed in Fig. 6, including BBX22IR and BBX24IR, showed evidence of escaping nuclei because they can be identified in ribosome protected fragments (Additional file 10: Table S9) [6] and therefore reside in the cytosol. This also implies that these IR variants can potentially undergo translation. BBX22IR, BBX24IR, PIF3IR and HY5IR are unlikely targets of NMD because they are not included in 3 recent datasets for UPF1, UPF3 and SMG7 [7-9].*

*We also agree that it would be the best to detect the protein from BBX22IR, and we invested tremendous efforts and time on this. To confirm the expression of the BBX22IR protein isoform, we synthesized an isotope-labeled unique peptide corresponding to BBX22IR peptide (KVTVLLYLVYDQD) with >98% purity for analysis. The sequence of the heavy isotope-labelled 22IR peptide was confirmed by MALDI-MS and spiked into the trypsin-N digested D4h and L4h protein extracts for parallel reaction monitoring-mass spectrometry (PRM-MS). After experimenting with a series of peptide concentration inputs (5 to 50 femtomole), the concentration of the dissolving reagent (3%-10% acetonitrile with 0.1% formic acid) and the collision energy used (25 to 35), we were still unable to detect the fragmentation ions for the isotope-labeled peptide or the intrinsic BBX22IR peptides in PRM-MS analyses. Other than the endogenous peptide being low in abundance, the synthesized/endogenous peptide is likely less amenable to ionization and fragmentation and therefore undetectable. To exhaust opportunities in detecting this peptide, we adopted a highly sensitive multiple reaction monitoring (MRM) LC-MS approach and first fractionated the proteins into 40 fractions to reduce sample complexities by running through the strong cation exchange column (polySULFOETHYL ATM column), followed by C18 column separation for MRM LC-MS analysis. For optimizing the ionization conditions of the isotope-labeled BBX22IR peptide standard, we tried both an automatic optimization process and manual adjustments with a wide dynamic concentration range from 5 to 100 femtomoles and the collision energies from 25 to 70 to track the predicted top 5 fragmentation ions. Despite the intensive efforts, no promising results were obtained. These efforts also suggest that a much more devoted study would be necessary to identify peptides derived from AS variants. Resources and efforts needed to tackle this challenging project are beyond the scope of this study.*

*The reviewer suggested removing the intron in BBX22 and expressing this construct in the bbx22 mutant for functional assessment. This would be a good alternative if BBX22 and BBX22IR have opposite biological functions, so one could assess the impact of one by eliminating the other. However, our current study indicated that both BBX22 and BBX22IR function as positive regulators of photomorphogenesis (Fig. 7), so the phenotype interpretation by expressing only intronless BBX22 would be unproductive. We acknowledge that removal of the intron as suggested will theoretically reduce the total BBX22 and BBX22IR levels because BBX22 is light-labile whereas BBX22IR lightstable. Regardless, the impact of BBX22IR can be easily masked by its modest level as compared with BBX22. The comparison of the photomorphogenic phenotypes in lines expressing intronless BBX22 and wild-type BBX22 will likely be challenging as well, considering that the BBX22 protein level will naturally vary in different transgenic lines carrying transgenes randomly inserted in the genome. Considering these limitations in reaching unequivocal conclusions, the overexpression of BBX22IR offers a more prominent way to examine the possible contribution of BBX22IR when accumulated to a high level. Although the transcript level of BBX22IR only constitutes ~5% of the BBX22 transcripts according to qRT-PCR, BBX22IR may accumulate to a higher level in specific cells/tissues, at the single cell or at the whole plant level in response to environmental perturbance to exert its biological function. We therefore ask for the reviewer’s kind consideration of our approaches in highlighting the potential functions of BBX22IR in photomorphogenic development.*

*We also appreciate the reviewer for pointing out that the previous title was not inclusive enough for key findings in this study. In this present revision, we have modified the title as “An improved repertoire of splicing variants and their potential roles in Arabidopsis photomorphogenic development”.*

A weakness of the RNA validation experiments is an absence of minus reverse transcriptase controls - see Figures 4d,e; 5c,d,e.

*Response: We thank the reviewer for this suggestion. We have performed new experiments to include controls without reverse transcriptase. The new data are shown in Figs. 4d,e; 5c,d,e of the revised manuscript. Results confirmed that the novel or fusion transcripts can only be detected in the presence of reverse transcriptase.*

Indeed, the authors do not comment on whether some of the many apparently non-coding RNAs they detect could be explained by RT-dependent template switching, which is an established disadvantage of cDNA sequencing. No non-RT-based validation of novel ncRNAs is performed here.

*Response: Indeed RT-dependent template switching could be source of artefacts in cDNA sequencing, especially for the single-exonic transcripts. We believe that the chance that novel transcripts we reported in this study are the result of template switching is relatively low for two major reasons.*

*1.Template switching often results in cDNAs with chimeric sequence context, noncanonical GU-AG splicing junctions and poly(A) sites (PASs) etc. [10-12]. In our case, canonical GU-AG splicing junctions can be detected in all multi-exonic transcripts shown in Fig. 4a and 4e. All of the reported transcripts have typical PASs, correct orientations of 5’ and 3’ adaptor sequences, and none were derived from chimeric transcripts. Also, among the 671 novel transcripts (Fig. 3a), 122 were also detected in datasets generated by Nanopore direct RNA sequencing [5] (new analyses included in the revised manuscript). Our preliminary data of sequencing non-normalized D4h cDNAs with the Nanopore platform also independently confirmed greater than 80% of the D4h novel transcripts reported in this study.*

*2.In the revised manuscript, the validation of the novel and fusion transcripts in Fig. 4d, 4e, 5c, 5d, and 5e was performed with Superscript IV that has much reduced efficiency in template switching activities [13] as compared with the SMARTScribe used for preparing the Iso-seq cDNA libraries. A minus RTase control was also included to ensure that the amplification of these cDNA was not from residual genomic DNA, especially for the single-exonic transcripts.*

*All attempts to validate the novel or fusion transcripts when revising the manuscript reached the same conclusions as drawn previously.*

**Reviewer #2:**

This manuscript entitled "Splicing variants constitute novel regulators of photomorphogenic development in Arabidopsis" presents here a description about normalized cDNA libraries and PacBio Iso-seq to identify new gene models and post-transcriptional regulation. This study also revealed the regulatory function of two IR isoforms, BBX22IR and BBX24IR, in photomorphogenic development. This resource will likely inspire further research for functional characterization of splicing isoforms in photomorphogenic development.

I would like to recommend the following revision.

In this study, normalized cDNA libraries present great advantage for identification of rare isoforms with low abundance. However, normalized cDNA libraries also had great disadvantage in quantitative profiling of isoforms in response to light signals. Thus, common cDNA libraries without normalization for PacBio Iso-seq is highly required for D4h and L4h sample to find light-induced alt-TSSs or alt-TESs, alternative polyadenylation (APA), and Alternative splicing (AS).

*Response: Indeed, cDNA libraries without normalization allow for quantitative analyses. Our study was primarily for a qualitative report by maximizing the detection of splicing variants, therefore adopting a normalization approach.*

*However, we do take the reviewer’s comments seriously and performed two replicates of non-normalized Iso-seq for D4h and L4h samples. The data were compared with the transcriptomes obtained with short-read RNA-seq data from the same developmental stage [6]. The non-normalized Iso-seq datasets showed low presentation for low- and medium-expressed genes and over-representation of the high-expressed genes (Additional file 1: Figure S20a). Differential gene expression fold changes between D4h and L4h were also clearly exaggerated in the non-normalized Iso-seq data (Pearson r=0.453; Additional file 1: Figure S20b). Levels of some of the differentially expression genes were even negatively correlated (Additional file 1: Figure S20b). Even though the non-normalized Iso-seq data reached 1.7-fold sequencing depth as compared with the normalized data, the throughput for this sequencing platform is still likely insufficient for reliable quantitative analyses, as previously described [14-16]. Therefore, we did not pursue the quantitative analyses further with the non-normalized Iso-seq data. Instead, in complementing the qualitative discovery nature of this study, quantitative analyses for light-regulated AS events were conducted by using RNA-seq data that have higher coverage (datasets from a parallel manuscript under revision).*

This manuscript reported "~12,000 new gene models and ~700 unannotated models" by comparing with TAIR10 to classify it into TAIR10 gene models, Iso-seq gene models and TAIR10 unannotated genes. However, the gene models from TAIR10 were outdate. New gene models reported in this study might have been included in published studies using long-read technologies. The authors can compare these new models in this study with up-to-date studies using PacBio SMRT (Cui, Jiawen, et al. "Analysis and comprehensive comparison of PacBio and nanopore-based RNA sequencing of the Arabidopsis transcriptome." Plant Methods 16.1 (2020): 1-13) or Nanopore sequencing (Parker, Matthew T., et al. "Nanopore direct RNA sequencing maps the complexity of Arabidopsis mRNA processing and m6A modification." Elife 9 (2020): e49658.).

*Response: We thank the reviewer for the constructive suggestions. In the revised manuscript, we downloaded and re-analyzed the ~1 M Pacbio Iso-seq datasets using a nonnormalized cDNA library [4] and the ~8 M reads of Nanopore direct RNA sequencing [5]. With the same mapping procedures, 13,493 and 75,096 gene models were generated from these two studies, respectively. The comparison of our data with those from these 2 studies supported 13,284 (72.4%) of our TAIR10 annotated gene models and 1,462 (12.3%) of our TAIR unannotated gene models (Additional file 1: Figure S8 and Additional file 7: Table S6).*

*In addition to comparing to TAIR10, we also compared our data with the more updated Araport11 database, reported in the discussion section of the original manuscript. The comparison revealed that 85% (10,182) of the Iso-seq gene models and 75% (502) of the novel genes were missing in Araport11 (Additional file 12: Table S11 and Additional file 13: Table S12). This finding is similar with the better performance of Nanopore long-read sequencing datasets when compared with Araport11 and AtRTD2 databases in identifying new isoforms [5]. In addition to alternative splicing, we also found that the 5’ end annotation, including alternative transcript start sites/exons, and 3’ end annotation, including alternative transcription end sites/exons and APA sites were severely underestimated in the Araport11 database (Additional file 1: Figure S21 and Additional file 12: Table S11), as described in previous studies [1, 5, 17, 18].*

P5 Line 55 "Therefore, the cDNA normalization allowed for effective detection of rare transcripts." The authors cannot get this conclusion based on only the number of gene loci in this section, since this study did not sequence common cDNA libraries without normalization for PacBio Iso-seq as control. However, next section in the manuscript indeed verified the success of cDNA normalization.

*Response: We concur with the reviewer’s point and moved this conclusion to the next section.*

Figure 3: New models (5787 D4h, 7401 L4h new and 671 TAIR10 unannotated genes) in this studies have great probability to be caused by tissue-specific expression in D4h and L4h, which did not annotated by TAIR10. For this situation, this kind of new model was caused by tissue-specific expression rather than the advantage of PacBio iso-seq long reads or the success of cDNA normalization. Thus Figure 3 can contain control from using published RNA-seq data (Liu, Ming-Jung, et al. "Translational landscape of photomorphogenic Arabidopsis." The Plant Cell 25.10 (2013): 3699-3710.) to reconstruct gene models using transcript assembly software, such as cufflinks or stringtie.

*Response: As suggested, we analyzed the previous RNA-seq data [6] and constructed gene models by using cufflinks based on both TAIR10 and Iso-seq (this study) gene models. Comparisons of gene models from Iso-seq and RNA-seq indicated that ~95% isoforms identified by Iso-seq can be supported by gene models build from RNA-seq data. The results have been included in Additional file 1: Fig. S9 and Additional file 8: Table S7). The analyses also revealed a large number of gene models supported by only RNAseq but not by Iso-seq. Although we cannot rule out that the present Iso-seq has modest sequencing throughput to identify the rare isoforms, it is even more plausible that gene models built based on RNA-seq can not distinguish the real isoforms from those assembled randomly by mixing and matching. This also highlights the importance of identifying splicing isoforms by long-read sequencing.*

*This result also confirms the authenticity of our Iso-seq identified transcript isoforms as included in revised Additional file 6: Table S5.*

Page 8 Line 22 Is it possible that shorter CDS is caused due to the error of PacBio (Figure S5) if the author used PacBio sequencing for CDS prediction?

*Response: Three types of sequencing errors detected in this study were calculated and tabulated in Additional file 1: Figure S5. In the data analysis pipeline, these errors were corrected as described in Methods, and the longest CDSs were used to build gene models. Therefore, we consider that the shorter CDS observed was not a result of sequencing errors but an intriguing feature associated with Iso-seq gene models.*

Page 9 Line 30 RT-PCR revealed several transcripts in etiolated or de-etiolating seedling. The author can present more useful information if they identify and present all novel transcripts in response to light (Differential transcripts between D4h and L4h) using published RNA-seq data (Liu, Ming-Jung, et al. "Translational landscape of photomorphogenic Arabidopsis." The Plant Cell 25.10 (2013): 3699-3710.) or adding common cDNA libraries without normalization for PacBio Iso-seq.

*Response: We thank the reviewer for this suggestion. As suggested, gene expression levels (in RPKM) for the novel transcripts in D4h and L4h samples were calculated from the RNA-seq data [6]. Results showed that 23 novel transcripts were significantly upregulated by 4-h light (p<0.05) and 33 significantly downregulated by 4-h light (p<0.05). These results have been included in Additional file 6: Table S5 of the revised manuscript. We did not pursue the light-regulated gene expression analyses with the non-normalized Iso-seq data because of their skewed expression patterns as elaborated above (analyses shown in Additional file 1: Fig. 20).*

Figure 4d/e: Reference genes such as UBQ should be added in Figure 4d/e to normalize two samples.

*Response: As suggested, we have revised Fig. 4d/e to include the internal control UBQ5.*

From the gene structure in the left panel of Figure 4e, the transcript of IGT00230 generated from single exon gene. Why did RT-PCR present two bands?

*Response: We thank the reviewer for pointing this out. From our Iso-seq and Sanger sequencing results, the IGT00230 consisted of 3 repeated DNA sequences, and the sequences for the first 2 repeats are highly conserved. The forward primer used in PCR can anneal to both repeats; therefore two amplicons (961 bp and 586 bp) were generated in RTPCR. For simplicity, only the longer fragment (961 bp) was shown in revised Fig. 4e.*

Figure 5b: How many AS events were differential between D4h and L4h. RNA-seq from (Liu, Ming-Jung, et al. "Translational landscape of photomorphogenic Arabidopsis." The Plant Cell 25.10 (2013): 3699-3710.) or common cDNA libraries without normalization for PacBio Iso-seq can address this question.

*Response: We performed the differential AS analyses using new RNA-seq data we generated in the lab (a parallel study under revision) by using the DEGSeq R package (1.20.0). By applying a corrected P value (false discovery rate) of 0.005 and log2 transformed fold change of 1, a total of 3,290 differential altA/D events, 4,248 differential IR events and 278 differential exon skipping events were obtained. We believe these two parallel studies offer different and complementary advantages in both qualitative (this study) and quantitative needs of discovering isoforms and quantifying light-regulation of the AS events.*

Page 10, Line 9-10: "254 showed evidence of sequences spanning separately annotated transcripts in TAIR10.". This study only presented two case studies in Figure 5d, e to support the fusion of pair-wise transcripts. The author can provide comprehensive evidence for the 254 fusion transcripts. Using one pair-wise fusion transcripts (At4g32780 and At4g32785) as example, the author may observe that pair-wise fusion transcripts have similar expression profiles due to coordinate expression in different tissues. Thus, the author can calculate expression level (FPKM/RPKM) based RNA-seq from different studies for all the genes. Then Pearson's correlation coefficient (PCC) for each pair of fusion transcripts (such as At4g32780 and At4g32785) can be calculated. If PCC from most of the pair-wise fusion transcripts presents strong bias to positive correlation, which will further support that they indeed originate from fusion transcripts.

*Response: In the revised manuscript, we report 202 instead of 254 fusion transcripts by removing those not from the same transcriptional orientation. We took the very constructive suggestion from the reviewer by downloading > 5,000 RNA-seq datasets from NCBI and calculated the Pearson's correlation coefficient for the expression (in RPKM) of each pair of fusion genes. Coefficients for the neighboring non-fusion genes (n = 19,253) were also calculated and used as a control. As the reviewer suggested, fusion transcripts had significantly higher Pearson's correlation coefficients (r=0.461) than non-fusion transcripts (r=0.155) (one-way ANOVA with unpaired t-test p-value <0.0001). This analysis supported the authenticity of the fusion transcript, and the result has been included in the revised manuscript (Additional File 1: Fig. S12). Two additional pieces of information further supported the authenticity of the fusion transcripts. First, RNA-seq data from the same developmental stage were [6] analyzed for junction reads with ≥10 nt intersected at both genes. Such junction reads were present for 106 fusion transcripts reported in this study (Additional file 9: Table S8). Second, 12 fusion transcripts were annotated in Araport11 (Additional file 13: Table S12).*

Fig. S8a How many APA events were supported by published PacBio Iso-seq (Cui, Jiawen, et al. "Analysis and comprehensive comparison of PacBio and nanopore-based RNA sequencing of the Arabidopsis transcriptome." Plant Methods 16.1 (2020): 1-13), Nanopore DRS (Parker, Matthew T., et al. "Nanopore direct RNA sequencing maps the complexity of Arabidopsis mRNA processing and m6A modification." Elife 9 (2020): e49658.) and PAT-seq (Wu, X., Liu, M., Downie, B., Liang, C., Ji, G., Li, Q. Q., & Hunt, A. G. (2011). Genome-wide landscape of polyadenylation in Arabidopsis provides evidence for extensive alternative polyadenylation. Proceedings of the National Academy of Sciences, 108(30), 12533-12538.) ?

*Response: As suggested, we have included the comparison of the poly(A) sites reported in this study with those reported in 2 of the 3 previous reports, including the Nanopore direct RNA-seq dataset [5] and Pacbio Iso-seq dataset [4]. The PAT-seq was not included because Plant APA database (PlantAPAdb) [3] represented a more inclusive version reported by the same lead author. Results showed >80% poly(A) sites in our study matched or were longer than those annotated in PlantAPAdb, Nanopore or the Pacbio long-read sequencing data. Also, in responding to the suggestion by reviewer 1, we also compared our data with those revealed by the direct RNA sequencing (DRS) datasets [2] and obtained similar results. These results have now been included in Additional file 1: Fig. S13 of the revised manuscript.*

Page 11, line 26-46, in this section, the author only presented 5 IR variants. In total, how many TFs with IR variants including AS events were light induced and were associated with translation machinery?

*Response: In accordance with the reviewer’s comment, we mapped the ribosome-protected fragments reported previously [6] to TF genes included in the Plant Transcription FactorDatabase (http://planttfdb.gao-lab.org/download.php). All 213 TF genes (275 variants) with IR events can have corresponding sequences protected by ribosomes in etiolated or de-etiolating seedlings. Results of this analysis has been included in Additional file 10: Table S9 of the revised manuscript.*

Page 12 44-46 The label of b and c should be switched in Figure7 or main document.

*Response: We have corrected the labeling as pointed out.*

Methods:

The authors cloned FL representative transcripts (short isoforms) for BBX22, BBX24 and their intron retained transcripts (long isoforms) BBX22IR, BBX24IR into vectors. In fact, the retained intron from BBX22IR, BBX24IR included normal GT-AG splicing sites. Thus, overexpressing BBX22IR and BBX24IR line still can generate both retained transcripts and spliced transcripts (short isoforms). The author should check this potential possibility in BBX22IR and BBX24IR overexpressing lines. Mutation the 5′ and 3′ splice sites of the retained intron can only generate intron retained transcripts (long isoforms) without the interference of short isoforms.

The authors should deposit all the PacBio sequencing data in public database (such as NCBI) and cited the accession in manuscript.

*Response: As suggested, all raw data generated in this study have been deposited at the NCBI’s Sequence Read Archive (SRA) under the BioProject number: PRJNA 726863 and SRR accessions: SRR14400342~ SRR14400349 for normalized D4h and L4h datasets, respectively. SRR15010480 and SRR15010481 for non-normalized D4h and L4h datasets, respectively.*

*References*

*1.Schon MA, Kellner MJ, Plotnikova A, Hofmann F, Nodine MD: NanoPARE: parallel analysis of RNA 5' ends from low-input RNA. Genome Res 2018, 28:1931-1942.*

*2.Sherstnev A, Duc C, Cole C, Zacharaki V, Hornyik C, Ozsolak F, Milos PM, Barton GJ, Simpson GG: Direct sequencing of Arabidopsis thaliana RNA reveals patterns of cleavage and polyadenylation. Nat Struct Mol Biol 2012, 19:845-852.*

*3.Zhu S, Ye W, Ye L, Fu H, Ye C, Xiao X, Ji Y, Lin W, Ji G, Wu X: PlantAPAdb: A Comprehensive Database for Alternative Polyadenylation Sites in Plants. Plant Physiol 2020, 182:228-242.*

*4.Cui J, Shen N, Lu Z, Xu G, Wang Y, Jin B: Analysis and comprehensive comparison of PacBio and nanopore-based RNA sequencing of the Arabidopsis transcriptome. Plant Methods 2020, 16:85.*

*5.Parker MT, Knop K, Sherwood AV, Schurch NJ, Mackinnon K, Gould PD, Hall AJ, Barton GJ, Simpson GG: Nanopore direct RNA sequencing maps the complexity of Arabidopsis mRNA processing and m(6)A modification. Elife 2020, 9.*

*6.Liu MJ, Wu SH, Wu JF, Lin WD, Wu YC, Tsai TY, Tsai HL, Wu SH: Translational landscape of photomorphogenic Arabidopsis. Plant Cell 2013, 25:3699-3710.*

*7.Kalyna M, Simpson CG, Syed NH, Lewandowska D, Marquez Y, Kusenda B, Marshall J, Fuller J, Cardle L, McNicol J, et al: Alternative splicing and nonsensemediated decay modulate expression of important regulatory genes in Arabidopsis. Nucleic Acids Res 2012, 40:2454-2469.*

*8.Drechsel G, Kahles A, Kesarwani AK, Stauffer E, Behr J, Drewe P, Ratsch G, Wachter A: Nonsense-mediated decay of alternative precursor mRNA splicing variants is a major determinant of the Arabidopsis steady state transcriptome. Plant Cell 2013, 25:3726-3742.*

*9.Raxwal VK, Simpson CG, Gloggnitzer J, Entinze JC, Guo W, Zhang R, Brown JWS, Riha K: Nonsense-Mediated RNA Decay Factor UPF1 Is Critical for Posttranscriptional and Translational Gene Regulation in Arabidopsis. Plant Cell 2020, 32:2725-2741.*

*10.Cocquet J, Chong A, Zhang G, Veitia RA: Reverse transcriptase template switching and false alternative transcripts. Genomics 2006, 88:127-131.*

*11.Houseley J, Tollervey D: Apparent non-canonical trans-splicing is generated by reverse transcriptase in vitro. PLoS One 2010, 5:e12271.*

*12.Balazs Z, Tombacz D, Csabai Z, Moldovan N, Snyder M, Boldogkoi Z: Templateswitching artifacts resemble alternative polyadenylation. BMC Genomics 2019, 20:824.*

*13.Wulf MG, Maguire S, Humbert P, Dai N, Bei Y, Nichols NM, Correa IR, Jr., Guan S: Non-templated addition and template switching by Moloney murine leukemia virus (MMLV)-based reverse transcriptases co-occur and compete with each other. J Biol Chem 2019, 294:18220-18231.*

*14.Amarasinghe SL, Su S, Dong X, Zappia L, Ritchie ME, Gouil Q: Opportunities and challenges in long-read sequencing data analysis. Genome Biol 2020, 21:30.*

*15.Ardui S, Ameur A, Vermeesch JR, Hestand MS: Single molecule real-time (SMRT) sequencing comes of age: applications and utilities for medical diagnostics. Nucleic Acids Res 2018, 46:2159-2168.*

*16.Hu Y, Shu XS, Yu J, Sun MA, Chen Z, Liu X, Fang Q, Zhang W, Hui X, Ying Y, et al: Improving the diversity of captured full-length isoforms using a normalized singlemolecule RNA-sequencing method. Commun Biol 2020, 3:403.*

*17.Zhang S, Li R, Zhang L, Chen S, Xie M, Yang L, Xia Y, Foyer CH, Zhao Z, Lam HM: New insights into Arabidopsis transcriptome complexity revealed by direct sequencing of native RNAs. Nucleic Acids Res 2020, 48:7700-7711.*

*18.Thieffry A, Vigh ML, Bornholdt J, Ivanov M, Brodersen P, Sandelin A: Characterization of Arabidopsis thaliana Promoter Bidirectionality and Antisense RNAs by Inactivation of Nuclear RNA Decay Pathways. Plant Cell 2020, 32:1845-1867.*

**Second round of review**

**Reviewer 2**

Thanks to the authors for their efforts to improve the manuscript by adding more experiments and bioinformatics analysis. The authors have addressed all my comments in the revised manuscript. The manuscript has been significantly improved.

I checked the point-to-point response in the revised version based on the comment from the other review. I found that most of the concerns from the other reviewer have been addressed. However, the author should consider following two issues.

The author indeed compare their Iso-seq data with nanoPARE datasets. However, the author should add more information for ND type in figure legend (Figure S7) or main document. Otherwise, it's difficult to understand why ND type exists in Iso-seq vs nanoPARE and do not exists in Iso-seq vs TAIR10.

The other reviewer suggested removing the intron in BBX22 and expressing this construct in the bbx22 mutant for detectable impact on phenotype. The authors only explained that BBX22 and BBX22IR function as positive regulators of photomorphogenesis. However, the author did not perform this transgene experiment as suggested. Thus, I am not sure that if this point can satisfy the other review since intrinsic BBX22IR peptides can not be detected.

**Authors Response**

**Point-by-point responses to the reviewers’ comments:**

**Reviewer #2:**

Thanks to the authors for their efforts to improve the manuscript by adding more experiments and bioinformatics analysis. The authors have addressed all my comments in the revised manuscript. The manuscript has been significantly improved.

I checked the point-to-point response in the revised version based on the comment from the other review. I found that most of the concerns from the other reviewer have been addressed. However, the author should consider following two issues.

The author indeed compare their Iso-seq data with nanoPARE datasets. However, the author should add more information for ND type in figure legend (Figure S7) or main document. Otherwise, it's difficult to understand why ND type exists in Iso-seq vs nanoPARE and do not exists in Iso-seq vs TAIR10.

The other reviewer suggested removing the intron in BBX22 and expressing this construct in the bbx22 mutant for detectable impact on phenotype. The authors only explained that BBX22 and BBX22IR function as positive regulators of photomorphogenesis. However, the author did not perform this transgene experiment as suggested. Thus, I am not sure that if this point can satisfy the other review since intrinsic BBX22IR peptides can not be detected.

The manuscript will not be significantly affected if the author did not perform this transgene experiment as suggested since the authors have introduced BBX22IR into *bbx22* mutant background and shown that BBX22IR positively regulated photomorphogenesis inddependent of FL BBX22 (Fig. 7b). This dominant negative role of BBX24IR is common mechanism, which also has been validated by previous studies:

Li, Quanzi, et al. "Splice variant of the SND1 transcription factor is a dominant negative of SND1 members and their regulation in Populus trichocarpa." Proceedings of the National Academy of Sciences 109.36 (2012): 14699-14704

Lin, Ying-Chung Jimmy, et al. "Reciprocal cross-regulation of VND and SND multigene TF families for wood formation in Populus trichocarpa." Proceedings of the National Academy of Sciences 114.45 (2017): E9722-E9729.

*Response: The figure legend of Figure S7 has been further elaborated for clarity.*
